# Recruitment of Cdc48 to chloroplasts by a UBX-domain protein in chloroplast-associated protein degradation

**DOI:** 10.1038/s41477-024-01769-x

**Published:** 2024-08-19

**Authors:** Na Li, R. Paul Jarvis

**Affiliations:** Section of Molecular Plant Biology, Department of Biology, University of Oxford, Oxford, UK

**Keywords:** Cell biology, Protein trafficking in plants, Chloroplasts, Transgenic plants, Proteolysis in plants

## Abstract

The translocon at the outer chloroplast membrane (TOC) is the gateway for chloroplast protein import and so is vital for photosynthetic establishment and plant growth. Chloroplast-associated protein degradation (CHLORAD) is a ubiquitin-dependent proteolytic system that regulates TOC. In CHLORAD, cytosolic Cdc48 provides motive force for the retrotranslocation of ubiquitinated TOC proteins to the cytosol but how Cdc48 is recruited is unknown. Here, we identify plant UBX-domain protein PUX10 as a component of the CHLORAD machinery. We show that PUX10 is an integral chloroplast outer membrane protein that projects UBX and ubiquitin-associated domains into the cytosol. It interacts with Cdc48 via its UBX domain, bringing it to the chloroplast surface, and with ubiquitinated TOC proteins via its ubiquitin-associated domain. Genetic analyses in *Arabidopsis* revealed a requirement for PUX10 during CHLORAD-mediated regulation of TOC function and plant development. Thus, PUX10 coordinates ubiquitination and retrotranslocation activities of CHLORAD to enable efficient TOC turnover.

## Abstract (web summary)

Extraction of ubiquitinated proteins from chloroplasts in CHLORAD is driven by the cytosolic ATPase Cdc48. The UBX-domain protein PUX10 is shown to be a CHLORAD component that recruits Cdc48 to the chloroplast surface.

## Main

Most chloroplast proteins (>90%) are synthesized in the cytosol and imported into chloroplasts post-translationally. The chloroplast protein import machinery consists of two translocons, a translocon located in the outer chloroplast membrane (TOC) and a translocon in the inner chloroplast membrane (TIC). Core components of the TOC are the β-barrel protein, TOC75, and the GTPases TOC159 and TOC33—all named in accordance with their molecular masses in kilodaltons. TOC75 forms a membrane channel for protein conductance, whereas TOC159 and TOC33 function as receptors by binding the transit peptides of precursor proteins via their cytosolic GTPase domains^1–8^.

Chloroplast protein import is dynamically regulated by chloroplast-associated protein degradation (CHLORAD), a ubiquitin-dependent proteolytic system that targets the TOC apparatus^9,10^. By reconfiguring the TOC machinery, CHLORAD action facilitates changes in the organelle’s proteome, functions and morphology. Such CHLORAD-mediated TOC regulation enables the biogenesis and operation of chloroplasts (and of other members of the plastid family of organelles) to be responsive to developmental and environmental cues, including stress^11–13^.

The first characterized CHLORAD component was the ubiquitin E3 ligase suppressor of *ppi* locus 1 (SP1). The SP1 protein is located in the chloroplast outer envelope membrane (OEM) and has a cytosol-facing RING domain and two transmembrane (TM) spans flanking an intermembrane space (IMS) domain that binds to TOC protein targets^9^. Analysis of *sp1*-mutant and SP1-overexpressor *Arabidopsis* plants showed that SP1 expression levels correlate inversely with the abundance of TOC proteins, resulting in the suppression or enhancement of the pale-green *ppi1* (TOC33 knockout)^14^ mutant phenotype. Such manipulation of SP1 expression also has developmental consequences. For instance, the *sp1*-mutant plants showed delayed de-etiolation and leaf senescence, whereas SP1-overexpressor plants showed acceleration of these processes^9,12^.

Other important components of the CHLORAD system are SP2 and Cdc48. The SP2 protein is located in the OEM and, like TOC75, it is a member of the OMP85 superfamily of β-barrel proteins with 16 predicted TM β-strands. This protein was shown to act as a translocon for the retrotranslocation of ubiquitinated TOC proteins^10^. In contrast with SP1 and SP2, Cdc48 is largely located in the cytosol. This protein provides the motive force for extracting TOC components ubiquitinated by SP1 to the cytosol, where degradation by the 26S proteasome occurs, and in this it cooperates with the SP2 membrane channel^10^.

The Cdc48 protein is a member of the ATPases associated with diverse cellular activities (AAA) family and it plays essential functions in a plethora of cellular processes^15^. Its ability to function in so many different pathways depends in part on its adaptor proteins, which control its targeting and activity^16^. For instance, the heterodimeric UFD1–NPL4 complex is a well-characterized adaptor that participates in many ubiquitin-dependent Cdc48-driven processes, including endoplasmic reticulum (ER)-associated protein degradation (ERAD)^17^. Most adaptor proteins possess conserved Cdc48-binding motifs, including ubiquitin regulatory X (UBX), UBXL, SHP, VBM, VIM and PUB^18–20^. Many of these adaptors are not individually essential for cell growth and survival, implying functional redundancies among them. The UBX-domain-containing proteins constitute by far the largest family of Cdc48 adaptor proteins^21^.

The UBX domain comprises approximately 80 amino acid residues and it shares substantial structural similarity with ubiquitin. Proteins possessing the domain are involved in substrate recruitment to Cdc48 and in the temporal and spatial regulation of Cdc48 activity^22–24^. The plant UBX-domain (PUX) proteins define a family of plant proteins that possess a conserved UBX domain for direct interaction with Cdc48. In *Arabidopsis* there are 16 *PUX* genes, most of which are largely uncharacterized^25^. That said, emerging knowledge on the structure and function of PUX proteins provides an indication of their functional links to Cdc48. Several family members appear to serve as adaptors to recruit Cdc48 to specific organelles^26,27^, whereas others are involved in regulating the activity of Cdc48 (refs. ^28–30^). Among the PUX proteins in *Arabidopsis*, PUX10 is the only protein with obvious membrane-anchoring hydrophobic regions. Previous studies identified PUX10 as a lipid droplet (LD)-anchored protein that mediates the degradation of ubiquitinated oleosins during seed germination^31,32^. Intriguingly, PUX10 was also reported to undergo relocalization to chloroplasts during seed maturation^31^.

In this study we investigated the role of PUX10 in chloroplast biogenesis in detail, providing information on the localization, topology, interactions and functions of the protein. On the basis of our results, we conclude that PUX10 is a key part of the CHLORAD machinery for TOC protein degradation. The UBX domain of PUX10 recruits cytosolic Cdc48 to the chloroplast surface, while its ubiquitin-associated (UBA) domain binds to ubiquitinated TOC proteins, thereby bringing them into close proximity with Cdc48 for retrotranslocation.

## Results

### Localization and topology analysis of the PUX10 protein

**Fig. 1:**
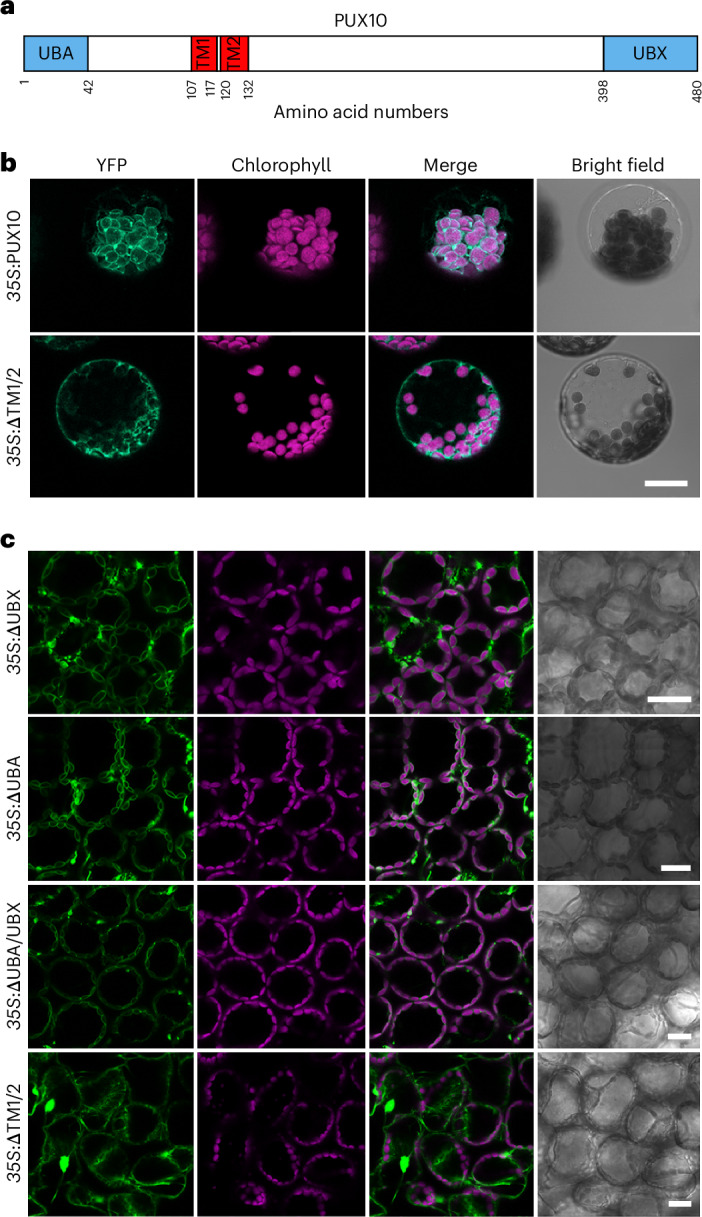
The hydrophobic region of PUX10 is essential for PUX10 localization to chloroplasts. **a**, Protein domain map for PUX10 showing its UBA, potential TM and UBX domains. **b**, Analysis of PUX10 localization upon transient expression. Protoplasts transiently expressing different YFP-tagged PUX10 variants (under the control of the *35S* promoter) were analysed by confocal microscopy. Representative protoplasts are shown. Exposure times and gain settings were identical in each case. Localization of PUX10–YFP to the chloroplast envelope (top) depended on the TM domains, as revealed by a double-TM deletion mutant (bottom). Scale bar, 20 µm. **c**, Analysis of PUX10 localization in transgenic plants. Constructs encoding different variants of PUX10 lacking the indicated domains (under the control of the *35S* promoter) were used to stably transform *Arabidopsis* plants. Rosette leaves taken from 28-day-old T_1_ plants were visualized by confocal microscopy. Representative images are shown as in **b**. Similar localization of the YFP signals was observed in 5–10 independent T_1_ transgenic plants. Exposure times and gain settings were identical. Scale bars, 20 µm.

**Extended Data Fig. 1:**
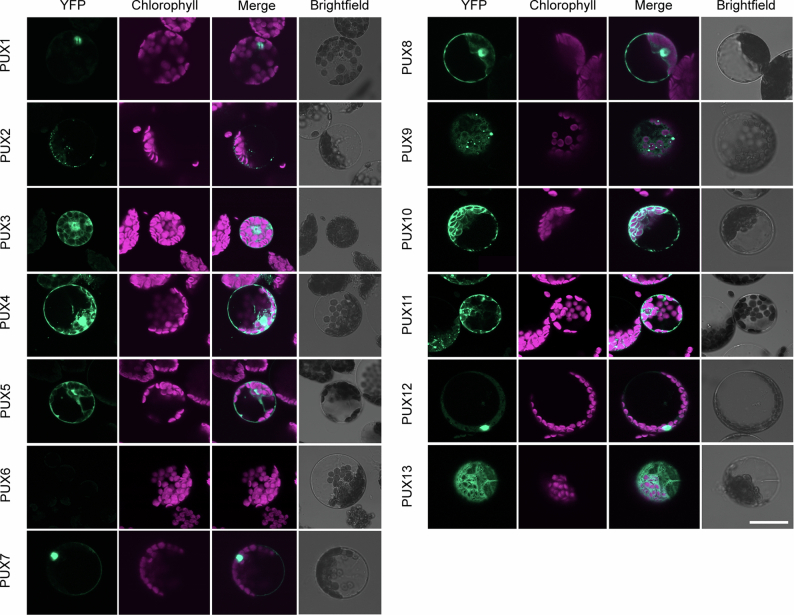
Subcellular localization analysis of *Arabidopsis* PUX proteins by confocal microscopy. Protoplasts transiently expressing YFP-tagged PUX proteins under the constitutive *35S* promoter were analysed by confocal microscopy. Representative protoplasts are presented. Exposure times and gain settings were identical. Scale bar = 20 µm. Note that *PUX14*, *PUX15* and *PUX16* were designated as pseudogenes in a previous report, based on the presence of frameshift and nonsense mutations, and so were excluded from this analysis^26^.

**Extended Data Fig. 2:**
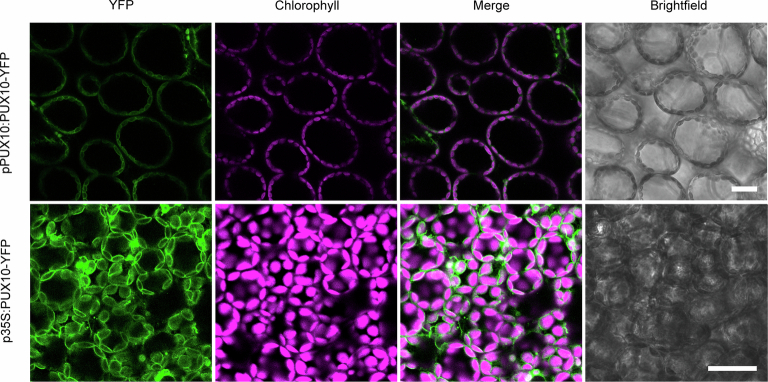
Localization of PUX10 in chloroplasts in transgenic plants. Constructs encoding PUX10-YFP driven by the native *PUX10* promoter (*pPUX10*) or by the constitutive *35S* promoter (*p35S*) were used for stable plant transformation. Rosette leaves taken from 28-day-old T_1_ transgenic plants were visualized by confocal microscopy. Representative images are presented. Similar localization of the YFP signals was observed in 5 to 10 independent T_1_ transgenic plants. Exposure times and gain settings were identical. Scale bars = 20 µm.

With the aim of identifying Cdc48 adaptors that act in CHLORAD, we conducted a subcellular localization screening analysis of all expressed PUX proteins in *Arabidopsis*. In this analysis, PUX10 was the only PUX protein showing distinct association with chloroplasts (Extended Data Fig. 1). The PUX10 protein has two predicted TM spans, an amino-terminal (N-terminal) UBA domain, and a carboxy-terminal (C-terminal) UBX domain (Fig. 1a). Wishing to understand the function of PUX10, we began by studying its localization in greater detail. To investigate the role of the two predicted TM domains in the localization of PUX10, a truncated PUX10 variant lacking the TM domains (ΔTM1/2) was generated. Both intact and deleted versions of PUX10 were fused with yellow fluorescent protein (YFP) and transiently expressed in protoplasts. Confocal visualization indicated that the full-length protein was localized in chloroplasts (in the chloroplast envelope) and that the truncated version was located in the cytosol (Fig. 1b). This indicated that PUX10 shows chloroplast envelope membrane localization dependent on its TM domains. To corroborate this conclusion, stable transgenic plants expressing various PUX10 forms fused to YFP were generated and analysed. This analysis indicated that the TM domains alone contribute to the chloroplast localization of PUX10; deletion of the UBA and UBX domains did not affect its localization (Fig. 1c). Lastly, we compared transgenic plants expressing the full-length PUX10–YFP fusion under the control of native or strong constitutive (*35S*) promoters, again viewing the localization patterns by confocal microscopy (Extended Data Fig. 2). Similar results were obtained, indicating that neither the expression system nor the expression level had a major effect on the predominant subcellular localization of PUX10. Collectively, these results showed that the TM domains of PUX10 are responsible for anchoring the protein in the chloroplast envelope membrane in leaves.

**Fig. 2:**
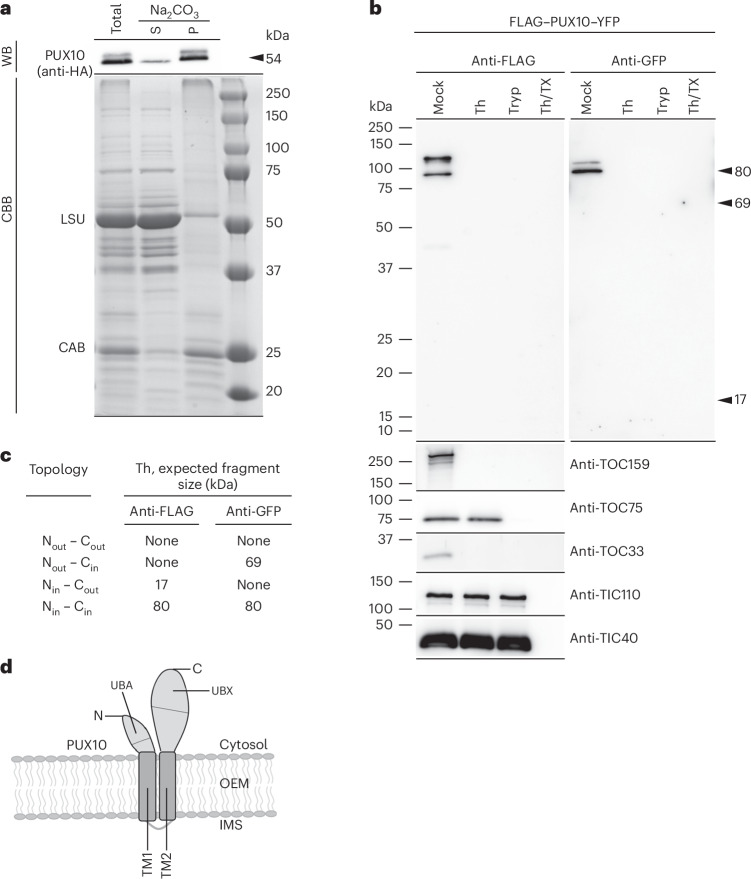
Topological analysis of PUX10 reveals cytosolic orientation of both termini. **a**, Alkaline extraction analysis of PUX10. Chloroplasts isolated from transgenic plants expressing PUX10–HA were fractionated into membrane pellet (P) and soluble supernatant (S) fractions after high-pH (Na_2_CO_3_) washing. The samples were analysed by anti-HA immunoblotting to detect the PUX10–HA protein (western blotting, WB; top) and by Coomassie brilliant blue staining (CBB; bottom). Endogenous marker proteins (RuBisCo large subunit (LSU); chlorophyll *a*/*b* binding proteins (CAB)) partitioned as expected. **b**,**c**, Protease protection analysis of the PUX10 protein. **b**, chloroplasts isolated from transgenic plants overexpressing FLAG–PUX10–YFP were subjected to treatment using thermolysin (Th), trypsin (Tryp), thermolysin plus Triton X-100 (Th/TX) or buffer lacking protease (mock). Immunoblotting using antibodies to the FLAG and YFP tags was conducted to assess the protease accessibility of the protein termini. The arrowheads indicate the positions of protected proteolytic fragments that would be expected after thermolysin protease treatment if alternative PUX10 topologies exist. Separate immunoblot analysis of five endogenous marker proteins (TOC and TIC components), using the same samples, confirmed the efficiency of the protease treatments. **c**, four possible topologies of the PUX10 protein in the OEM can be envisaged. The expected sizes of protected fragments for each possible topology following thermolysin treatment are shown; the positions of these are indicated in **b** with arrowheads. **d**, Schematic drawing showing the topology of PUX10 according to the protease treatment results. Source data

Next, to investigate the topology of the PUX10 protein, we generated transgenic plants expressing a PUX10–HA construct, with an HA tag fused to the C terminus of PUX10. First, alkaline extraction was applied to determine whether PUX10 is a peripheral or integral membrane protein. Chloroplasts isolated from the transgenic plants were treated with 100 mM Na_2_CO_3_ (pH 11.5) to remove non-integrated proteins from the membranes. Then, membrane pellet and soluble fractions were recovered and analysed alongside a total chloroplast sample by immunoblotting. Almost all of the PUX10–HA protein was found in the membrane pellet fraction, with very little in the soluble fraction, indicating that PUX10 is indeed an integral chloroplast membrane protein (Fig. 2a).

On the basis of the presence of two predicted TM spans in PUX10 and the similarity of its overall domain architecture to that of Ubx2, a yeast homologue^33^, it was hypothesized that the topology of PUX10 is such that both the UBA and UBX domains face the cytosol, enabling the protein to access both ubiquitinated substrates and Cdc48, as Ubx2 does in yeast. However, such a topological arrangement of PUX10 had not previously been assessed experimentally. To do this, we generated transgenic plants expressing a FLAG–PUX10–YFP construct, with FLAG and YFP tags fused to the N terminus and C terminus of PUX10, respectively. A protease protection assay using isolated chloroplasts and thermolysin or trypsin proteases was used (Fig. 2b). Thermolysin cannot penetrate beyond the OEM, which means it will digest only those OEM proteins that are exposed at the organelle surface (that is, normally facing the cytosol) without causing major damage to the integrity of the chloroplast^34^. In contrast, trypsin can partially disrupt the integrity of the OEM and thus gain access to the IMS, where it may digest regions of OEM or inner envelope membrane (IEM) proteins that extend into the IMS^35,36^.

Thus, chloroplasts were isolated from the FLAG–PUX10–YFP transgenic plants and either mock treated or treated with thermolysin or trypsin (the former with or without Triton X-100 detergent to disrupt the chloroplast membranes, as a control). All samples were analysed by immunoblotting using anti-FLAG or anti-GFP sera, or antisera against the following control proteins: TOC159 and TOC33 (which are OEM proteins with large cytosolic domains and were sensitive to thermolysin treatment as expected), and TOC75, TIC110 and TIC40 (which are deeply embedded in the OEM or in the IEM and were resistant to thermolysin and/or trypsin treatment as expected) (Fig. 2b). The full-length FLAG–PUX10–YFP fusion protein (~80 kDa) was detectable using both tags in the absence of protease treatment. However, both the N terminus (FLAG tagged) and the C terminus (YFP tagged) of PUX10 showed strong sensitivity to thermolysin, implying that PUX10 is located in the OEM with both termini facing the cytosol. Indeed, protected fragments that would be expected for alternative topological arrangements were absent (Fig. 2b,c) and complete degradation of PUX10 by trypsin treatment further supported this conclusion. Altogether, these results provided direct evidence for the OEM localization of PUX10, for the existence of two TM spans in the N-terminal hydrophobic region of PUX10, and for the cytosolic orientation of both the UBA and UBX domains, ensuring their accessibility to ubiquitinated substrates and Cdc48, respectively (Fig. 2d).

### PUX10 recruits Cdc48 to the chloroplast envelope

**Extended Data Fig. 3:**
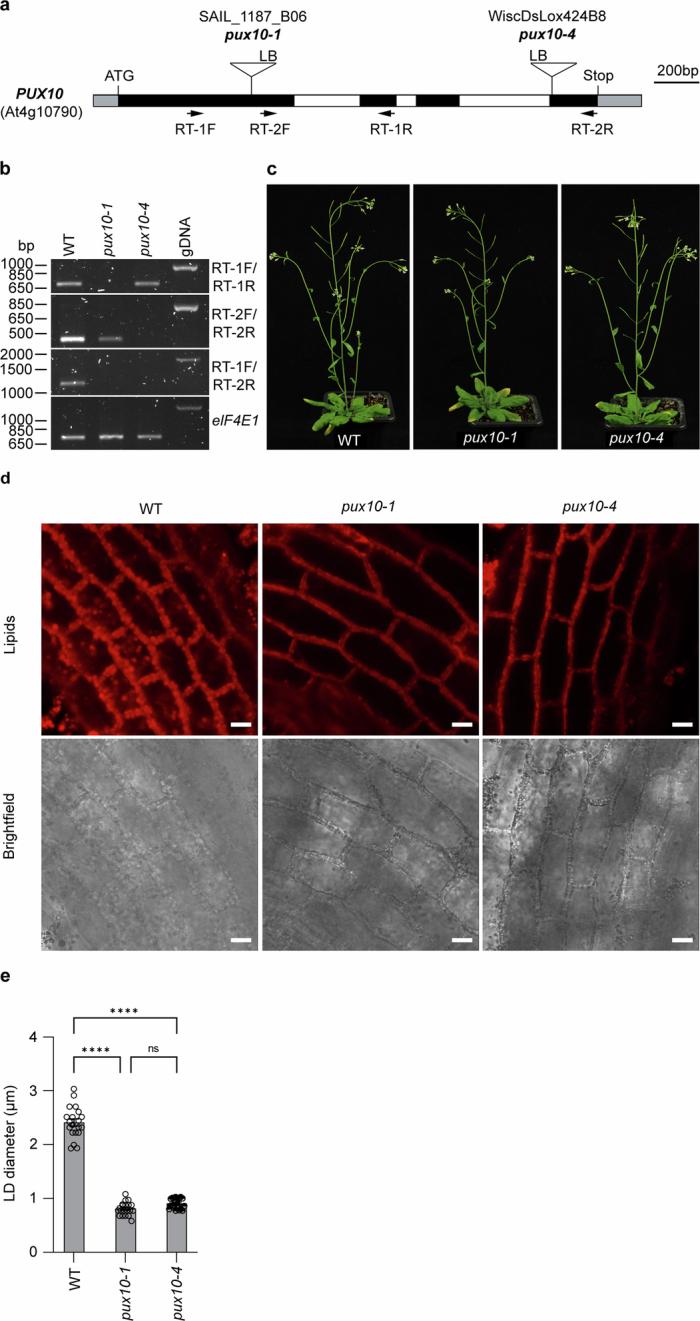
Molecular and phenotypic characterization of two *pux10* T-DNA insertion mutants. **a**, Schematic representation of the *Arabidopsis PUX10* genomic locus (At4g10790), annotated with the positions of the *pux10* T-DNA insertion mutations. The positions of PCR primers used in **b** are also indicated, with arrows. Black boxes show exons, interconnecting white boxes show introns, and grey boxes show untranslated regions. Abbreviations: LB, left border sequences of the SAIL and Wisconsin T-DNA insertions; ATG, translation initiation codon; Stop, translation termination codon; bp, base pairs. **b**, Analysis of *PUX10* mRNA expression in the *pux10* mutants and a corresponding wild-type control. Total RNA isolated from 3-week-old seedlings was analysed by RT-PCR using the indicated primers (primer positions are marked in a, and their sequences are listed in Supplementary Table 1). The *eIF4E1* gene was similarly analysed as a control for sample normalization. Wild-type genomic DNA (gDNA) was analysed as a positive control, and to rule out possible DNA contamination of the samples. Amplifications employed a limited number of cycles, and products were analysed by agarose gel electrophoresis. **c**, Phenotypes of 6-week-old wild-type, *pux10-1* and *pux10-4* plants grown on soil. Representative individuals are shown. Identical camera settings were employed, and all images are at the same magnification. **d**,**e**, Lipid droplet size in wild-type, *pux10-1* and *pux10-4* seedlings. Based on previous results^31^, we compared the size of lipid droplets (LDs) at the young seedling stage (34 h of germination) in the different plant genotypes. **d**, LDs were stained with Nile Red and visualized in the hypocotyl epidermis by confocal microscopy. Representative Nile Red fluorescence and brightfield images are shown. Exposure times and gain settings were identical. Scale bar = 10 µm. **e**, Quantification of LD size in wild-type, *pux10-1* and *pux10-4* plants. Images presented in **d**, and other similar images, were analysed. Values shown are means ± s.e.m. from 20 LDs per genotype. Asterisks indicate significance according to an unpaired two-tailed Student’s *t*-test (****P < 0.0001; ns, not significant). The two *pux10* mutants showed significantly and similarly reduced LD size compared to wild type, supporting the conclusion that the two *pux10* alleles are equivalent. Source data

To further understand the function of PUX10, two transfer-DNA (T-DNA) insertion mutants were obtained: *pux10-1* (SAIL-1187-B06) and *pux10-4* (WiscDslox424B8) (Extended Data Fig. 3a). On the basis of reverse transcription–polymerase chain reaction (RT-PCR) analysis of *PUX10* expression, both *pux10-1* and *pux10-4* were considered to be null mutants of *PUX10* (Extended Data Fig. 3b). However, there were no obvious phenotypic differences between the mutants and wild-type (WT) plants under standard growth conditions (Extended Data Fig. 3c). As the two *pux10* mutants are phenotypically identical (Extended Data Fig. 3d,e)^31^, the data presented hereafter are for *pux10-1* only as a representative allele.

**Fig. 3:**
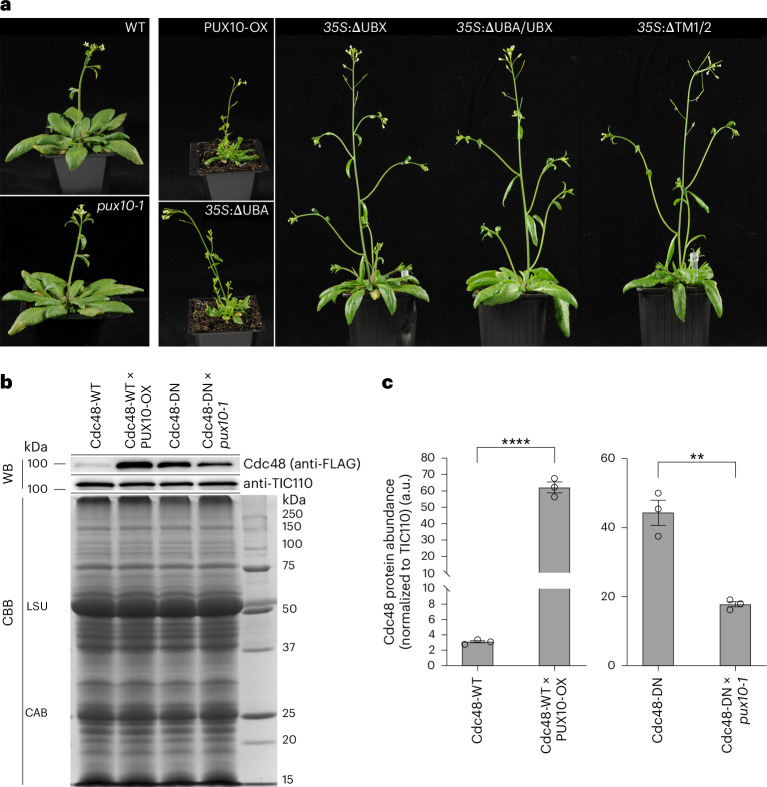
PUX10 influences the subcellular distribution of Cdc48 and plant growth. **a**, Phenotypes of WT, *pux10-1* and transgenic plants, the latter expressing various forms of the PUX10 protein under the constitutive *35S* promoter. Representative individuals are shown. Identical camera settings were used and all images are at the same magnification. Two *pux10* alleles were phenotypically identical and so only *pux10-1* is presented here as a representative allele. The WT and *pux10-1* plants were 5 weeks old and all the overexpression or *35S* lines were 6 weeks old (see Extended Data Fig. 4 for 3-week-old WT, *pux10* (two alleles) and transgenic plants). **b**,**c**, The extent of Cdc48 localization to chloroplasts depends on the expression level of PUX10. **b**, chloroplasts isolated from plants expressing different FLAG-tagged Cdc48 variants (either WT or DN) in different genetic backgrounds (either PUX10-OX or *pux10-1*) were analysed by immunoblotting after verification that the genetic background did not influence total FLAG-tagged protein expression. Anti-FLAG antibody was used to detect Cdc48, and TIC110 was analysed as an endogenous loading control. An equivalent Coomassie-brilliant-blue-stained gel was prepared to show equal loading of the samples. Cdc48-DN showed significantly enhanced chloroplast association relative to Cdc48-WT, which showed weak chloroplast association, consistent with published data^10^. **c**, quantification of the changes in abundance of chloroplast-associated Cdc48 in the PUX10-OX (left) or *pux10-1* (right) backgrounds was performed. Band intensities were quantified and normalized to corresponding TIC110 data; the data are presented relative to the relevant control genotype in arbitrary units. Values shown are means ± s.e.m. from three biological replicates. Asterisks indicate significance according to an unpaired two-tailed Student’s *t*-test. ^**^P = 0.0020, ^****^P < 0.0001. Source data

**Extended Data Fig. 4:**
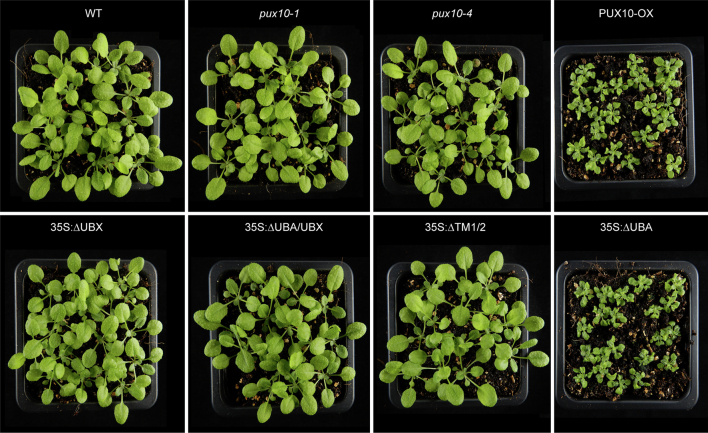
Visible appearance of wild-type, *pux10* mutant, and transgenic plants. Plants were grown on soil under standard conditions for 3 weeks before photography, and representative individuals are shown. Identical camera settings were employed, and all images are at the same magnification.

Although the *pux10*-knockout mutants appeared phenotypically normal, transgenic plants overexpressing full-length PUX10 (PUX10-OX) were severely dwarfed (Fig. 3a and Extended Data Fig. 4). In addition to the plants overexpressing intact PUX10, lines expressing truncated PUX10 forms were also generated and these showed different phenotypes. Transgenic lines with the UBX domain deleted (ΔUBX), both UBX and UBA domains deleted (ΔUBX/UBA) and both TM domains deleted (ΔTM1/2) all showed similar phenotypes to the WT. However, the same dwarfism as seen for full-length PUX10 overexpression was observed in the transgenic plants with the UBA domain deleted (ΔUBA; Fig. 3a). These phenotypic differences implied that overexpression of PUX10 can have a dominantly acting negative effect on plant growth and that both the UBX and TM domains of PUX10 are essential for this effect to be mediated.

Given that UBX is a Cdc48-binding domain and that Cdc48 has a wide spectrum of activity in various organelles and compartments, one can hypothesize that the dwarfism observed in both full-length PUX10 overexpression and ΔUBA-expressing plants was due to greatly disrupted subcellular distribution of Cdc48 due to the high-level expression of these UBX-domain proteins. It is noteworthy that no dwarfism was observed in ΔTM1/2 transgenic plants, implying that the expression of this cytosolic UBX-domain protein does not similarly disrupt the distribution of Cdc48. The selective overaccumulation of Cdc48 on chloroplasts via the UBX domain of PUX10, or its depletion from the cytosol or other compartments as a consequence, might be responsible for the developmental aberrancy seen in these transgenic plants.

To address this hypothesis, we made use of oestradiol-inducible Cdc48-WT–FLAG and Cdc48-DN–FLAG constructs^10^, which were introduced into PUX10-OX and *pux10* backgrounds, respectively, via genetic crossing. The Cdc48-WT protein shows only weak association with chloroplasts, whereas the Cdc48-DN protein, which is a dominant-negative (DN) mutant with stabilized substrate binding, shows more stable association with chloroplasts. Chloroplasts were isolated from the resulting transgenic plants and analysed by immunoblotting using anti-FLAG antibody to assess the extent of chloroplast association of Cdc48 (Fig. 3b,c). We observed that the weaker chloroplast-localized signal for Cdc48-WT–FLAG was significantly enhanced in the PUX10-OX background, whereas the strong chloroplast-localized signal for Cdc48-DN–FLAG was conversely reduced in the *pux10*-knockout background. This result indicated that the expression of PUX10 has a strong influence on the accumulation of Cdc48 at chloroplasts.

### PUX10 interacts with Cdc48 via its UBX domain

**Fig. 4:**
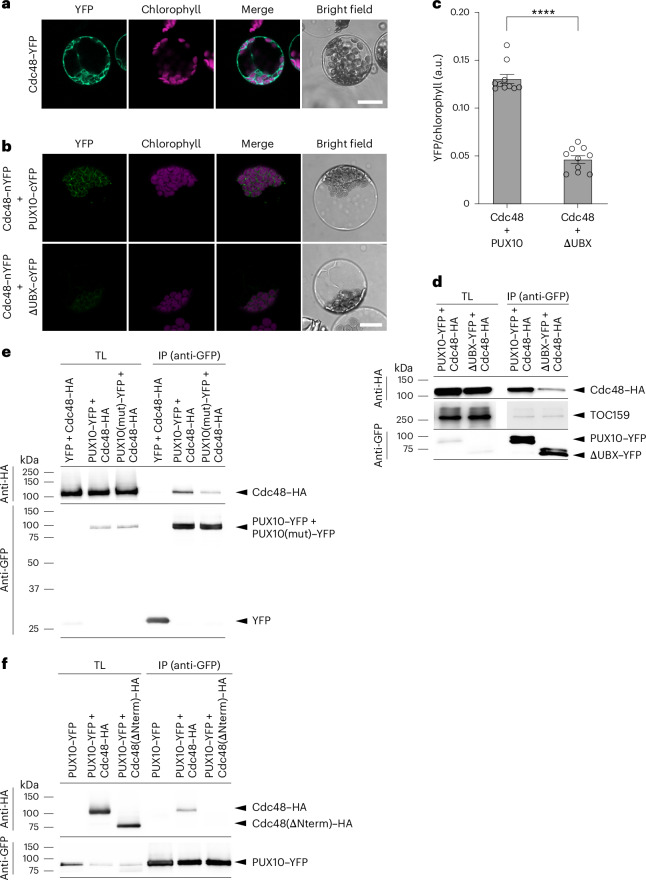
PUX10 interacts with Cdc48 at the chloroplast surface via its UBX domain. **a**, Localization analysis of the Cdc48 protein. Protoplasts transiently expressing a Cdc48–YFP construct were analysed by confocal microscopy. A representative protoplast is shown. Scale bar, 20 µm. **b**,**c**, BiFC analysis of the interaction between PUX10 and Cdc48. **b**, protoplasts co-expressing proteins fused to nYFP or cYFP fragments of YFP were visualized by confocal microscopy. Protoplasts showing typical results are shown. Exposure times and gain settings were identical. Scale bar, 20 µm. **c**, relative intensities of the BiFC signals were quantified and normalized with respect to chlorophyll autofluorescence. Each measurement was of a different field of view area; each area contained ~40 protoplasts. The values shown are means ± s.e.m. from ten measurements. Asterisks indicate significance according to an unpaired two-tailed Student’s *t*-test. ^***^P < 0.0001. **d**, Co-IP analysis of the interaction between PUX10 and Cdc48. Protoplasts transiently co-expressing the indicated proteins were solubilized and subjected to anti-GFP co-IP analysis. Anti-GFP immunoblot analysis verified the enrichment of the PUX10–YFP or ΔUBX–YFP proteins, whereas anti-HA analysis assessed co-purification of Cdc48–HA. Endogenous TOC159 protein was detected using anti-TOC159 antibody. **e**, Co-IP analysis of the interaction between Cdc48 and PUX10 with a UBX-domain triple-point mutation. Protoplasts transiently co-expressing the indicated proteins were solubilized and subjected to anti-GFP co-IP analysis. Anti-GFP immunoblot analysis verified the enrichment of the PUX10–YFP or PUX10(mut)–YFP proteins, whereas anti-HA analysis assessed co-purification of Cdc48–HA. **f**, Co-IP analysis of the interaction between PUX10 and Cdc48 lacking its N terminus. Protoplasts transiently co-expressing the indicated proteins were solubilized and subjected to anti-GFP co-IP analysis. Anti-GFP immunoblot analysis verified the enrichment of the PUX10–YFP proteins, whereas anti-HA analysis assessed co-purification of Cdc48–HA or Cdc48(∆Nterm)–HA. TL, total lysate. Source data

Proteins with a UBX domain have been shown to recruit Cdc48 to specific subcellular locales or organelles via direct interaction between the UBX domain and the N-terminal domain of Cdc48 (ref. ^21^). To investigate whether this may be the case for PUX10, we performed bimolecular fluorescence complementation (BiFC) assessments using the pSATN BiFC system^37^, in which the YFP variant EYFP (Clontech) is split between amino acid residues 174 and 175 to yield complementary N-terminal (nYFP) and C-terminal (cYFP) fragments. Full-length PUX10 and ΔUBX (PUX10 with the UBX-domain deleted) were fused with cYFP, and Cdc48 was fused with nYFP. The resulting constructs encoding complementary nYFP and cYFP fragments were co-expressed in pairs in *Arabidopsis* protoplasts. Meanwhile, as a control, we also transfected protoplasts with a single construct encoding Cdc48 fused to full-length YFP. Subsequent confocal microscopy analysis indicated that the Cdc48–YFP protein, expressed alone, is located predominantly in the cytosol (Fig. 4a). In the BiFC analysis, several key observations were made (Fig. 4b,c). First, fluorescence signals were observed when Cdc48 and full-length PUX10 fusions were co-expressed, indicating that these two proteins can interact. Second, these BiFC signals were localized to the chloroplast envelope membrane, supporting the view that PUX10 mediates the relocalization of Cdc48 to the chloroplast surface. Third, the detected interaction depended on the UBX domain of PUX10 because the BiFC signals were significantly reduced when ΔUBX was used instead of the full-length PUX10 protein.

To corroborate the findings of the BiFC analysis, co-immunoprecipitation (co-IP) experiments were performed. Constructs encoding either full-length PUX10 or the ΔUBX variant fused to YFP (that is, PUX10–YFP or ΔUBX–YFP) were co-expressed in protoplasts along with a previously described construct encoding HA-tagged Cdc48 (Cdc48–HA)^10^. Here, PUX10–YFP and ΔUBX–YFP were used as the bait proteins and were immunoprecipitated with anti-GFP beads after solubilization, while Cdc48–HA acted as the prey (Fig. 4d). As expected, the results clearly indicated an interaction between full-length PUX10 and Cdc48. However, this interaction was strongly disrupted when ΔUBX was used, which is consistent with the observations from the BiFC analysis.

**Extended Data Fig. 5:**
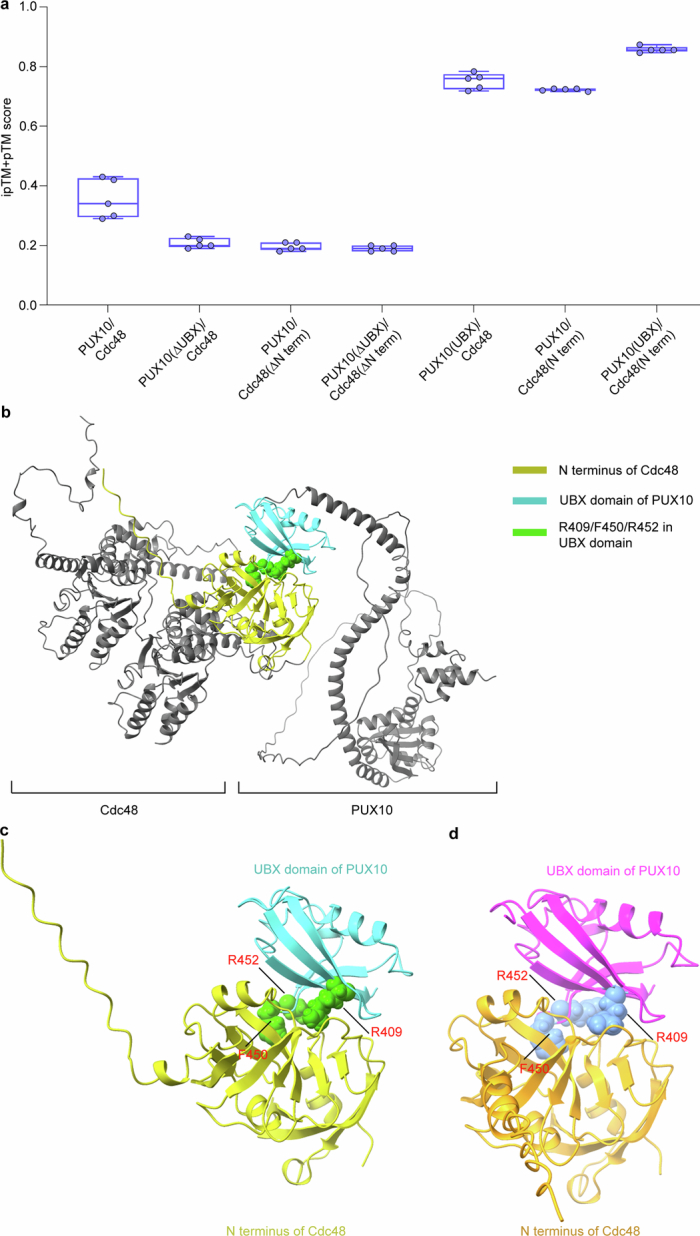
AlphaFold analysis of the interaction between PUX10 and Cdc48. **a**, Analysis of the interaction between PUX10 and Cdc48 using full-length or deleted forms of the proteins, or isolated domains, using AlphaFold. Box plots show the ipTM+pTM scores for each of five models for each of the protein pairs, and provide an indication of the likelihood of the relevant interaction. In each case, the box spans the interquartile range, the whiskers indicate the minimum and maximum scores, and the line inside the box represents the median. **b**, Structural model from the AlphaFold prediction of the interaction between the two full-length proteins. Yellow highlights the N terminus of Cdc48 while blue highlights the UBX domain of PUX10; the three residues of the highly-conserved R…FPR surface patch in the UBX domain are shown as green spheres. Outside of the highlighted domains, both Cdc48 and PUX10 are displayed in grey. **c**, Higher magnification image of the Cdc48-PUX10 interaction interface shown in **b**. Three residues of the highly-conserved R…FPR surface patch in the UBX domain are labelled in red. **d**, Structural model from the AlphaFold prediction of the interaction between the UBX domain of PUX10 and the N terminus of Cdc48 (that is, isolated domains). Orange highlights the N terminus of Cdc48 while magenta highlights the UBX domain of PUX10; the three residues of the highly conserved R…FPR surface patch in the UBX domain are shown as blue spheres and are labelled in red.

Next, we used AlphaFold^38,39^ to analyse the interaction between PUX10 and Cdc48 in silico. The analysis provides two intrinsic model-accuracy estimates (predicted template modelling (pTM) and interface pTM (ipTM)) and we used a combination of these two estimates as the confidence metric. An ipTM + pTM score of 0.5 or more is considered to be indicative of a reliable interaction^39,40^. Although analysis of a polypeptide pair comprising full-length PUX10 and full-length Cdc48 scored slightly below 0.5, it did score noticeably higher than pairs including one or both of the proteins in truncated form (that is, PUX10 lacking the UBX domain and Cdc48 lacking the N terminus) (Extended Data Fig. 5a). The low score associated with the analysis of the full-length proteins might have been related to a lack of similar structures in the training data, the presence of disordered or flexible regions or difficulty in modelling the interactions of large multi-domain proteins. Indeed, when polypeptide pairs including one or both of the domains of interest in isolated form were analysed, scores in excess of 0.75 were obtained (Extended Data Fig. 5a). Inspection of the predicted three-dimensional (3D) folds of the different interaction pairs showed that the structural arrangement at the interaction interface was highly similar regardless of whether full-length proteins or isolated UBX and N domains were used (Extended Data Fig. 5b–d). Thus, overall, these data are strongly supportive of the hypothesis that PUX10 and Cdc48 interact directly via their UBX and N domains.

**Extended Data Fig. 6:**
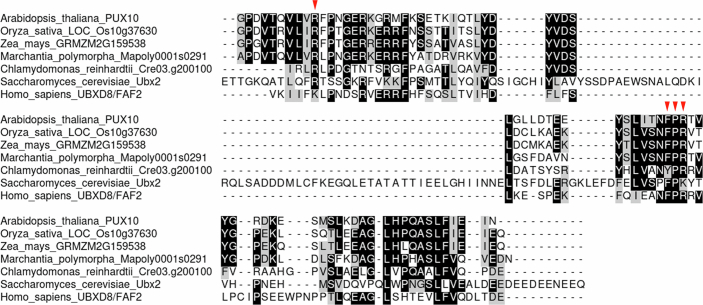
UBX domain alignment of selected PUX10 homologues. Amino-acid sequence alignment of the UBX domains of PUX10 and PUX10/Ubx2 homologues from different species (as indicated). Black highlights identical amino acids, while grey highlights amino acids that are similar. Residues of the highly conserved R…FPR surface patch of the UBX domain are marked with red arrowheads.

The UBX domain has a β-β-α-β-β-α-β secondary structure^41^. An exposed arginine residue in strand 1 and an FPR motif in the loop connecting strands 3 and 4 form a highly conserved surface patch (R…FPR, where the ellipsis represents intervening residues) (Extended Data Fig. 6). This R…FPR motif was found to be the major binding site of the UBX domain and its mutation greatly reduced its Cdc48/p97 binding^42^. To address whether the R…FPR surface patch of PUX10 is important for the Cdc48–PUX10 interaction, we generated a mutant PUX10 (PUX10(mut)) with a triple-point mutation in the R…FPR motif (R409A, F450S and R452A). Constructs encoding either WT PUX10 or PUX10(mut) fused to YFP (that is, PUX10–YFP or PUX10(mut)–YFP) were co-expressed in protoplasts along with a previously described construct encoding HA-tagged Cdc48 (Cdc48–HA)^10^. In parallel, a free YFP construct was co-expressed with Cdc48–HA to serve as a negative control. Here PUX10–YFP and PUX10(mut)–YFP were used as the bait proteins and were immunoprecipitated with anti-GFP beads after solubilization, while Cdc48–HA acted as the prey (Fig. 4e). As expected, the results clearly showed that the binding of PUX10 to Cdc48 was substantially reduced by the triple-point mutation. Therefore, the conserved R…FPR surface patch is essential for PUX10 binding to Cdc48.

It is well known that most Cdc48 adaptor proteins, including UBX proteins, bind to the N-terminal domain of Cdc48 (ref. ^19^). Indeed, our predictions by AlphaFold pointed to an interaction between the PUX10 UBX domain and the Cdc48 N terminus (Extended Data Fig. 5). To corroborate the prediction from AlphaFold, co-IP analysis was performed. Constructs encoding either full-length Cdc48 or Cdc48 lacking the N terminus (that is, Cdc48–HA or Cdc48(∆Nterm)–HA) were co-expressed in protoplasts along with the construct encoding PUX10–YFP. Here PUX10–YFP was used as the bait protein and was immunoprecipitated with anti-GFP beads after solubilization, while Cdc48–HA and Cdc48(∆Nterm)–HA acted as the prey (Fig. 4f). As expected, the binding of Cdc48 to PUX10 was abolished by the truncation of the N terminus.

Taken together, these results showed that PUX10 is able to bind the N terminus of Cdc48 through its UBX domain to recruit Cdc48 to the chloroplast envelope membrane.

### PUX10 interacts with the CHLORAD machinery

The Cdc48 ATPase was previously shown to form a complex with SP1 and SP2 at the surface of the chloroplast, enabling the ubiquitin-dependent degradation of TOC proteins by the cytosolic 26S proteasome in CHLORAD^10^. To investigate the possibility that PUX10 is involved in these processes, we assessed whether PUX10 also associates with SP1 and SP2.

**Fig. 5:**
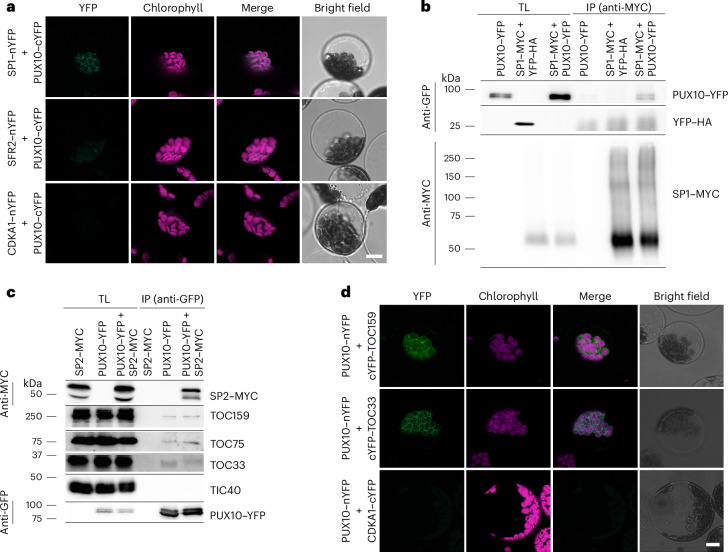
PUX10 interacts with SP1, SP2 and TOC proteins. **a**, BiFC analysis of the interaction between PUX10 and SP1. Protoplasts transiently co-expressing the SP1 and PUX10 proteins fused to nYFP and cYFP fragments of YFP, respectively, were analysed by confocal microscopy. In parallel, SFR2 was used as a chloroplast-localized negative control and CDKA1 was used as a cytosol-localized negative control. Representative protoplasts are shown. Exposure times and gain settings were identical. Scale bar, 10 µm. **b**, Co-IP analysis of the interaction between PUX10 and SP1. Protoplasts expressing the indicated proteins were solubilized and subjected to anti-MYC co-IP analysis. Anti-MYC tag immunoblot analysis verified the enrichment of the SP1–MYC protein and anti-GFP analysis assessed co-purification of PUX10–YFP or YFP–HA. **c**, Co-IP analysis of the interaction between PUX10 and SP2 or TOC proteins. Protoplasts expressing the indicated proteins were solubilized and subjected to anti-GFP co-IP analysis. Anti-GFP tag immunoblot analysis verified the enrichment of the PUX10–YFP protein and anti-MYC analysis assessed co-purification of SP2–MYC. Further immunoblot analysis using antibodies to key TOC components was used to detect co-purification of endogenous TOC proteins; similar analysis of TIC40 provided a negative control to confirm the specificity of the detected interactions. **d**, BiFC analysis of the interactions between PUX10 and major TOC protein isoforms. Protoplasts transiently co-expressing PUX10 and either TOC159 or TOC33 fused to nYFP and cYFP fragments of YFP, respectively, were analysed by confocal microscopy. In parallel, CDKA1 was used as a cytosol-localized negative control instead of the TOC components. Representative protoplasts are shown as in **a**. Exposure times and gain settings were identical. Scale bar, 10 µm. Source data

First, BiFC assays were used to test for interactions between PUX10 and SP1. Using the BiFC system previously described, full-length PUX10 was fused with cYFP, and SP1 and the negative control proteins sensitive to freezing 2 (SFR2) and cyclin-dependent kinase A1 (CDKA1) were fused with nYFP. Construct pairs encoding complementary nYFP and cYFP fragments were transiently co-expressed in *Arabidopsis* protoplasts and any reconstituted YFP signals were visualized by confocal microscopy (Fig. 5a). In this way, PUX10 was found to interact with SP1 at the chloroplast envelope membrane, whereas neither of the controls (the chloroplast membrane protein SFR2 nor the cytosolic protein CDKA1) showed appreciable interaction with PUX10.

To corroborate the results from the BiFC experiments and validate the interaction between PUX10 and SP1, co-IP experiments were performed. The construct encoding PUX10–YFP was transiently expressed in protoplasts, either in combination with a construct encoding MYC-tagged SP1 (SP1–MYC) or alone. In parallel, a YFP–HA construct was co-expressed with SP1–MYC in protoplasts to serve as a further negative control. The SP1–MYC and YFP–HA constructs were previously described^10^. Here SP1–MYC served as the bait and was immunoprecipitated with anti-MYC beads after solubilization of the transfected cells, with PUX10–YFP and YFP–HA serving as prey (Fig. 5b). As expected, only PUX10–YFP (not YFP–HA) was found to co-precipitate with SP1–MYC, indicating a specific interaction between PUX10 and SP1.

BiFC was judged to be an unsuitable method for analysing potential interactions with SP2 given its multi-membrane-spanning structure. Therefore, co-IP analysis was used to assess the interaction between PUX10 and SP2. Constructs encoding PUX10–YFP and MYC-tagged SP2 (SP2–MYC) were transiently co-expressed in protoplasts. In parallel, the two constructs were also singly transfected into protoplasts in control experiments. The SP2–MYC construct was previously described^10^. In this analysis, PUX10–YFP acted as the bait and was immunoprecipitated with anti-GFP beads after solubilization of the cells, with SP2–MYC acting as the prey (Fig. 5c). The results clearly showed a strong, specific interaction between PUX10 and SP2.

### PUX10 interacts with TOC proteins

On the basis of the above-described results, we hypothesized that PUX10 participates in the regulation of the TOC apparatus in a similar fashion to SP1 and SP2. Therefore, the co-IP samples generated above (Fig. 5c) were further analysed using antibodies to TOC and TIC components in additional immunoblotting experiments. The results showed that all three TOC proteins (TOC159, TOC75 and TOC33) had co-precipitated with PUX10, whereas no association was detected for TIC40 (Fig. 5c). This indicated specific interactions between PUX10 and the TOC complex.

**Extended Data Fig. 7:**
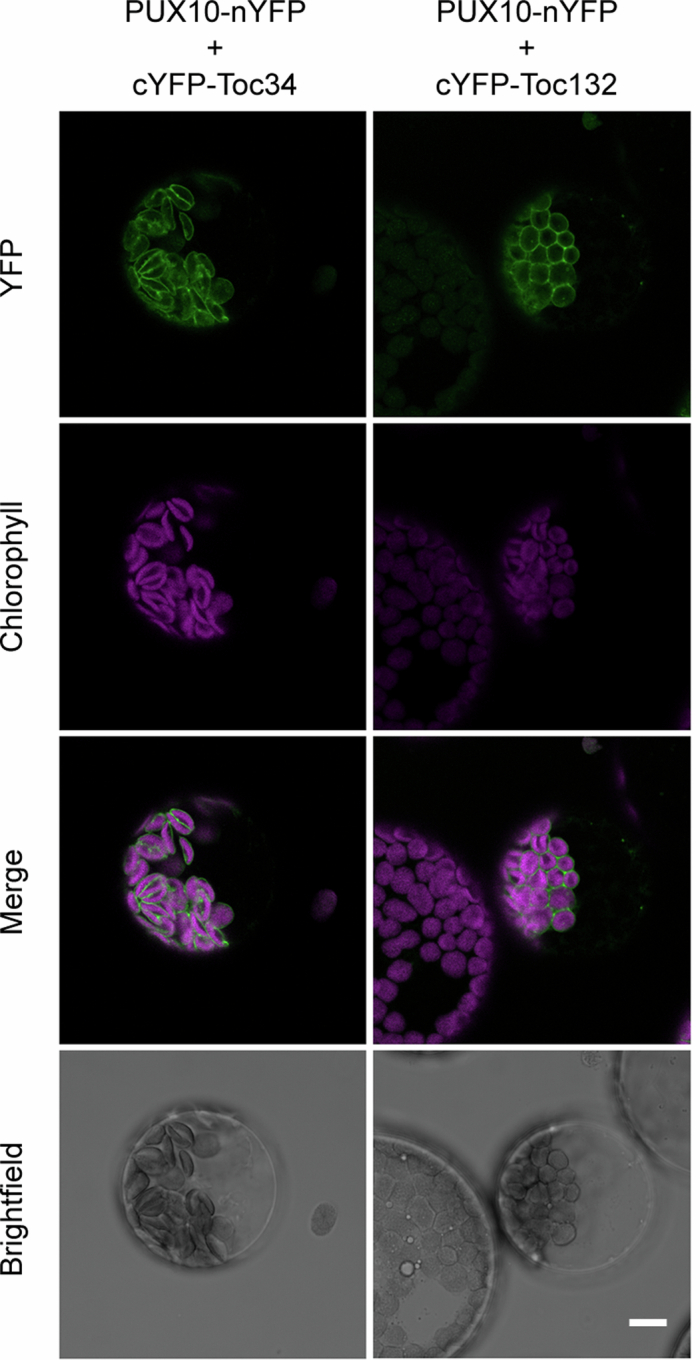
BiFC analysis of the interactions between PUX10 and minor TOC protein isoforms. Protoplasts transiently co-expressing PUX10 and either Toc132 or Toc34 fused to nYFP and cYFP fragments of YFP protein, respectively, were analysed by confocal microscopy. Representative protoplasts are presented. Exposure times and gain settings were identical. Scale bar = 10 µm.

To complement these results with spatial information, the interactions between PUX10 and TOC proteins were also assessed in BiFC experiments. In this case, PUX10 was fused to the nYFP fragment, and TOC159, TOC33 and the negative control protein CDKA1 were all fused to the cYFP fragment. Complementary construct pairs were transiently expressed in protoplasts and any resulting YFP signals were visualized by confocal microscopy (Fig. 5d). As expected, PUX10 was found to interact with both TOC159 and TOC33 at the chloroplast envelope. However, no appreciable interaction was observed for the negative control protein CDKA1, indicating that the detected PUX10–TOC interactions were specific. Moreover, similar BiFC analyses indicated that TOC132 and TOC34 (which are minor isoforms of TOC159 and TOC33, respectively, in *Arabidopsis*) also interact with PUX10 at the chloroplast envelope (Extended Data Fig. 7).

**Fig. 6:**
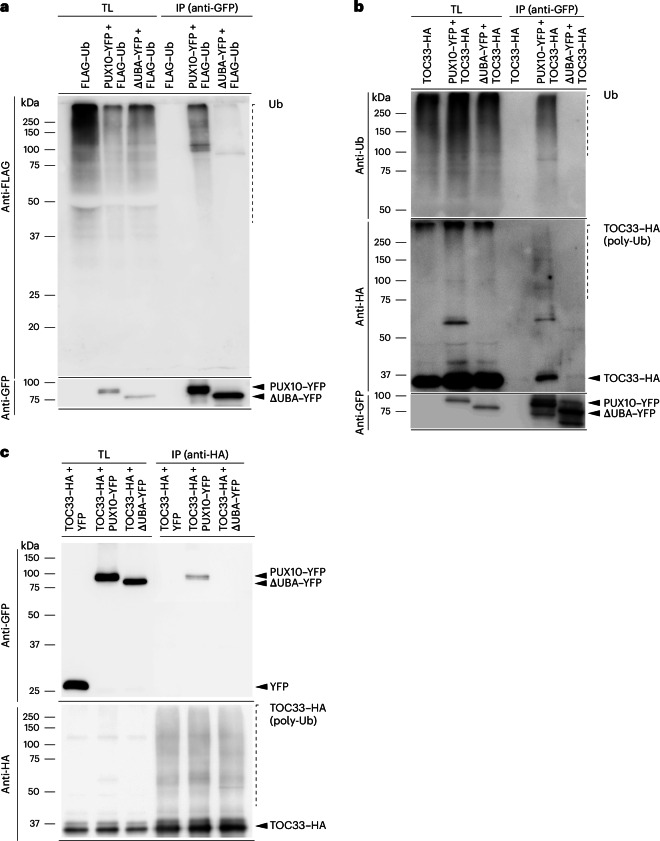
PUX10 interaction with ubiquitin and ubiquitinated TOC33 via its UBA domain. **a**, Analysis of the interaction of PUX10 with ubiquitin. Protoplasts transiently expressing the indicated proteins were solubilized and subjected to anti-GFP co-IP analysis. Anti-GFP immunoblot analysis verified the enrichment of the PUX10–YFP or ΔUBA–YFP proteins, and anti-FLAG analysis assessed co-purification of FLAG-tagged (poly)ubiquitin. **b**, Analysis of the interaction of PUX10 with ubiquitinated TOC33. Protoplasts expressing the indicated proteins were solubilized and subjected to anti-GFP co-IP analysis. Anti-GFP immunoblot analysis verified the enrichment of the PUX10–YFP or ΔUBA–YFP proteins, and anti-HA analysis assessed co-purification of TOC33–HA; both unmodified (see arrowhead) and high-molecular-weight modified forms of TOC33–HA were detected. Parallel analysis of the samples by anti-ubiquitin immunoblotting provided evidence that the high-molecular-weight species were polyubiquitinated TOC33. **c**, Analysis of the interaction of ubiquitinated TOC33 with WT PUX10 or PUX10 lacking its UBA domain. Protoplasts co-expressing the indicated proteins were solubilized and subjected to anti-HA co-IP analysis. Anti-HA immunoblot analysis verified the enrichment of the TOC33, and anti-GFP analysis assessed co-purification of the PUX10–YFP or ΔUBA–YFP proteins. Both unmodified (see arrowhead) and high-molecular-weight modified forms of TOC33–HA were enriched. Dashed verticle lines indicate ubiquitinated proteins. poly-Ub, polyubiquitin; Ub, ubiquitin. Source data

As noted earlier, PUX10 possesses an N-terminal UBA domain, which is a well-known ubiquitin-binding module. To investigate whether PUX10 indeed has the capacity to bind ubiquitin, co-IP analysis was performed. Constructs encoding either full-length PUX10 or the ΔUBA variant fused to YFP (that is, PUX10–YFP or ΔUBA–YFP) were co-expressed in protoplasts along with a previously described construct encoding FLAG-tagged ubiquitin (FLAG–Ub)^9^. Here the YFP fusions acted as bait and were immunoprecipitated using anti-GFP beads after solubilization, with FLAG–Ub acting as the prey (Fig. 6a). The results showed a strong interaction between full-length PUX10 and ubiquitin (in fact, polyubiquitin smears), and this interaction was dependent on the UBA domain. Given that TOC proteins are ubiquitinated by SP1 before being targeted to the cytosolic 26S proteasome for degradation, it was hypothesized that the UBA domain of PUX10 is important for the interaction between PUX10 and ubiquitinated TOC proteins. Thus, the co-IP analysis above was repeated using HA-tagged TOC33 (TOC33–HA)^10^ in place of FLAG-Ub (Fig. 6b). The results clearly indicated that PUX10 is capable of interacting with polyubiquitinated TOC33 through its UBA domain. The fact that unmodified TOC33–HA was similarly precipitated most likely reflects the fact that ubiquitinated and unmodified proteins are present together in complexes. To corroborate the interaction between PUX10 and ubiquitinated TOC proteins, a reciprocal assay using the same constructs was performed. In this assay, the TOC33–HA acted as bait and was immunoprecipitated using anti-HA beads after solubilization, with PUX10–YFP and ∆UBA–YFP acting as the prey (Fig. 6c). The results confirmed the interaction between PUX10 and (ubiquitinated) TOC33, and that this interaction is dependent on the UBA domain.

Collectively, these results from different BiFC and co-IP experiments showed a clear association between PUX10 and the components and substrates of CHLORAD at the chloroplast OEM.

### The *pux10* mutation suppresses the *ppi1* phenotype

**Fig. 7:**
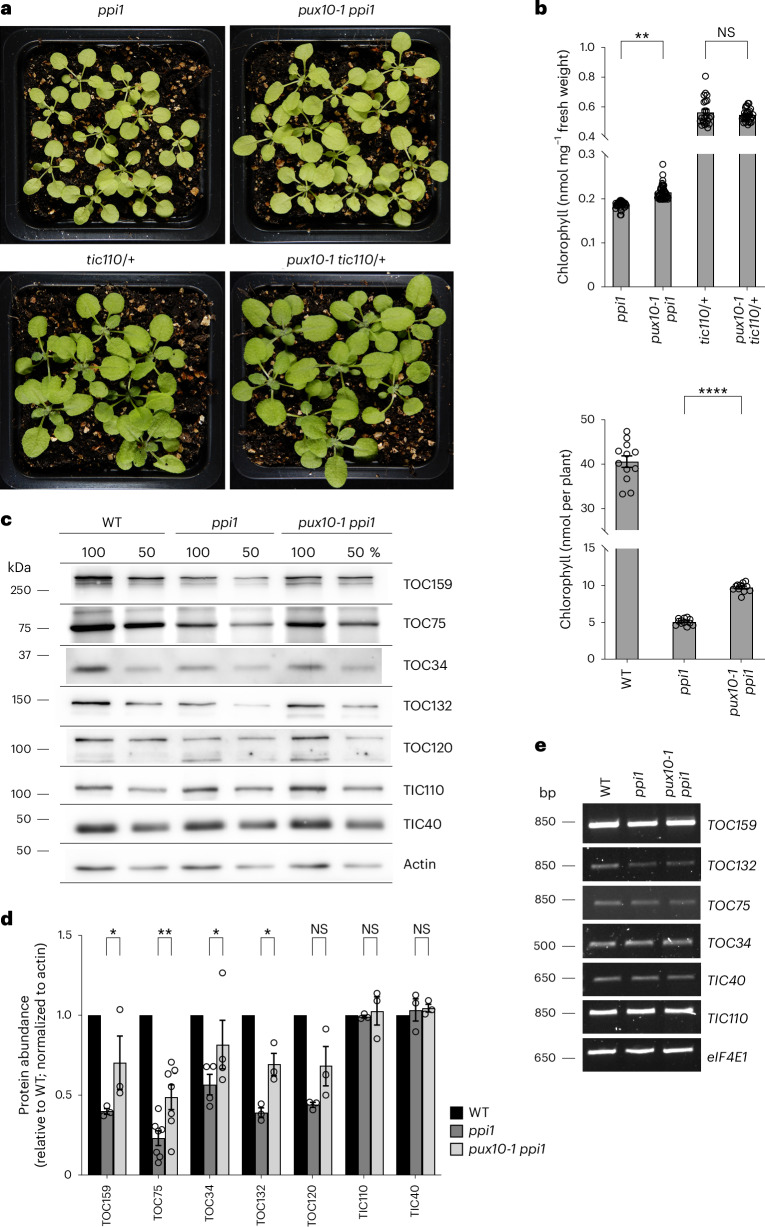
The *pux10* mutation suppresses the *ppi1* phenotype. **a**, Phenotypes of 3-week-old *pux10-1 ppi1* and control seedlings grown on soil. Controls included the *pux10-1 tic110*/*+* double mutant; the *tic110* genotype was analysed in the heterozygous state as the homozygous state is lethal^43^. Representative plant images are shown. Identical camera settings were used and all images are at the same magnification. **b**, Quantification of chlorophyll concentration in *pux10-1 ppi1* double-mutant and control plants. Measurements were taken on the day of photography in **a**. First, the leaves were analysed using a Konica Minolta SPAD-502 meter (top). The values shown are means ± s.e.m. from 30 leaves per genotype. Second, chlorophyll in the aerial tissues of the plants was extracted and quantified using a spectrophotometer (bottom). The values shown are means ± s.e.m. from five plants per genotype. Asterisks indicate significance according to an unpaired two-tailed Student’s *t*-test. ^**^P < 0.005, ^****^P < 0.0001. **c**,**d**, Immunoblot analysis of TOC and TIC protein levels in WT, *ppi1* and *pux10-1 ppi1* plants. **c**, four-week-old plants of indicated genotypes were subjected to immunoblotting analysis. Two different loadings of each sample (100% and 50%) were analysed. Actin was used as a loading control. Representative blot images are shown. **d**, quantification of the immunoblot data presented in **c**, and of other similar datasets, was performed. Band intensities were quantified and normalized to corresponding actin data; the data are presented as ratios relative to the WT value for each protein. Data were derived from multiple technical replicates and were representative of three biological replicates. Sample size (*n*) for each protein was as follows: TOC159 (3), TOC75 (7), TOC34 (4), TOC132 (3), TOC120 (3), TIC110 (3) and TIC40 (3). The values shown are means ± s.e.m. Asterisks indicate significance according to an unpaired two-tailed Student’s *t*-test. ^*^P < 0.05, ^**^P < 0.005. **e**, Analysis of mRNA expression of important translocon component genes in WT, *ppi1* and *pux10-1 ppi1* plants. Total RNA isolated from 20-day-old plants was analysed by RT-PCR for the indicated genes and the reference gene *eIF4E1*. Amplifications used a limited number of cycles to avoid saturation. NS, not significant. Source data

The interaction of PUX10 with TOC proteins suggested a functional link between PUX10 and the TOC apparatus. To investigate this possibility, the *pux10-1* mutation was introduced into the *ppi1* single-mutant background^14^ and, as a control, the *tic110*/+ mutant background^43^. The resulting *pux10-1 ppi1* double-mutant plants showed a moderate increase in chlorophyll content and leaf size relative to single-mutant *ppi1* control plants (Fig. 7a,b). In contrast, *pux10-1 tic110*/+ double-mutant control plants showed no phenotypic differences from *tic110*/*+* single-mutant plants, which show mild chlorosis, indicating that the effect on *ppi1* was specific; note that the *tic110* genotype was analysed in the heterozygous state as the homozygous state is lethal^43^.

To investigate the basis for this *ppi1* suppression effect, total protein extracts were prepared from WT, *ppi1* and *pux10-1 ppi1* plants and the abundance of TOC proteins in the samples was analysed by immunoblotting. Notably, all tested TOC proteins showed substantially recovered levels in the *pux10-1 ppi1* double-mutant plants, relative to *ppi1*, whereas the IEM proteins TIC40 and TIC110 were largely unaffected by the *pux10* mutation (Fig. 7c,d). These changes in TOC protein abundance were not attributable to pretranslational events because *TOC* transcript levels were comparable in the different genotypes (Fig. 7e). Thus, the *ppi1* suppression mediated by *pux10*, much like that mediated by *sp1* and *sp2* as previously described^9,10^, is linked to partially restored TOC protein accumulation.

### The *pux10* mutation abrogates effects of *SP1* overexpression

Having identified the interaction between PUX10 and SP1, we wished to investigate a possible functional link between the two components. It is well known that the *sp1* mutation suppresses the *ppi1* phenotype to produce bigger and greener plants that have increased abundance of TOC proteins and chlorophyll and improved chloroplast ultrastructural organization^9^. Conversely, SP1 overexpression (SP1-OX) greatly enhances the chlorosis of *ppi1* by increasing the ubiquitination of the residual TOC proteins to prime their proteasomal degradation, leading to severe depletion of TOC proteins, reduced chlorophyll concentration, and smaller and paler plants^9^. Given that Cdc48 is important for the degradation of ubiquitinated TOC proteins^10^ and that PUX10 recruits Cdc48 to the chloroplast OEM (as shown earlier), we hypothesized that PUX10 is required for SP1 function and therefore for the excessive TOC protein removal seen upon *SP1* overexpression. If this is indeed the case, then the *pux10* mutation should block or reduce the severe phenotypic effects of SP1-OX in *ppi1* plants.

**Fig. 8:**
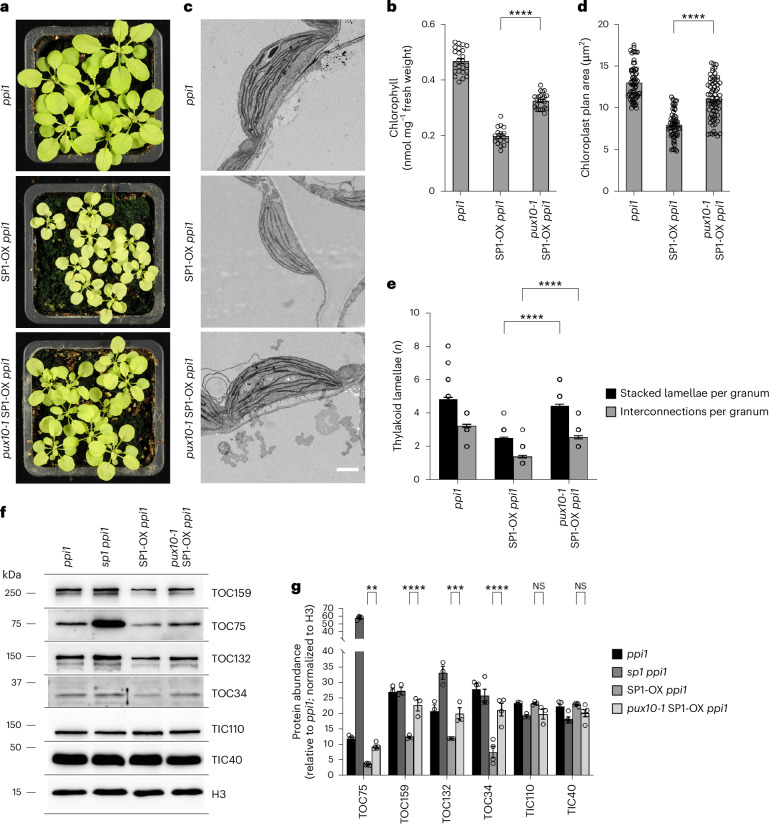
The *pux10* mutation abrogates the effects of SP1 overexpression. **a**, Phenotypes of 4-week-old *pux10-1* SP1-OX *ppi1* triple-homozygous and control seedlings grown on soil. Representative plant images are shown. Identical camera settings were used and all images are at the same magnification. **b**, Quantification of chlorophyll concentration in the plants. Measurements were taken on the day of photography in **a** using a Konica Minolta SPAD-502 meter. The values shown are means ± s.e.m. from 30 leaves. Asterisks indicate significance according to an unpaired two-tailed Student’s *t*-test. ^****^P < 0.0001. **c**–**e**, Ultrastructure analysis of rosette leaf chloroplasts in *pux10-1* SP1-OX *ppi1* and control plants. **c**, samples from 4-week-old plants were analysed by TEM and typical organelles are shown. Scale bar, 1 µm. The presented electron micrographs, and other similar micrographs, were used to determine chloroplast cross-sectional area (**d**) and thylakoid development (**e**), which includes the number of lamellae per granal stack and the number of membrane interconnections per granal stack. The values shown are means ± s.e.m. from 60 chloroplasts per genotype in **d** and 50–80 chloroplasts per genotype in **e**. Asterisks indicate significance according to an unpaired two-tailed Student’s *t*-test. ^****^P < 0.0001. **f**,**g**, Immunoblot analysis of TOC and TIC protein levels in *pux10-1* SP1-OX *ppi1* and control plants. **f**, four-week-old plants of the indicated genotypes were subjected to immunoblotting analysis. Equal amounts of the different samples were loaded. Histone H3 was used as a loading control. Representative blot images are shown. **g**, quantification of the immunoblot data presented in **f**, and of other similar datasets, was performed. Band intensities were quantified and normalized to corresponding H3 data; the data are presented as ratios relative to the *ppi1* value for each protein in arbitrary units. Data were derived from multiple technical replicates and were representative of three biological replicates. Sample size (*n*) for each protein was as follows: TOC75 (5), TOC159 (3), TOC132 (3), TOC34 (4), TIC110 (3) and TIC40 (5). The values shown are means ± s.e.m. Asterisks indicate significance according to an unpaired two-tailed Student’s *t*-test. ^**^P = 0.0016, ^***^P = 0.0006, ^****^P < 0.0001. NS, not significant. Source data

**Extended Data Fig. 8:**
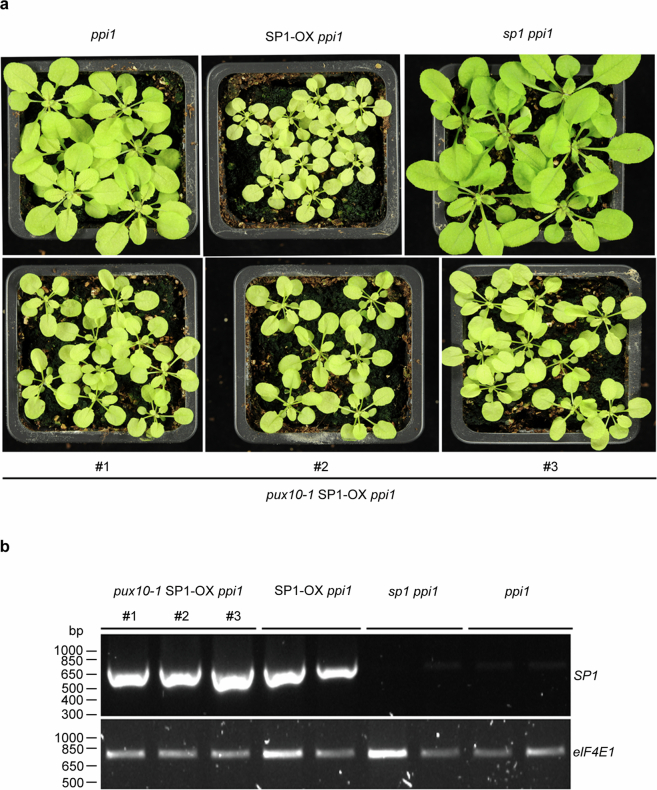
Identification and preliminary analysis of *pux10-1* SP1-OX *ppi1* plants. **a**, Phenotype screening for 4-week-old *pux10-1* SP1-OX *ppi1* plants grown on soil. The *pux10-1* mutant was crossed to an SP1-OX *ppi1* transgenic line, and pale F_2_ individuals showing a greener phenotype than SP1-OX *ppi1* controls were identified and carried forward for genotyping and further analysis. Three representative *pux10-1* SP1-OX *ppi1* F_3_ lines and controls plants are presented. Identical camera settings were employed, and all images are at the same magnification. **b**, Analysis by RT-PCR of the overexpression of *SP1* in the selected F_3_ lines. Total RNA samples isolated from 2-week-old plants of the F_3_ lines shown in **a**, and from appropriate controls, were analysed by RT-PCR for *SP1* and the reference gene *eIF4E1*. The SP1-OX *ppi1* line acted as a positive control, while *ppi1* and *sp1 ppi1* acted as negative controls (the native *SP1* mRNA was below the level of detection in *ppi1*). Amplifications employed a limited number of cycles to avoid saturation, and products were analysed by agarose gel electrophoresis. Source data

To test this hypothesis, the *pux10-1* mutation was crossed into the SP1-OX *ppi1* background, and triple homozygous *pux10-1* SP1-OX *ppi1* plants were identified via phenotyping, genotyping and RT-PCR analysis (Extended Data Fig. 8). As expected, SP1-OX *ppi1* plants were much smaller and more chlorotic than *ppi1* plants. Most interestingly, and in accordance with the above hypothesis, the *pux10-1* SP1-OX *ppi1* plants were substantially greener and larger than SP1-OX *ppi1* plants, although they were not completely recovered to the level of *ppi1* single-mutant plants (Fig. 8a). Chlorophyll concentration was significantly increased in *pux10-1* SP1-OX *ppi1* plants relative to SP1-OX *ppi1* plants, although still considerably less than in *ppi1* (Fig. 8b); this was consistent with the visible difference between the *pux10-1* SP1-OX *ppi1* and *ppi1* plants.

To determine whether the suppression of chlorosis observed in *pux10-1* SP1-OX *ppi1* plants was linked to effects on chloroplast biogenesis, the organelles were analysed by transmission electron microscopy (TEM). This analysis showed that *pux10-1* SP1-OX *ppi1* plants possess chloroplasts of increased size and improved ultrastructure compared with SP1-OX *ppi1* plants (Fig. 8c). Quantitative analysis showed a clear increase in chloroplast cross-sectional area in *pux10-1* SP1-OX *ppi1* relative to SP1-OX *ppi1* (Fig. 8d), and there were increased numbers of thylakoid lamellae per granal stack and interconnections between granal stacks (Fig. 8e). Nonetheless, the overall development of chloroplasts in *pux10-1* SP1-OX *ppi1* was still less than that in *ppi1*, which was consistent with the visible differences between the plants.

In SP1-OX *ppi1* plants, the severe phenotype is related to reduced abundance of TOC proteins^9^. To test whether the suppression effect observed in *pux10-1* SP1-OX *ppi1* plants is linked to restored TOC protein abundance, total protein extracts from the plants were analysed by immunoblotting. This analysis revealed substantial increases in TOC protein abundance in *pux10-1* SP1-OX *ppi1* plants relative to SP1-OX *ppi1* plants (Fig. 8f,g). However, in line with previous observations concerning the *sp1 ppi1* mutant^9^, there were no comparable differences in the levels of TIC components, indicating that the effects of PUX10, similarly to those of SP1, are specific to TOC components.

**Extended Data Fig. 9:**
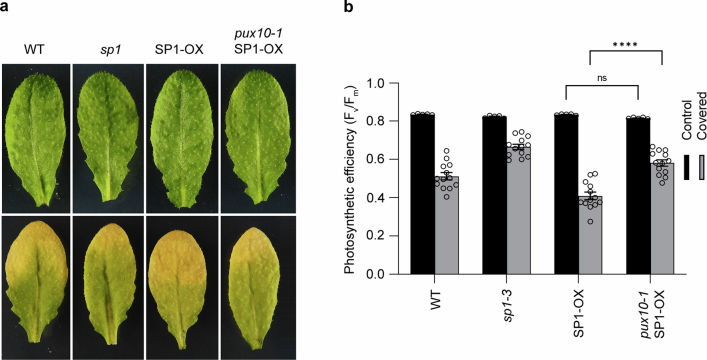
Evaluation of the effect of PUX10 on SP1-dependent dark-induced leaf senescence. **a**, Visual assessment of leaf senescence. Attached leaves of 4-week-old plants of the indicated genotypes were left uncovered (top; control) or were covered with aluminium foil (bottom; covered), and the plants were kept under standard growth conditions for 5 days before leaf detachment and photography. A representative leaf from each genotype for each condition is shown. Identical camera settings were employed, and all images are at the same magnification. **b**, Quantitative assessment of photosynthesis. The maximum photochemical efficiency of photosystem II (*F*_v_/*F*_m_) was measured for leaves similar to those presented in **a** using a CF Imager, to estimate the extent of senescence. The data were derived from eight leaves per genotype (for control, uncovered), and ten leaves per genotype (for senescence, covered). Values shown are means ± s.e.m. The asterisks indicate significance according to an unpaired two-tailed Student’s *t*-test (****P < 0.0001; ns, not significant).

To further explore the physiological significance of PUX10, we conducted an analysis of leaf senescence. The expression level of *SP1* was previously shown to influence leaf senescence and other developmental processes in which plastids (the organelle family to which chloroplasts belong) change type^9,12^. High levels of SP1 activity promote the reorganization of the TOC machinery so that it is better able to bring about the organellar proteome changes that underly such transitions, and in the case of aging or dark-treated leaves, this results in accelerated leaf senescence (due to accelerated conversion of chloroplasts into gerontoplasts)^9,12^. To determine whether PUX10 also plays a role in such developmental processes, the *pux10-1* mutation was crossed into the SP1-OX background and the resulting *pux10-1* SP1-OX plants were subjected to leaf-senescence analysis, along with WT, *sp1* and SP1-OX control plants (Extended Data Fig. 9).

In line with previously published results, the *sp1* mutant showed retarded leaf senescence, as judged by reduced visible yellowing and a smaller decline in the maximal photochemical efficiency of photosystem II (variable fluorescence (*F*_v_)/maximum fluorescence (*F*_m_)) relative to WT, whereas SP1-OX leaves showed accelerated senescence. Interestingly, the *pux10-1* SP1-OX leaves showed a significant reduction in senescence compared with SP1-OX leaves based on both visual assessment of the material and measurements of the *F*_v_/*F*_m_ parameter (Extended Data Fig. 9). Thus, these data show the physiological importance of PUX10 during an important developmental transition and further support the conclusion that PUX10 cooperates with SP1 in the CHLORAD pathway.

## Discussion

Thousands of nucleus-encoded proteins are imported into chloroplasts via the TOC–TIC apparatus in the chloroplast envelope membranes^1–8^. Hence, protein import is vital for chloroplast biogenesis and operation and for plant growth and development. The CHLORAD system regulates the turnover of TOC proteins to manipulate protein import and thereby optimizes the organellar proteome and functions^8^. Three key components of CHLORAD have been identified (SP1, SP2 and Cdc48), which work cooperatively in the ubiquitination, retrotranslocation and degradation of TOC proteins. The Cdc48 ATPase drives the extraction of target proteins through the SP2 channel, following their ubiquitination by SP1; but how Cdc48 is recruited to the chloroplast surface to enable such action was previously unknown.

Previous studies identified PUX proteins as being involved in regulating various activities of Cdc48 in plants^26–30^ but their potential involvement in CHLORAD was unexplored. The PUX10 protein was recently identified as an LD-localized Cdc48 adaptor protein that regulates the degradation of LD proteins during embryogenesis and seed germination^31,32^. Intriguingly, the subcellular localization of PUX10 swiftly changed from LDs to chloroplasts during seed maturation^31^. Given its relevance to the interpretation of our study, we further analysed the subcellular localization of PUX10. During embryogenesis, PUX10 was predominantly localized in LDs at the bent cotyledon stage; however, when embryos reached the mature green stage, it was predominantly localized in chloroplasts and a characteristic ring-shaped distribution around the chloroplast envelope was detected (Supplementary Fig. 1). During germination, PUX10 was found to be associated with LDs at the earliest stages (6 h of germination; although it should be noted that plastid localization at this stage could not be ruled out owing to the difficulty in detecting these organelles due to the lack of chlorophyll) but then later PUX10 was detected around chloroplasts (24 h of germination) before finally becoming clearly and predominantly located at the surface of the chloroplasts (after 48 h of germination) (Supplementary Fig. 1). Thus, it appears that PUX10 has multifunctional roles that are delivered in a phased manner during plant development.

In leaves, we found PUX10 to be distinctly and predominantly associated with the chloroplasts, where it was clearly localized in the envelope (Fig. 1 and Extended Data Figs. 1 and 2). Nonetheless, colocalization analysis indicated that a minor fraction of PUX10 is associated with the ER (Supplementary Fig. 2), as was also described in previous studies^31,32^. Interestingly, such partitioning between organelles was also observed for Ubx2 and FAS-associated factor 2 (FAF2, also known as Ubxd8), which are homologues of PUX10 in yeast and mammals, respectively^33,44–48^. The Ubx2 protein is best known for its role in recruiting Cdc48 to the ER membrane in ERAD but it was also shown to perform a similar role in mitochondria^47^. Therefore, multilocation operation may be a conserved feature of this group of proteins. It will be interesting to investigate in future work whether PUX10 indeed participates in ER-associated protein quality control processes, in addition to CHLORAD.

To elucidate the function of PUX10 in chloroplasts, a key step was to establish the topology of the protein. Our analysis showed that PUX10 is anchored in the chloroplast OEM by two TM spans such that both termini face the cytosol (Figs. 1 and 2). Thus, the UBA and UBX domains both have cytosolic orientation, enabling interactions of PUX10 with different sets of functional partners. Our results indicated that PUX10 interacts with Cdc48 via its UBX domain, enabling it to recruit Cdc48 to the chloroplast envelope (Figs. 3 and 4), and with ubiquitinated TOC proteins via its UBA domain (Figs. 5 and 6). Thus, PUX10 effectively acts as a bridge that brings together cytosolic Cdc48 and ubiquitinated TOC proteins in the OEM to promote the retrotranslocation step of CHLORAD. Indeed, PUX10 was also shown to interact with SP1 and SP2 (Fig. 5), supporting the notion that it is a key component of the CHLORAD system acting in the regulated turnover of the TOC machinery.

This view was confirmed through genetic analysis. Apart from effects on LD size during embryogenesis^31,32^ and seed germination (Extended Data Fig. 3d,e), *Arabidopsis pux10* single mutants showed no obvious phenotypic differences from WT plants (Extended Data Figs. 3 and 4), much like *sp1* and *sp2* mutants^9,10^. However, the *pux10* mutation did suppress the pale phenotype of *ppi1*. Importantly, this suppression was linked to increased TOC protein abundance (Fig. 7), paralleling closely the *ppi1* suppression mediated by the *sp1* and *sp2* mutations^9,10^. This provided strong evidence that PUX10 is involved in chloroplast functions in leaves and most likely in the regulation of TOC activity by CHLORAD.

As a core component of the CHLORAD system, the E3 ubiquitin ligase SP1 labels TOC proteins with ubiquitin for degradation by the cytosolic 26S proteasome. Thus, when it is overexpressed in an already TOC-compromised background (*ppi1*), severe chlorosis results^10^. Intriguingly, when the *pux10* mutation was introduced into the SP1-OX *ppi1* background, the resulting plants were much larger and greener than SP1-OX *ppi1* plants, and presented increased abundancies of chlorophyll and TOC proteins and improved chloroplast ultrastructural organization (Fig. 8). This provided a clear demonstration that PUX10 is required for SP1 function in vivo. Previous work showed that SP1 plays a crucial role in plant developmental transitions in which plastids change type, such as leaf senescence^9,12^. We found that the stimulating effect of *SP1* overexpression on leaf senescence^9^ was attenuated by the *pux10* mutation (Extended Data Fig. 9), providing further strong evidence that PUX10 and SP1 act in the same pathway (that is, CHLORAD) and of the physiological importance of PUX10.

It is noteworthy that the phenotypic suppression of *ppi1* delivered by *pux10* (Fig. 8) was not as strong as that delivered by the *sp1* or *sp2* mutations^9,10^, and that the *pux10* mutation only partially blocked the phenotypic consequences of SP1 overexpression. These observations suggest that there may be functional redundancy between PUX10 and as-yet-unknown proteins, such as other members of the PUX family^49^ or other Cdc48 adaptor proteins^19,20^. Although our comprehensive analysis of PUX protein localization did not identify any other family members with distinct chloroplast localization (Extended Data Fig. 1), we cannot completely rule out the possibility that additional PUX proteins function at the chloroplast surface. It is also possible that PUX10 is only strictly required in specific situations—more so than either SP1 or SP2, which are clearly responsible for core functions of the CHLORAD system (that is, substrate ubiquitination and conductance, respectively). Regardless, it will be interesting to explore in future studies what other factors are involved in the ubiquitin-mediated degradation of TOC proteins.

**Extended Data Fig. 10:**
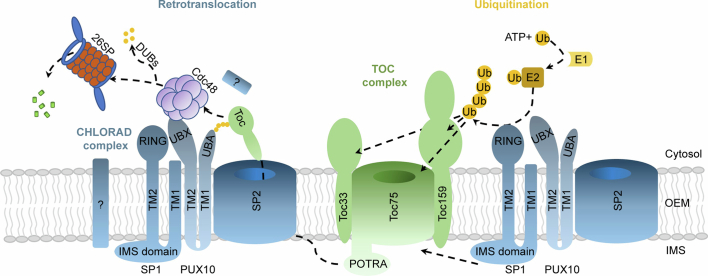
A model for the role of PUX10 in the CHLORAD pathway. In CHLORAD, TOC proteins are marked with polyubiquitin through the action of E1, E2 and SP1 E3 ligase enzymes. The PUX10 protein is integrated in the chloroplast OEM via its transmembrane domains (TMs), and it acts to recruit Cdc48 from the cytosol to the chloroplast surface. Moreover, PUX10 acts as a link or bridge between the ubiquitinated TOC proteins and Cdc48, through its cytosol-oriented UBA and UBX domains respectively. Thus, PUX10 facilitates the retrotranslocation of ubiquitinated TOC proteins from the chloroplast OEM. Upon dislocation into the cytosol, ubiquitin is cleaved from TOC proteins by yet unknown deubiquitinase (DUB) enzymes and the TOC proteins are degraded by the 26S proteasome. *Arabidopsis* TOC proteins are shown and labelled by convention according to their molecular masses in kilodaltons. Putative unknown proteins (?) that share functional redundancy with PUX10 are shown. Abbreviations: CHLORAD, chloroplast-associated protein degradation; Cdc48, cell division cycle protein 48; DUBs, deubiquitinases; IMS, intermembrane space; OEM, outer envelope membrane; POTRA, polypeptide transport-associated domain; RING, really interesting new gene; SP1, suppressor of *ppi1* locus 1; SP2, suppressor of *ppi1* locus 2; TM, transmembrane domain; Ub, ubiquitin; UBA, ubiquitin-associated; UBX, ubiquitin regulatory X; 26SP, 26S proteasome.

Collectively, our work has unveiled PUX10 as a chloroplast-bound adaptor protein that recruits Cdc48 to the chloroplast surface, promoting its interaction with ubiquitinated TOC proteins so that they may be extracted into cytosol for degradation by the 26S proteasome. In view of its physical and functional association with the established CHLORAD machinery, we conclude that PUX10 is a bona fide component of the CHLORAD system (Extended Data Fig. 10).

## Methods

### Plant materials and growth conditions

All *Arabidopsis thaliana* plants used in this work were of the Columbia-0 (Col-0) ecotype. Two T-DNA mutant lines, *pux10-1* (SAIL_1187_B06) and *pux10-4* (WiscDsLox424B8), were obtained from the Salk Institute Genomic Analysis Laboratory (SIGnAL)^50^ via the Nottingham Arabidopsis Stock Centre and confirmed by genomic PCR and RT-PCR, as previously described^51^. The *pux10-1* mutant has been previously described^31,32^ but the *pux10-4* mutant has not previously been studied. Two further alleles of *pux10*, namely *pux10-2* and *pux10-3*, have been described by other research groups^31,32^ but were not used in this study. The *ppi1, tic110/+*, *sp1* and *sp1 ppi1* mutants and the SP1-OX and SP1-OX *ppi1* transgenic lines have been previously described^9,14,43^. The ER, mitochondria and Golgi marker lines were provided by Dr Niloufer G. Irani and the late Dr Ian Moore of Oxford University^52–54^.

For most experiments, plants were grown on soil: 80% (v/v) compost (modular seed; Sinclair) and 20% (v/v) vermiculite (fine particle size; Sinclair Pro). For in vitro growth, seeds were surface sterilized, sown on Murashige–Skoog (MS) agar medium in petri plates, cold treated at 4 °C and kept in a growth chamber (Percival Scientific) thereafter, as previously described^55^. All plants were grown under a long-day cycle (16 h light and 8 h dark, 100–120 µE m^−2^ s^−1^) at 20 °C with ~60% relative humidity. For the induction of CDC48-WT or CDC48-DN expression in the corresponding transgenic lines, 8-day-old seedlings were transferred to liquid MS medium supplemented with 4 μM oestradiol (Sigma) and incubated for an additional 2 days as previously described^10^.

For germination assays, seeds were surface sterilized, sown on MS agar medium in petri plates, cold treated at 4 °C and kept in a growth chamber (Percival Scientific) thereafter at 20 °C under continuous light.

### Physiological studies

Chlorophyll measurement was performed as previously described by using a Konica Minolta SPAD-502 meter for the analysis of rosette-stage plants^56^, or following *N*,*N*′-dimethylformamide extraction using a spectrophotometer for the analysis of seedlings^14,57–59^.

Dark treatments for the induction of leaf senescence were conducted as previously described^9,60^. Developmentally equivalent leaves of 28-day-old plants were wrapped in aluminium foil while still attached to the plant and then left under standard growth conditions for 5 days. *F*_v_/*F*_m_ was determined by measuring chlorophyll fluorescence using a CF Imager (Technologica) as previously described^9,12^. Three experiments were performed and approximately ten leaves (each one from a different plant) were analysed per genotype in each experiment.

### Plasmid constructs

All primers used are listed in Supplementary Table 1. The SP1–MYC, SP2–MYC, YFP–HA, FLAG–Ub, TOC33–HA, Cdc48–HA and Cdc48–YFP constructs have all been previously described^9,10^. The Cdc48(∆Nterm)–HA construct was generated by using modified 5′ and 3′ primers to amplify from Col-0 cDNA a truncated *Cdc48* coding sequence (CDS) (encoding a polypeptide lacking the first 190 residues), which was then cloned into the pDONR201 entry vector (Invitrogen) and subcloned into the modified p2GW7 vector^61^ with a C-terminal HA tag for protoplast transfection. The new PUX10-related constructs for this study were generated as follows. The *PUX10* CDS was amplified in different forms from Col-0 cDNA by using primers at the 5′ and 3′ termini of the CDS; by using a modified 5′ primer that adds an N-terminal FLAG tag; by using alternative 5′ and/or 3′ primers to generate the ΔUBX, ΔUBA and ΔUBX/UBA variants; and by using modified 5′ and 3′ primers to generate the triple UBX-domain point mutations of PUX10(mut). The *PUX10* CDS lacking the coding region of the TM domains (ΔTM1/2) was generated by overlap-extension PCR. The *PUX10* promoter (*pPUX10*) was amplified from Col-0 genomic DNA by using primers that add 5′ HindIII and 3′ SpeI sites. To generate a modified pK7YWG2 binary vector^61^, the amplified *PUX10* promoter sequence was cloned into pGEM-T Easy (Promega), sequenced, and then subcloned into 5′ HindIII and 3′ SpeI sites of pK7YWG2 to replace the *35S* promoter. The Gateway Cloning System (Invitrogen) and vectors driven by the *35S* promoter (with the exception of *pPUX10*) were used for most constructs, and all donor vectors were verified by DNA sequencing. To generate C-terminal YFP tag fusions, the *PUX10* CDSs (all forms) were cloned into the pDONR201 entry vector (Invitrogen) and then subcloned either into the p2GWY7 vector^61^ for protoplast transfection or into the pK7YWG2 or the modified (*pPUX10*) pK7YWG2 binary vector^61^ for stable plant transformation. The full-length *PUX10* CDS (no tag) was also cloned into a previously described modified binary vector pH2GW7, which provides a C-terminal HA tag for stable plant transformation^9,10^. To generate BiFC constructs, selected CDSs were cloned into the pGEM-T Easy vector, sequenced and then subcloned as follows: into 5′ BglII and 3′ SalI sites of the pSAT4A-nEYFP-N1 and pSAT4A-cEYFP-N1 vectors^37,62^ for PUX10–nYFP and PUX10–cYFP, respectively; into 5′ XholI and 3′ EcoRI sites of the pSAT4A-cEYFP-N1 vector for ΔUBX–cYFP; and into 5′ KpnI and 3′ XmaI sites of the pSAT4A-nEYFP-N1 vector for SFR2–nYFP. The Cdc48–nYFP, SP1–nYFP, CDKA–nYFP, cYFP–TOC33, cYFP–TOC159, cYFP–TOC34 and cYFP–TOC132 constructs have all been previously described^10,63^. Routine sequence analyses for generating plasmid constructs were conducted using DNAStar Lasergene v.7.2.

### Protoplast isolation and transient assays

Protoplast isolation and transient assays were carried out as previously described^64^. When required, bortezomib (Selleckchem) (prepared as a 10 mM stock solution in DMSO) was added to the protoplast culture medium after 15 h of incubation to a final concentration of 5 μM; subsequently, the culture was incubated for a further 2–3 h before analysis. For YFP fluorescence or co-IP assays, 0.1 ml (~10^5^) or 0.6 ml (~10^6^) aliquots of protoplasts were transfected, respectively, with either 5 μg or 30 μg of plasmid DNA. The samples were analysed after 15–18 h.

### Generation of transgenic lines

The *35*:PUX10–YFP (PUX10-OX in Fig. 3a), *35S*:ΔUBX–YFP, *35S*:ΔUBA–YFP, *35S*:ΔUBX/UBA–YFP, *35S*:ΔTM1/2–YFP, *35S*:FLAG–PUX10–YFP, *35S*:PUX10–HA (PUX10-OX in Fig. 3b) and *pPUX10*:PUX10–YFP transgenic plants were generated by *Agrobacterium*-mediated floral dip transformation^65^. Transformants were selected by using MS medium containing either kanamycin (for the pK7YWG2 vector) or hygromycin B (for the modified pH2GW7 vector). At least ten T_2_ lines for each genotype were analysed by confocal microscopy, immunoblotting or RT-PCR. The *35S*:PUX10–HA transgene was introduced into the CDC48-WT background^10^ (and the *pux10-1* mutation was introduced into the CDC48-DN background^10^) (Fig. 3b) by crossing the corresponding genotypes, followed by immunoblotting or semi-quantitative RT-PCR analysis in the F_2_ generation.

### Microscopy

TEM was performed using mature rosette leaves as previously described^59^. Images were taken using an FEI Tecnai T12 TEM from three biological replicates (different leaves from different individual plants) and analysed using Fiji ImageJ-win32 (ref. ^66^). Quantitative analyses (Fig. 8d,e) were based on at least 60 different plastids per genotype and were representative of 3 individuals per genotype. Chloroplast cross-sectional area was estimated as previously described^43,59^ by using the equation *π* × 0.25 × length × width. Numbers of thylakoid lamellae per granal stack and of interconnections between granal stacks were determined as previously described^9,43^ in a total of ~130 resolvable grana across 3 individuals per genotype.

All fluorescence microscopy and BiFC experiments were conducted at least three times with the same results and typical images are presented. For the imaging of YFP, GFP, RFP and chlorophyll fluorescence signals, in most cases (except for Fig. 1c and Extended Data Fig. 2), protoplasts were examined by using a Leica TCS SP5 confocal microscope equipped with a Leica HC Plan Apochromat CS2 63.0× UV water immersion lens as previously described^63,67^. For Fig. 1c and Extended Data Fig. 2, small leaf tissue samples (~0.5 cm × 0.5 cm) were mounted in perfluorodecalin (PFD) before imaging as described above. PFD easily infiltrates leaf tissue to fill the intercellular air spaces of the mesophyll, enabling high-resolution confocal imaging of the mesophyll^68^. For confocal microscopy experiments, typically YFP fluorescence, chlorophyll autofluorescence, merged YFP and chlorophyll fluorescence and bright-field images are presented.

For BiFC assays, plasmid DNA for two constructs (one nYFP fusion and one cYFP fusion) was co-transfected into WT *Arabidopsis* protoplasts. After overnight incubation, reconstituted YFP signals were analysed by confocal imaging. All images were captured using the same settings to enable comparisons.

For the analysis of LDs, embryos and young seedlings were obtained from different siliques of plants grown on soil or from seeds germinated on MS agar medium in petri plates. Embryos and young seedlings were gently squeezed out of the seed coat using jeweller forceps (Sigma-Aldrich) on a microscope slide under a dissection microscope. They were then stained with Nile Red (dissolved to a concentration of 4 mg ml^−1^ in DMSO and then diluted 500-fold in water before use; Sigma-Aldrich) for 30 min on the microscope slide, before a coverslip was applied with gentle pressure to flatten the stained material. Confocal microscopy was performed using a Zeiss LSM 880 Airy Scan. Excitation (ex) and emission (em) parameters for the detection of the different fluorophores were as follows: YFP (ex/em, 514 nm/521–551 nm), Nile Red (ex/em, 561 nm/580–671 nm) and chlorophyll (ex/em, 633 nm/670–700 nm). The embryos and young seedlings, after their dissection from siliques or germinated seeds, respectively, were also imaged using a Zeiss Stemi 508 microscope equipped with a Axiocam 105 colour camera.

The diameter of LDs was measured using Fiji ImageJ-win32 by drawing the diameter using the ‘line’ tool and measuring it using the ‘measure’ function of the software^69^.

### Chloroplast isolation and protein topology analysis

Chloroplasts were isolated from plants grown in vitro for 8–10 days after the induction of the CDC48-WT and CDC48-DN constructs. To induce expression of the transgenes, 8-day-old seedlings were transferred from MS agar medium to MS liquid medium supplemented with 4 μM oestradiol (Sigma) and incubated for an additional 2 days with gentle shaking under standard growth conditions. Chloroplast isolation and alkaline extraction were performed as previously described^70,71^. Protease treatments were performed as previously described with some minor modifications^72^: 500 µg ml^−1^ thermolysin (with or without 1% Triton X-100) or 500 µg ml^−1^ trypsin was used. After the protease treatments, chloroplast pellets were added directly to 2× sodium dodecyl sulphate–polyacrylamide gel electrophoresis (SDS–PAGE) loading buffer (see below) and then analysed by immunoblotting.

### SDS–PAGE, immunoblotting and co-IP

SDS–PAGE and immunoblotting were performed as previously described^43,73^ with minor modifications. When necessary, gels were stained with InstantBlue Protein Stain (Expedeon).

The primary antibodies used were as follows, with dilution factors provided in parentheses. To identify TOC or TIC components, we used anti-atTOC75-III POTRA-domain^51^ (1:1,000), anti-atTOC159 A-domain^74^ (1:5,000), anti-atTOC132 A-domain^9^ (1:1,000); anti-atTOC120 A-domain^51^ (1:1,000), anti-atTOC33 peptide^75^ (1:500), anti-atTOC34 (1:2,000; AS07 238; Agrisera), anti-atTIC110 stromal domain^76,77^ (1:5,000) and anti-atTIC40 stromal domain^51^ (1:100,000). To detect unrelated proteins as loading controls, we used anti-actin (1:3,000; AS132640; Agrisera) and anti-histone H3 (1:1,000; ab1791; Abcam). Other primary antibodies we used were anti-HA tag (1:1,000; H6908; Sigma), anti-c-MYC tag (1:1,000; ab9106; Abcam), anti-GFP (which detects both GFP and YFP; 1:1,000; SAB4301138; Sigma), anti-FLAG tag (1:1,000; F7425; Sigma) and anti-ubiquitin (1:2,000; 662099; Merk)^10^.

The secondary antibody was anti-rabbit IgG conjugated with horseradish peroxidase (1:5,000; 12-348; Sigma). Chemiluminescence was detected using the EZ-ECL Enhanced Chemiluminescence Detection Kit for HRP (Biological Industries, Sartorius) and an ImageQuant LAS-4000 imager (GE Healthcare). Band intensities were quantified using Aida Image Analyzer v.4.27 software (Raytest). Quantification data were obtained from the results of at least three experiments all showing a similar trend. Typical images are shown in all figures.

For co-IP using YFP-tagged proteins, total protein (~500 mg) was extracted from protoplasts in IP buffer (25 mM Tris-HCl, pH 7.5, 150 mM NaCl, 1 mM EDTA and 1% Triton X-100) containing 0.5% plant protease inhibitor cocktail (Sigma) and centrifuged at 20,000*g* for 10 min at 4 °C. The clear lysate was then incubated with 50 μl of GFP-Trap Magnetic Agarose (ChromoTek) for 2 h to overnight at 4 °C with slow rotation. After 6 washes with 500 μl of IP-washing buffer (25 mM Tris-HCl, pH 7.5, 150 mM NaCl, 1 mM EDTA and 0.5% Triton X-100), bound proteins were eluted by boiling in 2× SDS–PAGE loading buffer (50 mM Tris-HCl, pH 6.8, 20% glycerol, 1% SDS and 0.1 M dithiothreitol) for 5 min, and analysed by SDS–PAGE and immunoblotting. A similar procedure was adopted for co-IP using MYC-tagged proteins, except that 50 μl of EZview Red Anti-c-Myc Affinity Gel (Sigma) was used instead of the GFP-Trap Magnetic Agarose.

### Multiple protein sequence alignment

Protein sequences of PUX10 and UBX2 homologues were obtained from a variety of sources including Phytozome^78^ and Uniprot^79^ by using *Arabidopsis* PUX10 as a query in BLASTP. Multiple sequences were aligned by the ClustalW multiple method, using the BioEdit Alignment Editor software package v.7.2.5.

### Protein complex prediction in silico

The 3D structures of complexes formed by the PUX10 + Cdc48, PUX10(∆UBX) + Cdc48, PUX10 + Cdc48(∆Nterm) and PUX10(∆UBX) + Cdc48(∆Nterm) polypeptide pairs were predicted using Alphafold-Multimer (an extension of AlphaFold2 that uses artificial intelligence to predict protein–protein complexes^39^) as previously described^40^. This analysis was performed by Homma Scientific. The 3D structures of the PUX10(UBX) + Cdc48, PUX10 + Cdc48(Nterm) and PUX10(UBX) + Cdc48(Nterm) polypeptide pairs were predicted by AlphaFold2 using UCSF ChimeraX^80^. Both methods produced two intrinsic model accuracy estimates (ipTM and pTM) and we used a combination of these two estimates (0.8 ipTM + 0.2 pTM) as the confidence metric^39,40^.

### Statistics and reproducibility

Statistical calculations (mean, s.e.m. and *t*-test) were performed using GraphPad Prism v.8.3.0 software. The statistical significance of differences between two experimental groups was assessed by using a two-tailed Student’s *t*-test. Differences between two datasets were considered significant at *P* < 0.05.

The protoplast transient expression analyses of protein localization in Figs. 1b and 4a and Extended Data Fig. 1 were repeated three times independently with similar results. The BiFC assays in Fig. 5a,d and Extended Data Fig. 7 were repeated a minimum of three times independently with similar results. The stable transformations for analysing protein localization in Fig. 1c and Extended Data Fig. 2 were conducted once, although multiple independent lines were analysed in each case. The membrane protein topology analysis in Fig. 2a,b was repeated three times independently with similar results. The co-IP assays for analysing protein–protein interactions in Figs. 4d–f, 5b,c and 6 were repeated a minimum of three times independently with similar results. The semi-quantitative RT-PCR analyses of gene expression in Fig. 7e and Extended Data Figs. 3b and 8b were repeated twice independently with similar results.

### Reporting summary

Further information on research design is available in the Nature Portfolio Reporting Summary linked to this article.

## Supplementary information

Supplementary Information Supplementary Figs. 1 and 2 and Table 1.

Reporting Summary

## Source data

Source Data Fig. 2 Unprocessed western blots.

Source Data Fig. 3 Unprocessed western blots.

Source Data Fig. 4 Unprocessed western blots.

Source Data Fig. 5 Unprocessed western blots.

Source Data Fig. 6 Unprocessed western blots.

Source Data Fig. 7 Unprocessed western blots and gels.

Source Data Fig. 8 Unprocessed western blots.

Source Data Extended Data Fig. 3 Unprocessed gels.

Source Data Extended Data Fig. 8 Unprocessed gels.

## Data Availability

All data generated or analysed during this study are included in this published article or its Supplementary Information. Gene sequences for the following proteins from *A. thaliana* were used experimentally in this study: PUX1 (At3g27310), PUX2 (At2g01650), PUX3 (At4g22150), PUX4 (At4g04210), PUX5 (At4g15410), PUX6 (At3g21660), PUX7 (At1g14570), PUX8 (At4g11740), PUX9 (At4g00752), PUX10 (At4g10790), PUX11 (At2g43210), PUX12 (At3g23605), PUX13 (At4g23040), SP1 (At1g63900), SP2 (At3g44160), CDC48A (At3g09840), TOC159 (At4g02510), TOC33 (At1g02280), TOC120 (At3g16620), TOC132 (At2g16640), TOC34 (At5g05000), TOC75 (At3g46740), TIC110 (At1g06950), TIC40 (At5g16620), CDKA1 (At3g48750), SFR2 (At3g06510) and ubiquitin (At4g05320). Amino acid sequences of the UBX domains of the following proteins from different species were used in this study: *Oryza sativa* Os10g37630 (AAP54662), *Zea mays* GRMZM2G159538 (AQL10361), *Marchantia polymorpha* Mapoly0001s0291 (PTQ50274), *Chlamydomonas reinhardtii* Cre03.g200100 (A0A2K3DZI1), *Saccharomyces cerevisiae* Ubx2 (Q04228) and *Homo sapiens* UBXD8/FAF2 (Q96CS3). Sequences were obtained from the TAIR (https:// www.arabidopsis.org/), Phytozome (https://phytozome.jgi.doe.gov/pz/portal.html), Ensembl Plants (https://plants.ensembl.org/index.html), Uniprot (https://www.uniprot.org/) or National Center for Biotechnology Information (https://www.ncbi.nlm.nih.gov/) databases. Source data are provided with this paper.
