# Supplementary material for: Recruitment of Cdc48 to chloroplasts by a UBX-domain protein in chloroplast-associated protein degradation
Source: Nat Plants. 2024 Aug 19;10(9):1400–17. doi: 10.1038/s41477-024-01769-x (PMC11410653; doi:10.1038/s41477-024-01769-x)
Supplement: Supplementary file 1 — Supplementary Figs. 1 and 2 and Table 1. [file 41477_2024_1769_MOESM1_ESM.pdf]

nature plants

Supplementary information https://doi.org/10.1038/s41477-024-01769-x

Recruitment of Cdc48 to chloroplasts by a UBX-domain protein in chloroplast-associated protein degradation

In the format provided by the authors and unedited

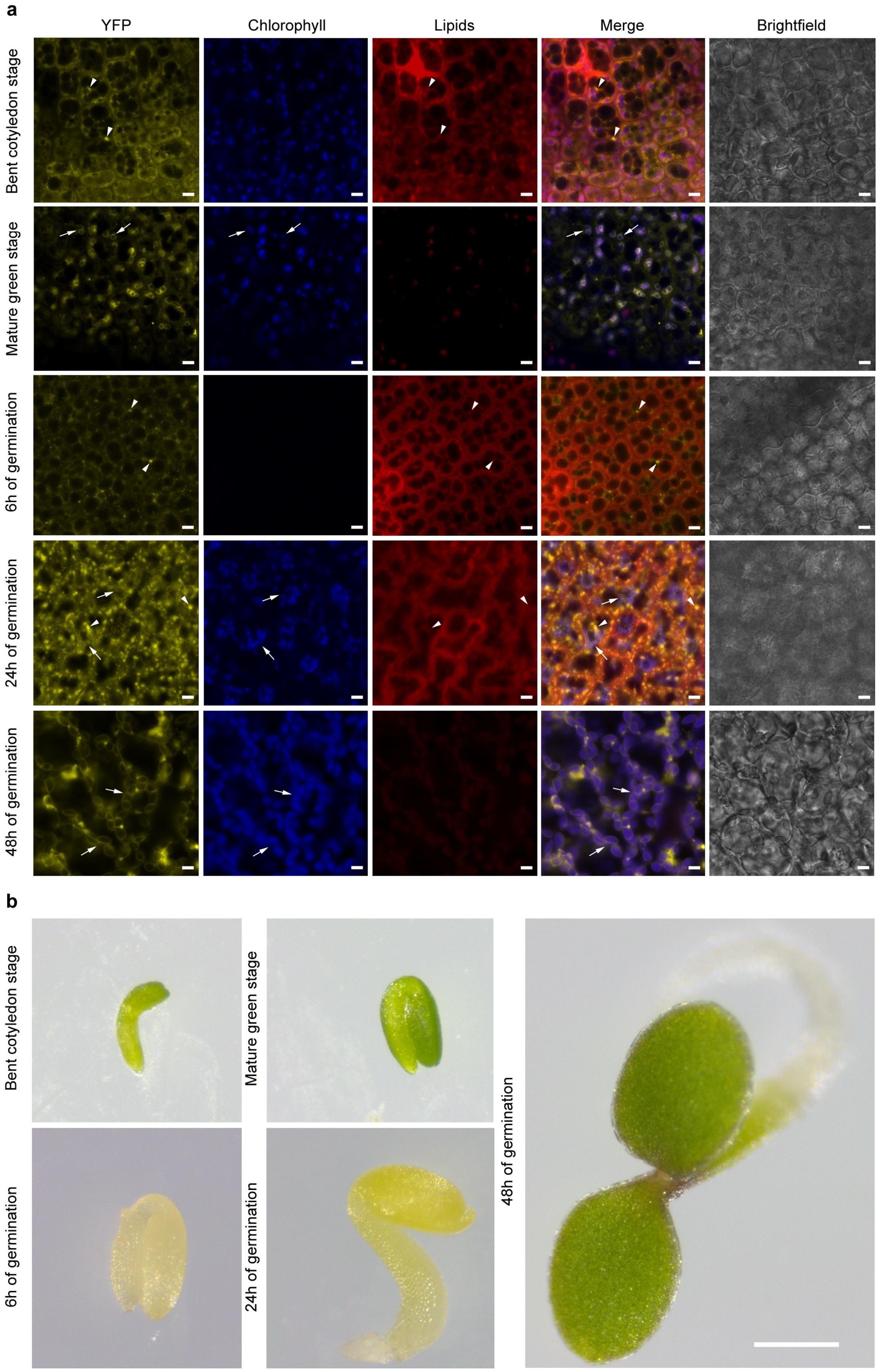
a
YFP
Chlorophyll
Lipids
Merge
Brightfield
Bent cotyledon stage
Mature green stage
6h of germination
24h of germination
48h of germination
b
Bent cotyledon stage
Mature green stage
6h of germination
24h of germination
48h of germination

Supplementary Fig. 1 | Analysis of the localization of PUX10 in lipid droplets during early development. a, Localization of PUX10 during different stages of embryogenesis and seed germination. Stable *Arabidopsis* transgenic plants expressing a construct encoding PUX10-YFP driven by the CaMV *35S* promoter (see Extended data Fig. 2) were analyzed by confocal microscopy. Cotyledon epidermises were imaged at different stages of embryogenesis (bent cotyledon stage and mature green stage) and seed germination (6, 24 and 48 h of germination). Lipid droplets (LDs) were stained with Nile Red (red). Chlorophyll autofluorescence was imaged to localize the chloroplasts (blue). Colocalization of PUX10-YFP with LDs is indicated with arrowheads; colocalization of PUX10-YFP with chloroplasts is indicated with complete arrows. Representative images are presented. In each case, YFP fluorescence, chlorophyll autofluorescence, Nile Red fluorescence, merged (combining YFP, chlorophyll and Nile Red fluorescence), and brightfield images are shown. Bars = 5 µm. **b,** Representative images of the different embryo morphogenesis and seed germination stages analyzed in **a**. The images were taken by dissection microscopy. The settings were identical in each case. Bar = 50 µm.

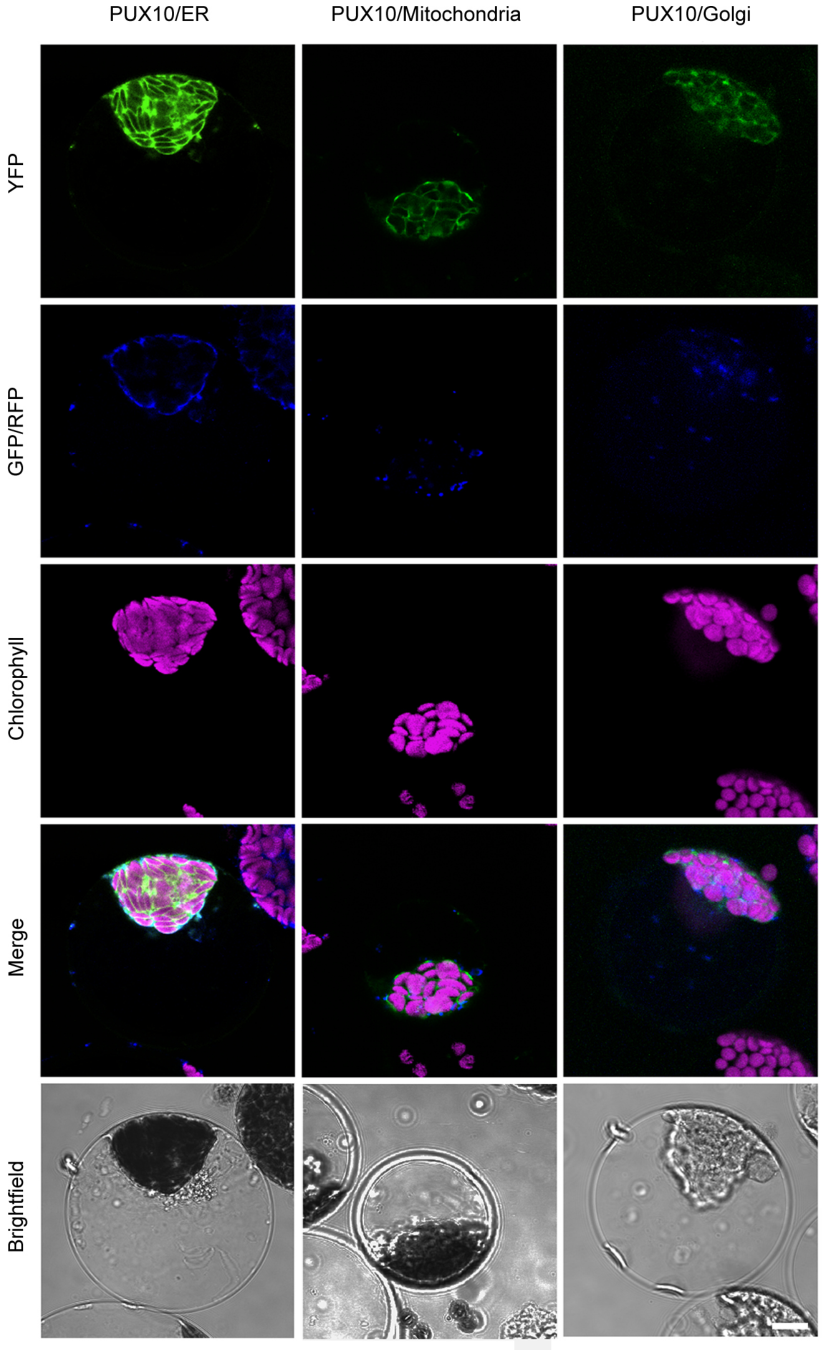

Supplementary Fig. 2 | Analysis of possible localization of PUX10 in other subcellular compartments. The PUX10-YFP construct (see Fig. 1b) was used to transiently transform protoplasts isolated from *Arabidopsis* lines stably expressing genetically-encoded organelle markers for the ER, mitochondria and Golgi. The fluorescence signals were visualized by confocal microscopy, and representative

protoplasts are presented. Both the ER and mitochondria were marked by GFP, while the Golgi was marked by RFP. In each case, YFP and GFP/RFP fluorescence, chlorophyll autofluorescence, merged YFP/GFP/RFP and chlorophyll fluorescence, and brightfield images are shown. Similar localization of the fluorescence signals was observed in 5 to 8 independent protoplasts. The exposure times and gain settings were identical in each case. Bar = 10 µm.

Supplementary Table 1: Primers used during the course of the study.

(A) Primers used to generate various constructs.

Primer name	Primer sequence (5' to 3')*	Used to generate…	Comments
PUX10-BglII-F	AAAGATCTATGGTTGATACTGTTGACAAATTAGG	pSAT4(A)-nEYFP-PUX10; pSAT4(A)-cEYFP-PUX10	PUX10-nYFP/ PUX10-cYFP<br><br>In Fig. 4, 5, Extended data Fig. 7
PUX10-SalI-ns-R	AAGTCGACAGTTGATCTCGATGAAGAGACTTGC		
ΔUBX-XhoI-F	AACTCGAGATGGTTGATACTGTTGACAAATTAGG	pSAT4(A)-cEYFP- ΔUBX	UBX-cYFP<br><br>In Fig. 4
ΔUBX-EcoRI-R	AAGAATTCAGGTTCTTCTCCAAGAGCAAGAG		
SFR2-Kpn-F	GGTACCAACTAGAAAGATCCGGTG	pSAT4(A)-nEYFP-SFR2	SFR2-nYFP<br><br>In Fig. 5
SFR2-XmaI-ns-R	CCCGGGGTCAAAGGGTGAGGCTAA		
ΔUBA-attB1-F	GGGGACAAGTTTGTACAAAAAAGCAGGCTCCATGTCTTCTGATCAAGACGCTTCG	p2GWY7-ΔUBA; pK7YWG2-ΔUBA	ΔUBA-YFP<br><br>In Fig. 1, 3, 6, Extended data Fig. 4
ΔUBA-attB2-ns-R	GGGGACCACTTTGTACAAGAAAGCTGGGTTGTTGATCTCGATGAAGAGACTTGC		
ΔUBX-attB1-F	GGGGACAAGTTTGTACAAAAAAGCAGGCTCCATGGTTGATACTGTTGACAAATTAGG	p2GWY7-ΔUBX; pK7YWG2-ΔUBX	ΔUBX-YFP<br><br>In Fig. 1, 3, 4, Extended data Fig. 4
ΔUBX-attB2-ns-R	GGGGACCACTTTGTACAAGAAAGCTGGGTTAGGTTCTTCTCCAAGAGCAAGAG		
ΔTM-attB1-1F	GGGGACAAGTTTGTACAAAAAAGCAGGCTCCATGGTTGATACTGTTGACAAATTAGG	p2GWY7-ΔTM1/2; pK7YWG2-ΔTM1/2	ΔTM1/2-YFP<br><br>In Fig. 1, 3, Extended data Fig. 4
ΔTM-1R	GAGAATACGACGGTAAAGTAATAATCCT		
ΔTM-2F	TACTTTACCGTCGTATTCTCTTGGTATG		

ΔTM-attB2-ns-2R	GGGGACCACTTTGTACAAGAAAGCTGGGTTGTTGATCTCGATGAAGAGACTTGC		
PUX10-FLAG-F	TACAAAGACGATGACGACAAAATGGTTGATACTGTTGACAAATTAGG	pK7YWG2-FLAG-PUX10	FLAG-PUX10-YFP<br><br>In Fig. 2
attB1-FLAG	GGGGACAAGTTTGTACAAAAAAGCAGGCTCCATGGACTACAAAGACGATGACGAC		
PUX10-Pro-HindIII-F	AAAAGCTTACATCGTCCTGCATTTTTCGATG	pPUX10-K7YWG2-PUX10	pPUX10:PUX10-YFP<br><br>In Extended data Fig. 2
PUX10-Pro-SpeI-R	AAACTAGTTTTCAGAAAACCCTAATTCGATTTTCCC		
PUX1-attB1-F	AAAAAGCAGGCTCCATGTTTGTTGATGACCCTTCTC	p2GWY7-PUX1	PUX1-YFP<br><br>In Extended data Fig. 1
PUX1-attB2-ns-R	AGAAAGCTGGGTTCATTTTAAACCACTTAGGCTTTGTGG		
PUX2-attB1-F	AAAAAGCAGGCTCCATGGATGACGTTAAAGATAAGC	p2GWY7-PUX2	PUX2-YFP<br><br>In Extended data Fig. 1
PUX2-attB2-ns-R	AGAAAGCTGGGTTCGAGAGTGGTTCACTGATC		
PUX3-attB1-F	AAAAAGCAGGCTCCATGCTAGTTTTGTTTCTGGCTAAGC	p2GWY7-PUX3	PUX3-YFP<br><br>In Extended data Fig. 1
PUX3-attB2-ns-R	AGAAAGCTGGGTTGAATTTCTGAATGACTACAGAACTTG		
PUX4-attB1-F	AAAAAGCAGGCTCCATGTCTTCGAAAGACAAGAAACC	p2GWY7-PUX4	PUX4-YFP<br><br>In Extended data Fig. 1
PUX4-attB2-ns-R	AGAAAGCTGGGTTGAATTTCTGAAGAACAACAGAGTTG		
PUX5-attB1-F	AAAAAGCAGGCTCCATGGCGACGGAGACGAACGAGAA	p2GWY7-PUX5	PUX5-YFP<br><br>In Extended data Fig. 1
PUX5-attB2-ns-R	AGAAAGCTGGGTTGAATTTCTGGATGACGACG		
PUX6-attB1-F	AAAAAGCAGGCTCCATGGACGTTAACTCGCCGGAGA	p2GWY7-PUX6	PUX6-YFP<br><br>In Extended data Fig. 1
PUX6-attB2-ns-R	AGAAAGCTGGGTTGAATTTTTGGACGAGGACAGAG		

PUX7-attB1-F	AAAAAGCAGGCTCCATGGAAGGAATGTTGTCGTC	p2GWY7-PUX7	PUX7-YFP<br><br>In Extended data Fig. 1
PUX7-attB2-ns-R	AGAAAGCTGGGTTTTCCCACGTAGCAGAGATC		
PUX8-attB1-F	AAAAAGCAGGCTCCATGGCGACACCGAATCAG	p2GWY7-PUX8	PUX8-YFP<br><br>In Extended data Fig. 1
PUX8-attB2-ns-R	AGAAAGCTGGGTTGATAAGCTCAAGGAACAATGC		
PUX9-attB1-F	AAAAAGCAGGCTCCATGGTGAGTCCGACGAGAG	p2GWY7-PUX9	PUX9-YFP<br><br>In Extended data Fig. 1
PUX9-attB2-ns-R	AGAAAGCTGGGTTCACCAGCAGTTCCAGAAAC		
PUX10-attB1-F	AAAAAGCAGGCTCCATGGTTGATACTGTTGACAAATTAG	p2GWY7-PUX10; pK7YWG2-PUX10; pK7YWG2-PUX10 modified with native promoter; pH2GW7 modified with HA tag	PUX10-YFP<br><br>In Fig. 1, 4, 5, 6, Extended data Fig. 1, 2, Supplementary Fig. 1, 2<br><br>PUX10-OX<br><br>In Fig. 2, 3, Extended data Fig. 4
PUX10-attB2-ns-R	AGAAAGCTGGGTTGTTGATCTCGATGAAGAGAC		
PUX11-attB1-F	AAAAAGCAGGCTCCATGGAAGCTCTGTCTTCTC	p2GWY7-PUX11	PUX11-YFP<br><br>In Extended data Fig. 1
PUX11-attB2-ns-R	AGAAAGCTGGGTTTCGTCTATCATTACTGTCTC		
PUX12-attB1-F	AAAAAGCAGGCTCCATGCTCGAGTGTCAATCAATG	p2GWY7-PUX12	PUX12-YFP<br><br>In Extended data Fig. 1
PUX12-attB2-ns-R	AGAAAGCTGGGTTGAGCCAAGTAACAGAAACC		

PUX13-attB1-F	AAAAAGCAGGCTCCATGGCGACACCTACTCAG	p2GWY7-PUX13	PUX13-YFP<br><br>In Extended data Fig. 1
PUX13-attB2-ns-R	AGAAAGCTGGGTTGATCAGCTCCAAGAACAATG		
attB1	GGGGACAAGTTTGTACAAAAAGCAGGCT	Complete Gateway recombination sites for cloning into pDONR201	Multiple constructs<br><br>In Extended data Fig. 1
attB2	GGGGACCACTTTGTACAAGAAAGCTGGGT		
PUX10-R409A-F	TTAGTTGCGTTCCCGAATG	p2GWY7-PUX10(mut)	PUX10(mut)-YFP<br><br>In Fig. 4
PUX10-R409A-R	CATTCGGGAACGCAACTAA		
PUX10-F450S-F	TCACAAACAGCCCAAGGAC		
PUX10-F450S-R	GTCCTTGGGCTGTTTGTGA		
PUX10-R452A-F	CTTCCCAGCGACAGTGTATG		
PUX10-R452A-R	CATACACTGTCGCTGGGAAG		
Cdc48-ΔN-attB1-F	AAAAAAGCAGGCTCCATGGAGCCTGTGAAGAGAGAG	p2GW7-Cdc48(ΔNterm)-HA	Cdc48(ΔNterm)-HA<br><br>In Fig. 4
Cdc48-ΔN-attB2-nsR	AGAAAGCTGGGTTATTGTAGAGATCATCATCGTCCC		

*Nucleotides shown in red do not corresponding to the target gene, but instead correspond to linker sequences. Restriction sequences are underlined.

(B) Primers used in RT-PCR experiments.

Primer name	Primer sequence (5' to 3')	Comments
PUX10-RT-1F	GTTTCTGGTAGCTTAGGGTTAGT	In Extended data Fig. 3
PUX10-RT-1R	TGATCGGCTTCAAGAGCAG	
PUX10-RT-2F	GTGGTGGAACGCTTTGTA	
PUX10-RT-2R	AAGTCGACAGTTGATCTCGATGAAGAGACTTGC	
Toc159-F	TGTTGTAGGAGACGCAGAGG	In Fig. 7
Toc159-R	ACTTATTATGCACATCAGGGATGT	
Toc132-F	GATGGGACTGAGTTTGTGGTTAG	
Toc132-R	CCTCTTGTTCTGTCTGTATGCC	
Toc75-F	GTTGGGTTAATGGTACAGTCG	
Toc75-R	ATCTCAACAATAATGCCCCCT	
Toc34-F	TTGTCGGTGCTATAACTGATGC	
Toc34-R	CTTGCTAAACCGGAGTCTCG	
Tic110-F	CACAACAAAGAGAGACGATTCAG	
Tic110-R	CAAATCATTCAGCGACAAGAC	
Tic40-F	TCTCGTCGGACTCCTAATATTGT	
Tic40-R	CTTTCTTCTCTTTGCTTGCCT	
SP1-F	GGTACAAGATAGTGCGTTGATG	In Extended data Fig. 8
SP1-R	CTGCAGTCAGTGACGATATGTCTTAAC	
eIF4E1-F	AAACAATGGCGGTAGAAGACACTC	In Fig. 7, Extended data Fig. 3, 8
eIF4E1-R	AAGATTTGAGAGGTTTCAAGCGGTGTAAG	

(C) Primers used to genotype T-DNA mutants.

Primer name	Primer sequence (5' to 3')	Comments
PUX10-SAIL-F	GTTTCTGGTAGCTTAGGGTTAGT	In Extended data Fig. 3
PUX10-SAIL-R	TGATCGGCTTCAAGAGCAG	
LB1	GCCTTTTCAGAAATGGATAAATAGCCTTGCTTCC	
PUX10-Wisc-F	GAGAGATACTAAACTTGGTGCC	
PUX10-Wisc-R	ACATCTAGTATGTATGAATGTGAGG	
WiscDsLoxLB	AACGTCCGCAATGTGTTATTAAGTTGTC	
