# Supplementary material for: Recruitment of Cdc48 to chloroplasts by a UBX-domain protein in chloroplast-associated protein degradation
Source: Nat Plants. 2024 Aug 19;10(9):1400–17. doi: 10.1038/s41477-024-01769-x (PMC11410653; doi:10.1038/s41477-024-01769-x)
Supplement: Supplementary file 3 — Unprocessed western blots. [file 41477_2024_1769_MOESM3_ESM.pdf]

Fig. 2a

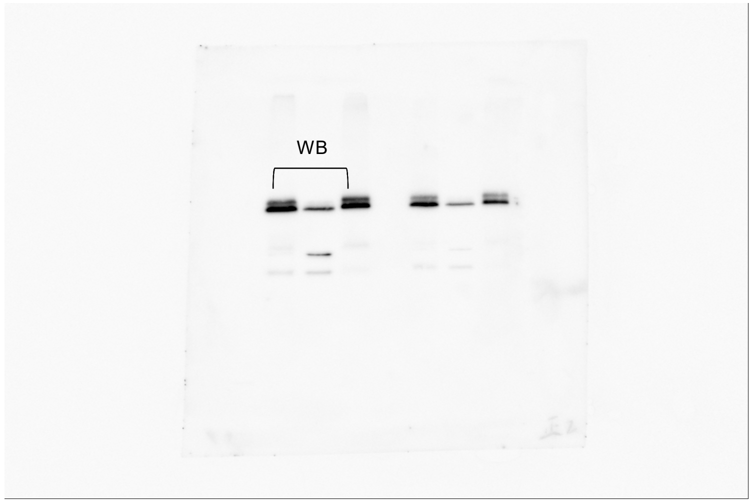
WB

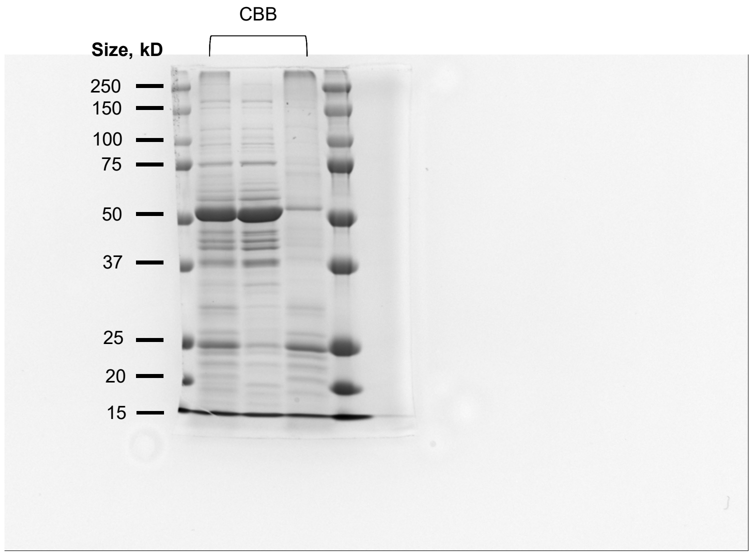
CBB
Size, kD
250
150
100
75
50
37
25
20
15

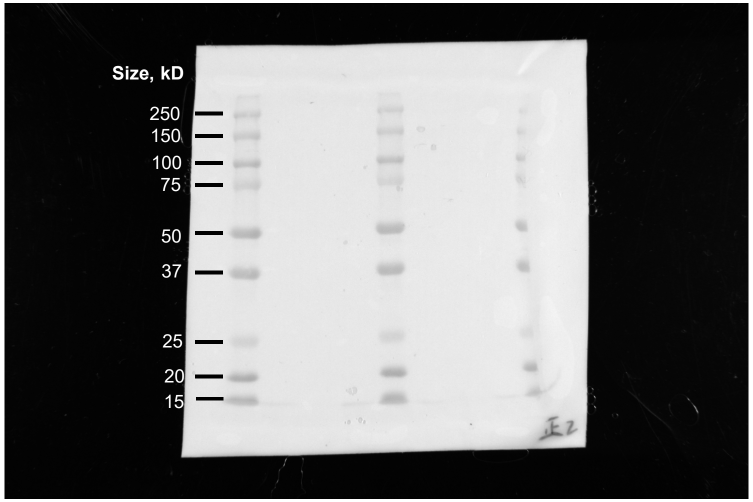
Size, kD
250
150
100
75
50
37
25
20
15

Fig. 2b

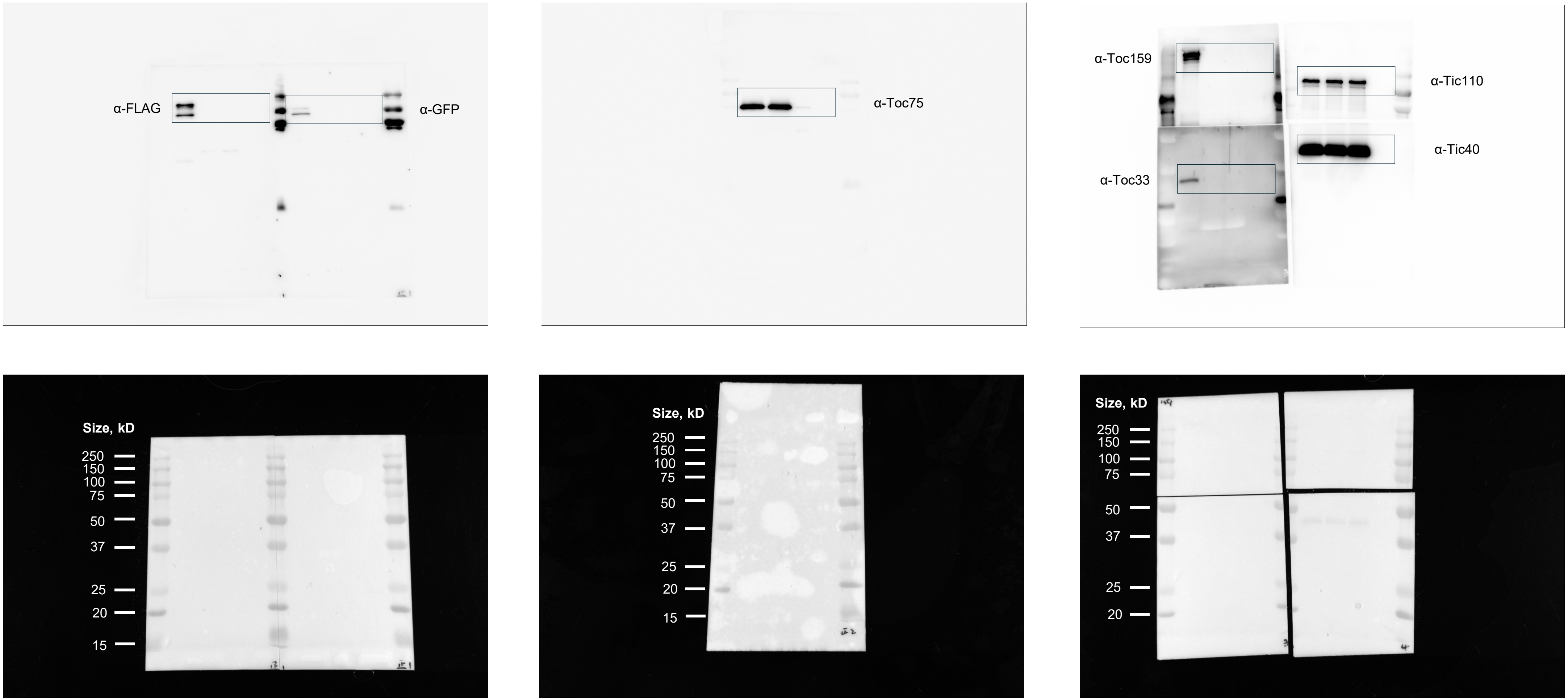

One single membrane with duplicate identical loadings, but cut into two for probing with two different antibodies.

One single membrane with duplicate identical loadings, but cut into four for probing with four different antibodies.

Note: Multiple exposure times were recorded in each case, but for simplicity of presentation just a single exposure time is shown here.
