# Supplementary material for: Recruitment of Cdc48 to chloroplasts by a UBX-domain protein in chloroplast-associated protein degradation
Source: Nat Plants. 2024 Aug 19;10(9):1400–17. doi: 10.1038/s41477-024-01769-x (PMC11410653; doi:10.1038/s41477-024-01769-x)

Fig. 3b,c

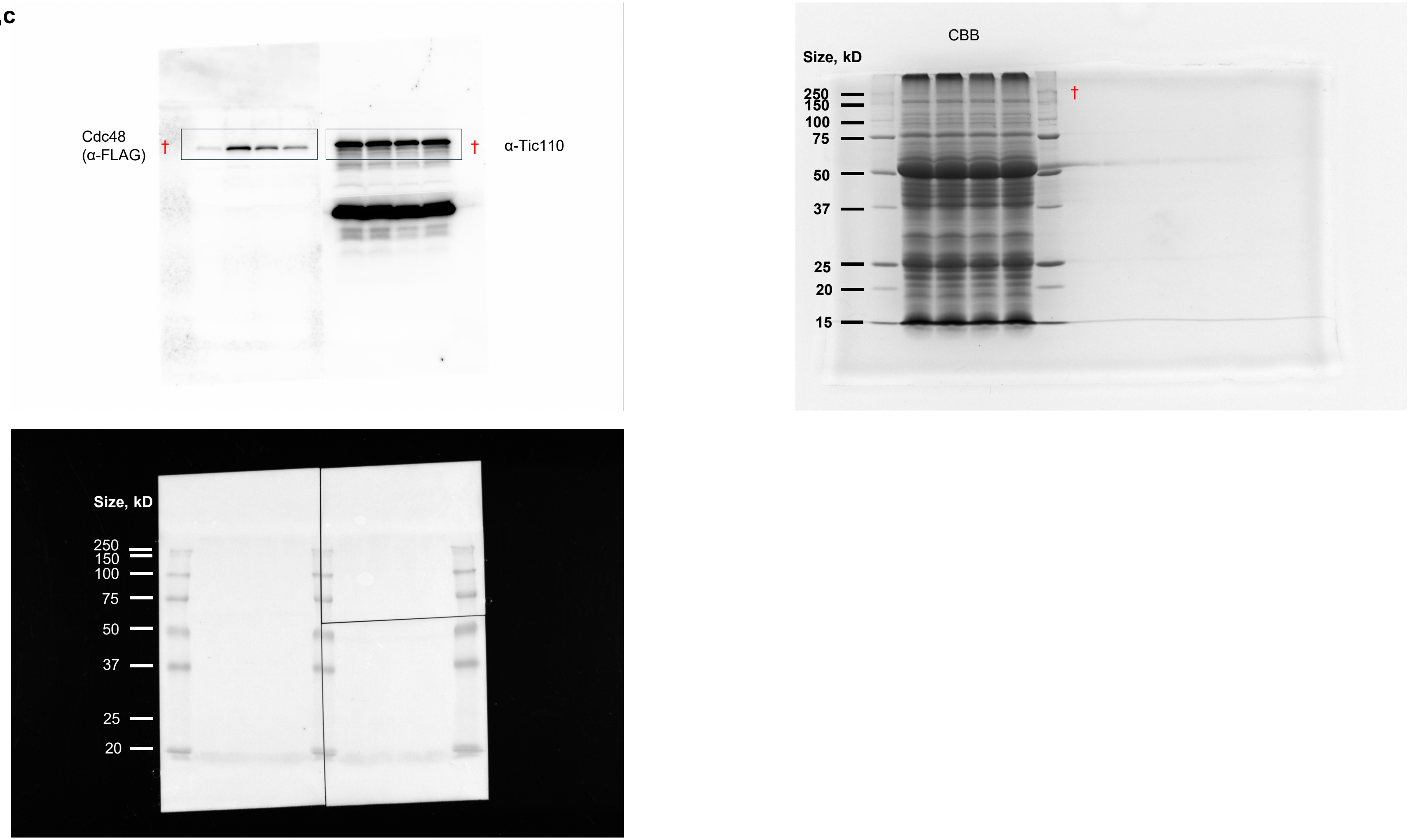

One single membrane with duplicate identical loadings, but cut into three for probing with three different antibodies.
The lower-right panel was not included in Fig. 3b,c.

Note: Replicate results using identical sample loadings for quantification purposes are shown, here and on the following two pages; the results shown in Fig. 3b are marked with a dagger (†), although the exposure times may differ.
Multiple exposure times were recorded in each case, but for simplicity of presentation just a single exposure time is shown here.

Fig. 3b,c continued

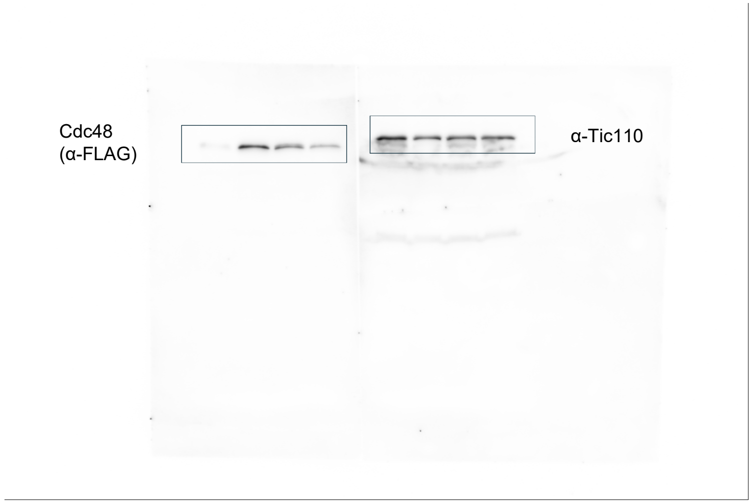

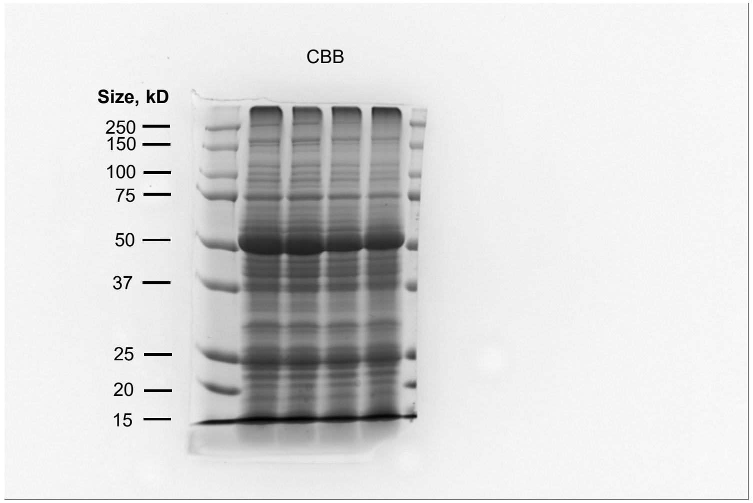

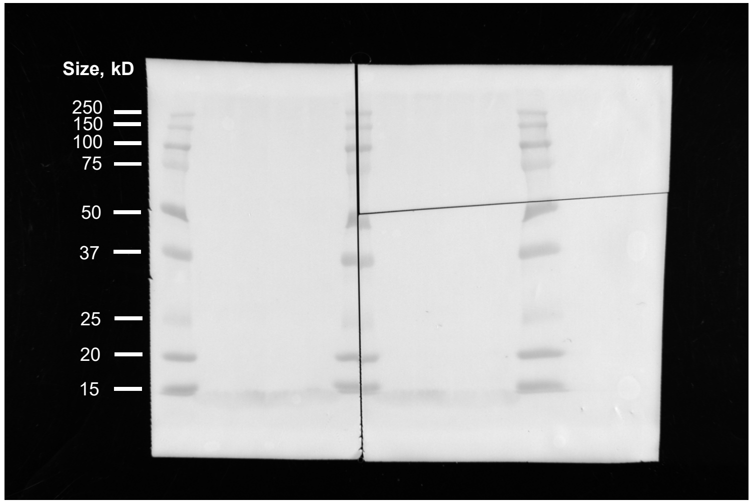

One single membrane with duplicate identical loadings, but cut into three for probing with three different antibodies.
The lower-right panel was not included in Fig. 3c.

Fig. 3b,c continued

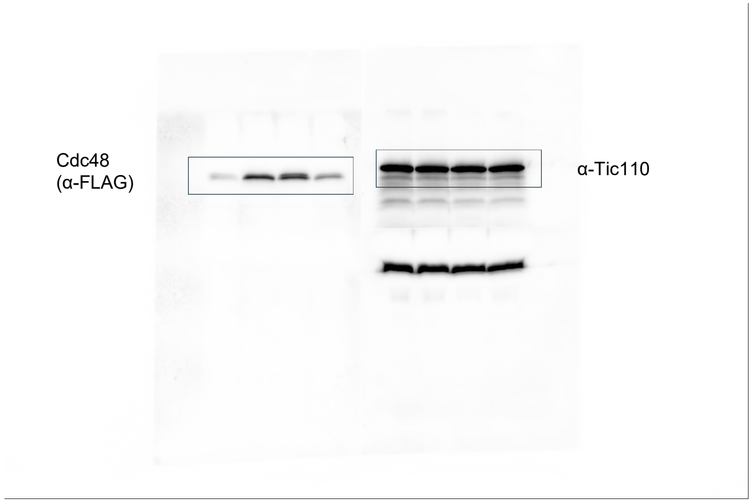

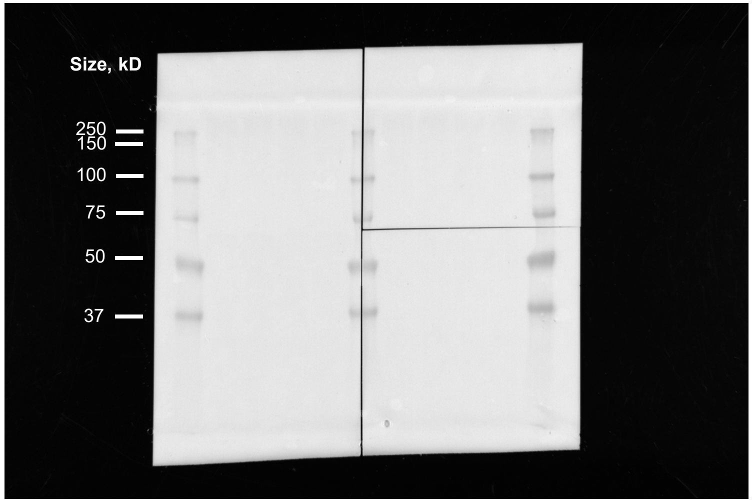

One single membrane with duplicate identical loadings, but cut into three for probing with three different antibodies.
The lower-right panel was not included in Fig. 3c.

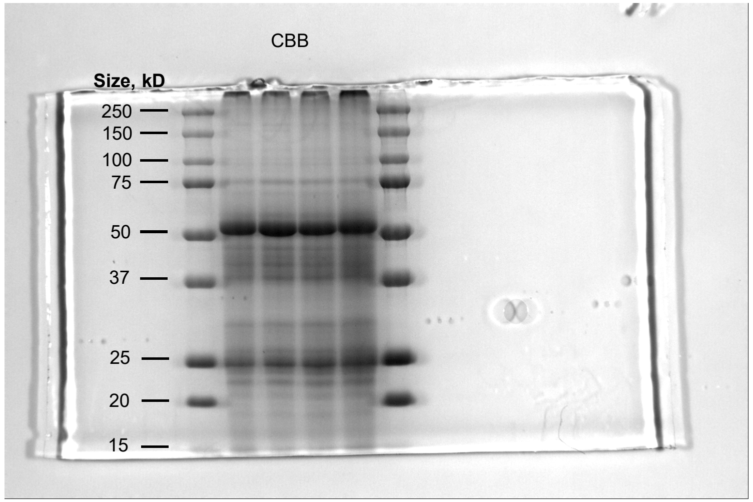

Supplement: Supplementary file 4 — Unprocessed western blots. [file 41477_2024_1769_MOESM4_ESM.pdf]
