# Supplementary material for: Recruitment of Cdc48 to chloroplasts by a UBX-domain protein in chloroplast-associated protein degradation
Source: Nat Plants. 2024 Aug 19;10(9):1400–17. doi: 10.1038/s41477-024-01769-x (PMC11410653; doi:10.1038/s41477-024-01769-x)
Supplement: Supplementary file 5 — Unprocessed western blots. [file 41477_2024_1769_MOESM5_ESM.pdf]

Fig. 4d

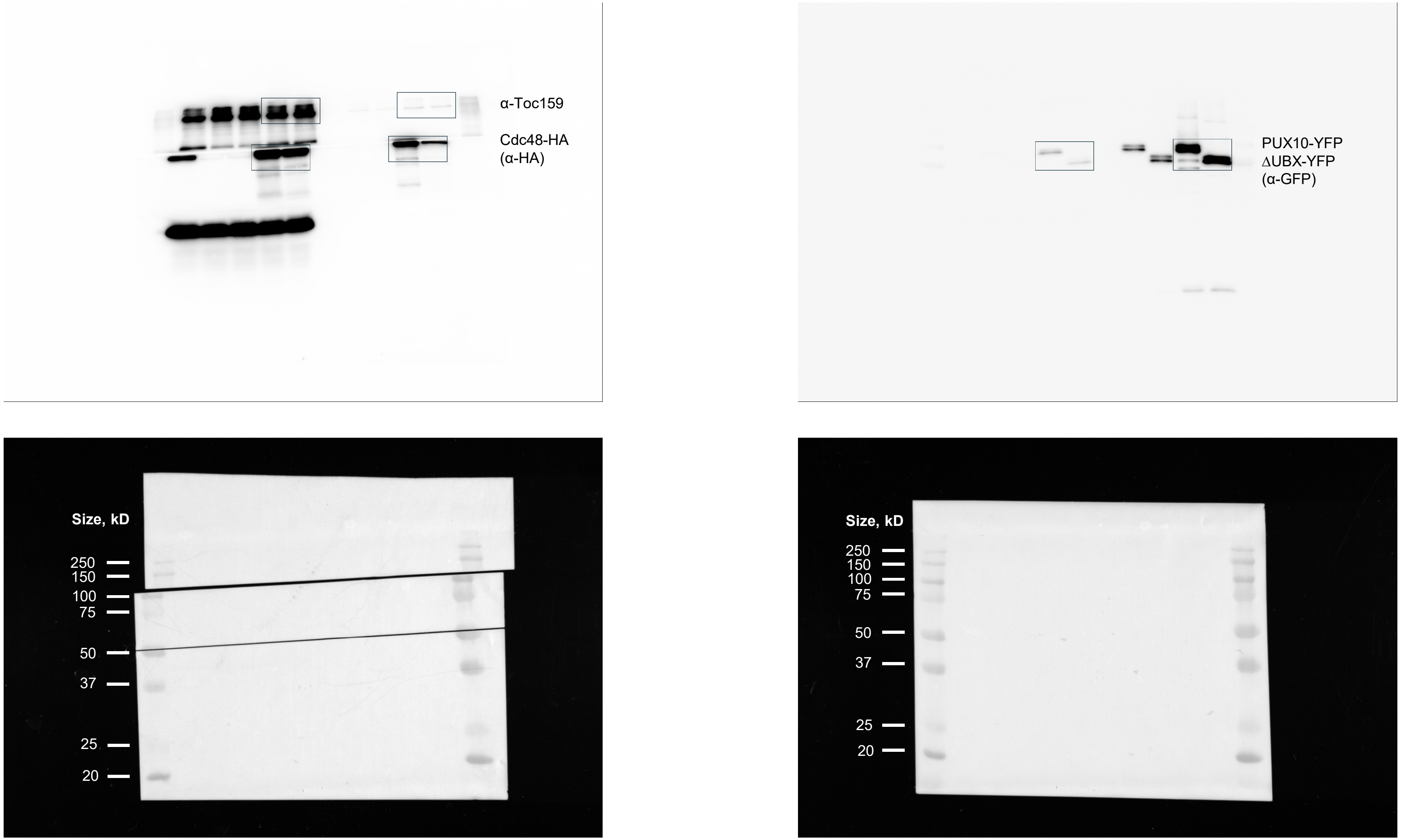

One single membrane but cut for probing with three different antibodies.
The lower panel was not included in Fig. 4d.

Note: Multiple exposure times were recorded in each case, but for simplicity of presentation just a single exposure time is shown here.

Fig. 4e

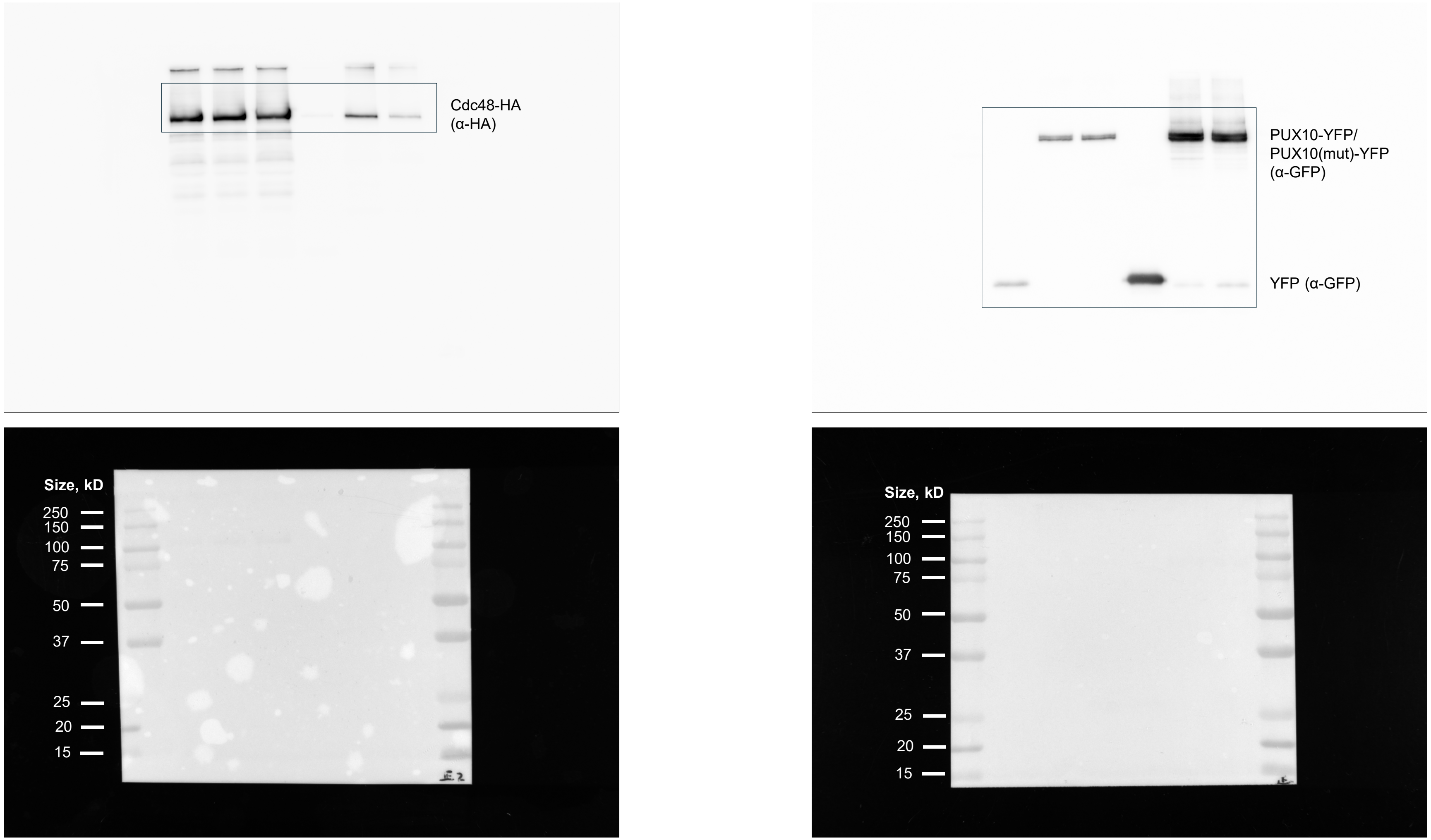

Note: Multiple exposure times were recorded in each case, but for simplicity of presentation just a single exposure time is shown here.

Fig. 4f

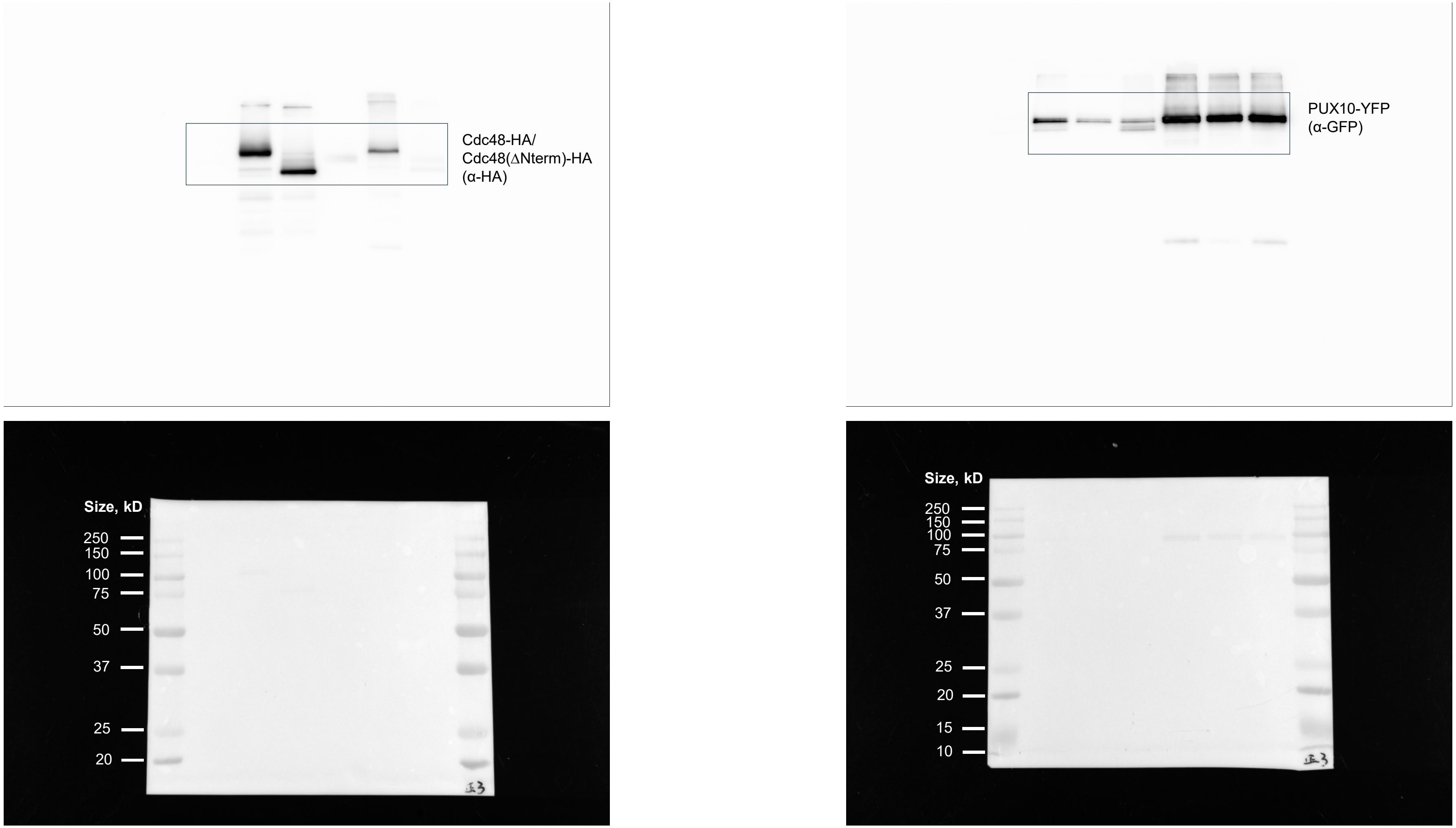

Note: Multiple exposure times were recorded in each case, but for simplicity of presentation just a single exposure time is shown here.
