# Supplementary material for: Recruitment of Cdc48 to chloroplasts by a UBX-domain protein in chloroplast-associated protein degradation
Source: Nat Plants. 2024 Aug 19;10(9):1400–17. doi: 10.1038/s41477-024-01769-x (PMC11410653; doi:10.1038/s41477-024-01769-x)
Supplement: Supplementary file 6 — Unprocessed western blots. [file 41477_2024_1769_MOESM6_ESM.pdf]

Fig. 5b

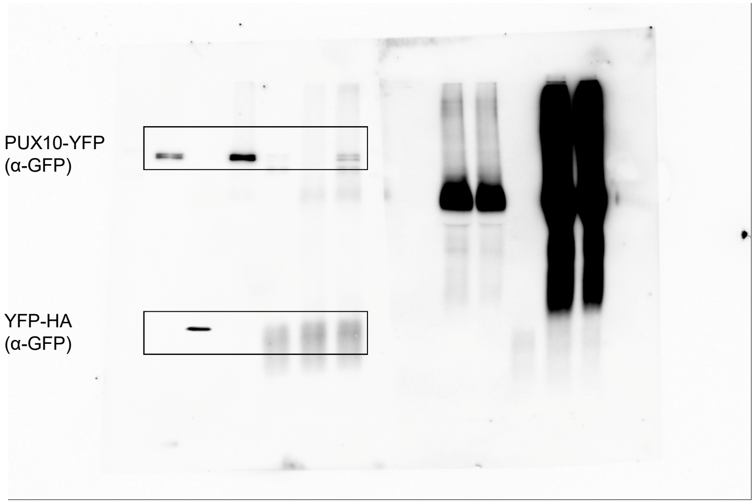

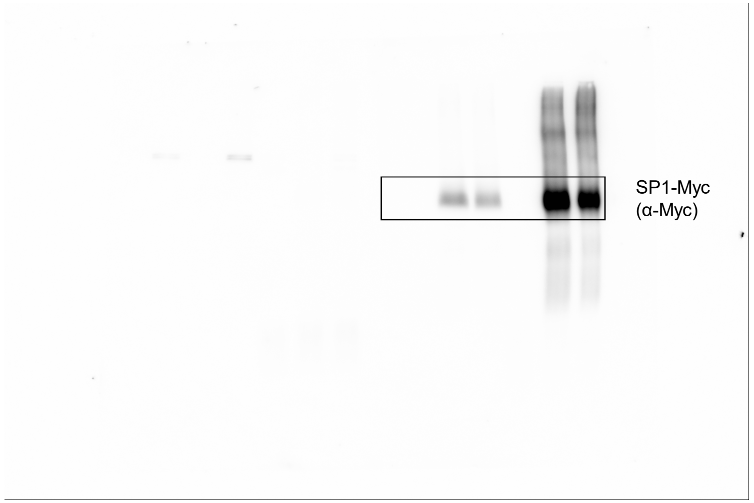

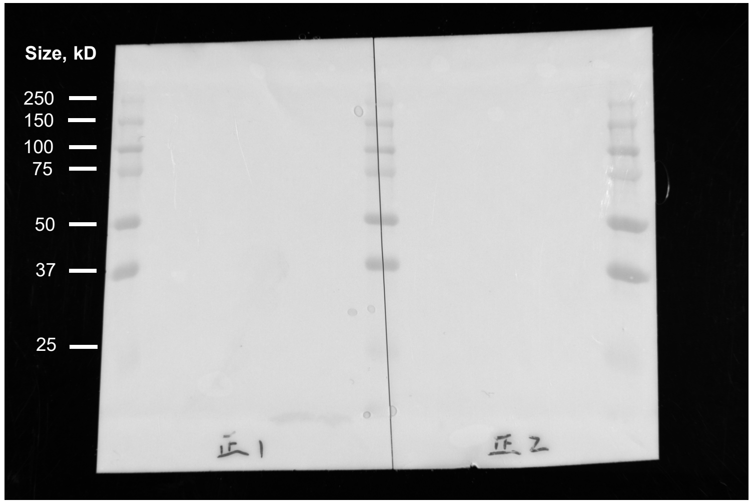

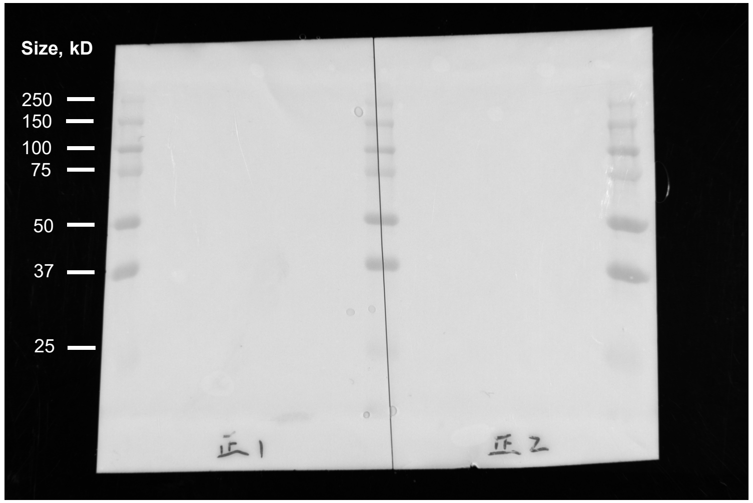

One single membrane with duplicate identical loadings, but cut into two for probing with two different antibodies.

This is the same membrane as the one on the left but with shorter exposure time.

Fig. 5c

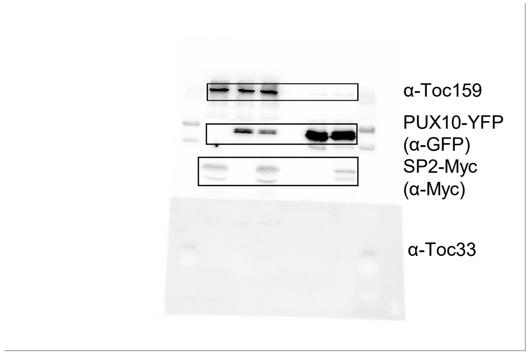

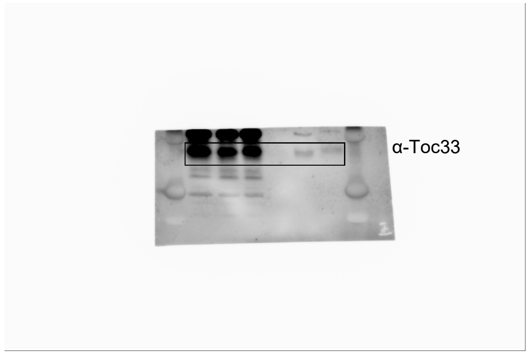

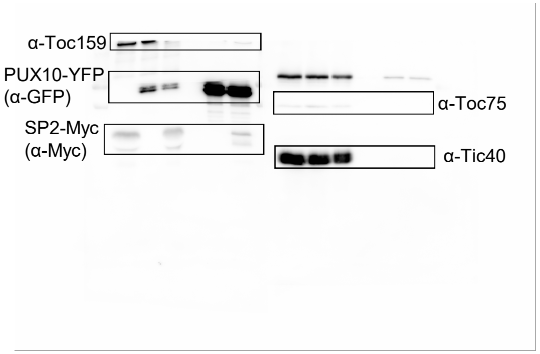

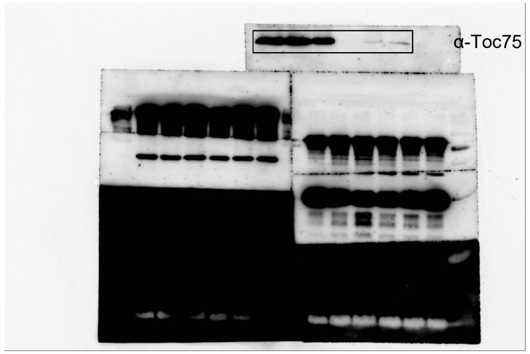

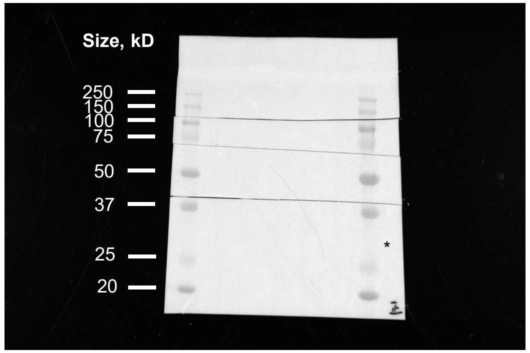

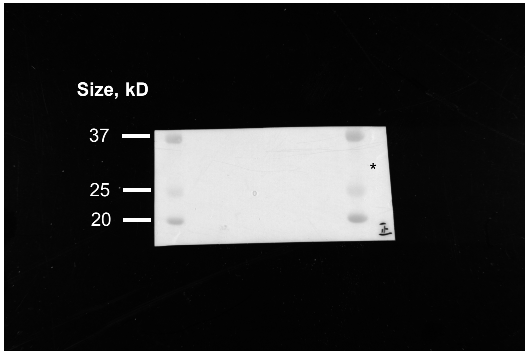

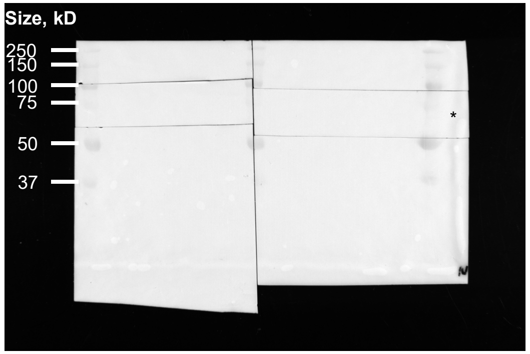

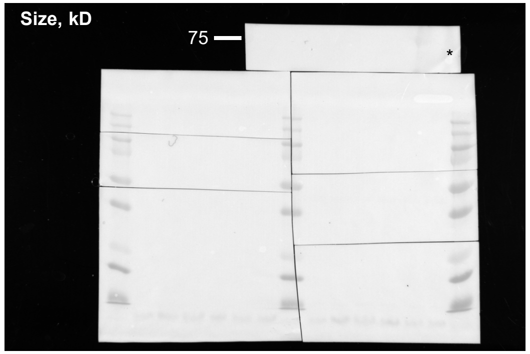

One single membrane but cut for probing with four different antibodies.

- One piece (*) was reprobed with a different Toc33 antibody since the signal was too weak.

- The indicated membrane piece on the left (*) was reprobed with a different Toc33 antibody.

One single membrane with duplicate identical loadings, but cut into six for probing with six different antibodies.
The upper-right panel was not included in Fig. 5c

- One piece (*) was reprobed with freshly-prepared Toc75 antibody since the signal was too weak.

- The indicated membrane piece on the left (*) was reprobed with freshly-prepared Toc75 antibody.
  The membrane below was for a different experiment and not related to this assay.

Note: Multiple exposure times were recorded in each case, but for simplicity of presentation just a single exposure time is shown here.
