# Supplementary material for: Recruitment of Cdc48 to chloroplasts by a UBX-domain protein in chloroplast-associated protein degradation
Source: Nat Plants. 2024 Aug 19;10(9):1400–17. doi: 10.1038/s41477-024-01769-x (PMC11410653; doi:10.1038/s41477-024-01769-x)
Supplement: Supplementary file 7 — Unprocessed western blots. [file 41477_2024_1769_MOESM7_ESM.pdf]

Fig. 6a

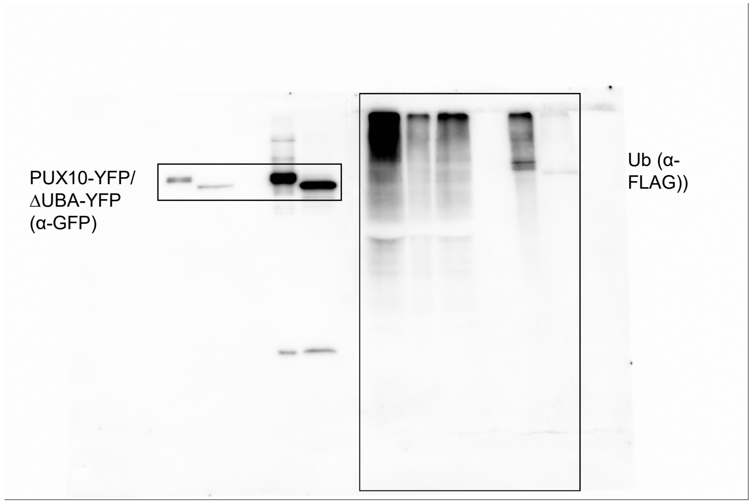

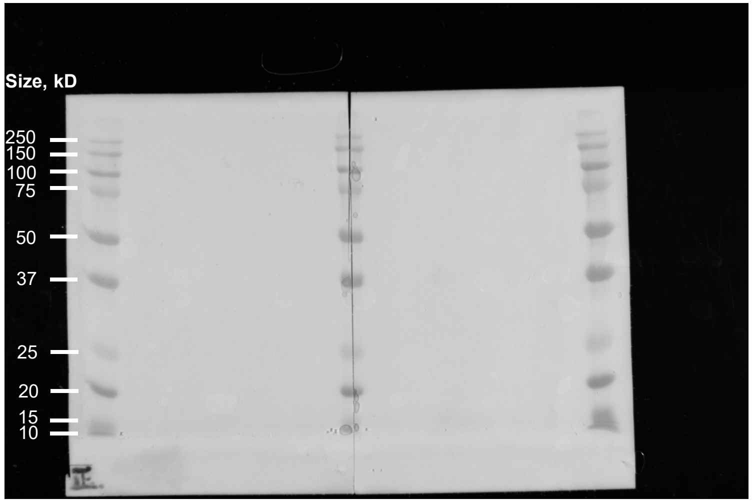

One single membrane with duplicate identical loadings, but cut into two for probing with two different antibodies.

Note: Multiple exposure times were recorded in each case, but for simplicity of presentation just a single exposure time is shown here.

Fig. 6b

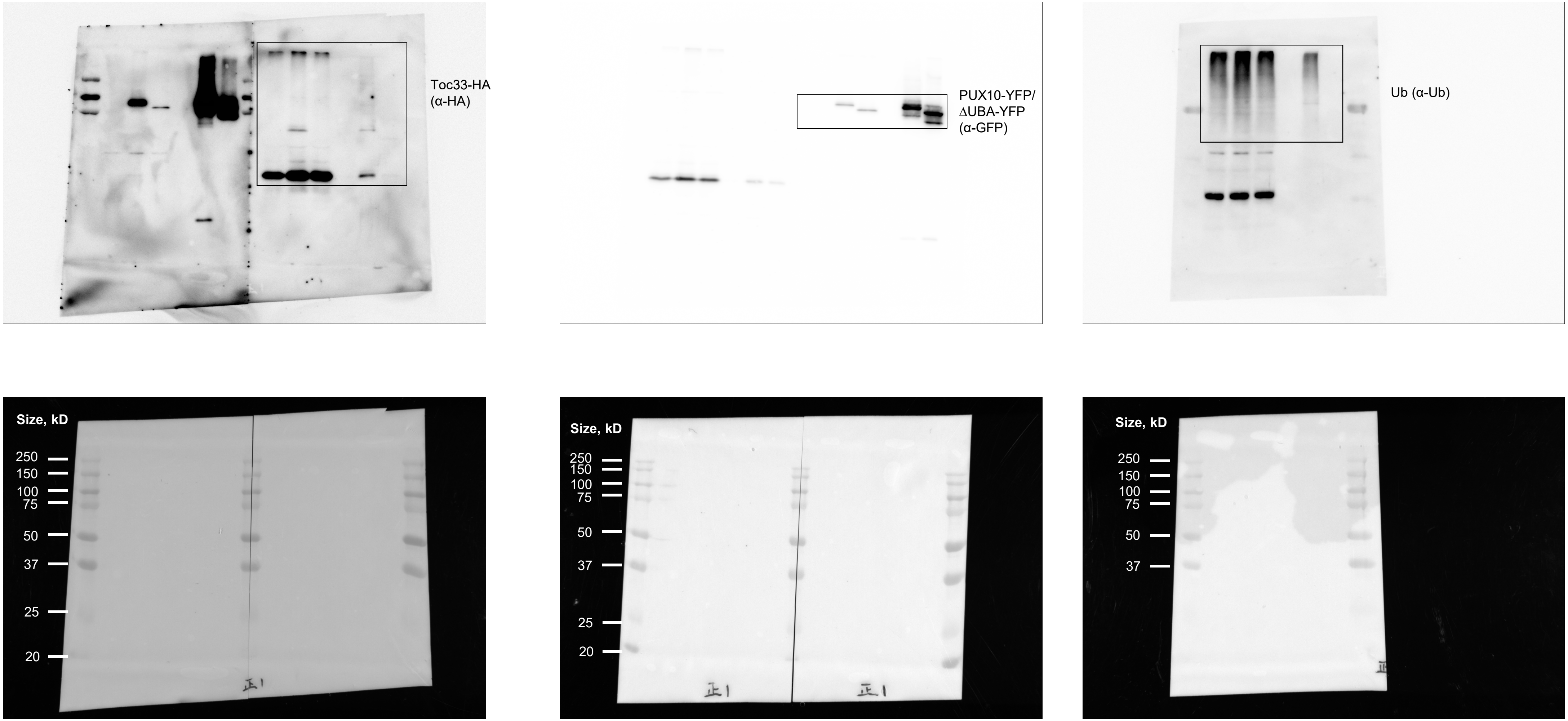

One single membrane with duplicate identical loadings, but cut into two for probing with two different antibodies.
The left panel was not included in Fig. 6b.

One single membrane with duplicate identical loadings, but cut into two for probing with two different antibodies.
The left panel was not included in Fig. 6b.

Note: Multiple exposure times were recorded in each, but for simplicity of presentation just a single exposure time is shown here.

Fig. 6c

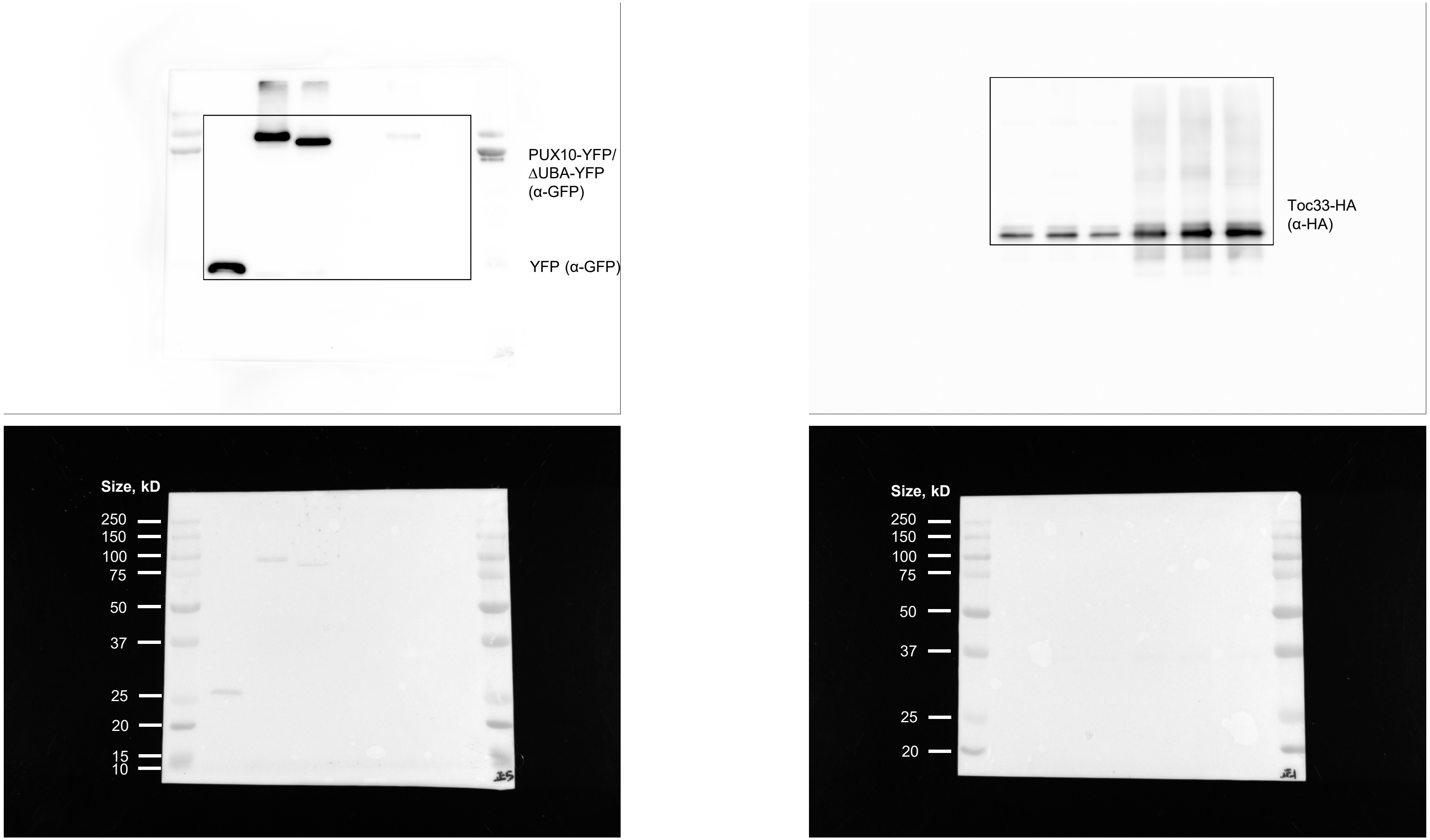

Note: Multiple exposure times were recorded in each case, but for simplicity of presentation just a single exposure time is shown here.
