# Supplementary material for: Recruitment of Cdc48 to chloroplasts by a UBX-domain protein in chloroplast-associated protein degradation
Source: Nat Plants. 2024 Aug 19;10(9):1400–17. doi: 10.1038/s41477-024-01769-x (PMC11410653; doi:10.1038/s41477-024-01769-x)
Supplement: Supplementary file 8 — Unprocessed western blots and gels. [file 41477_2024_1769_MOESM8_ESM.pdf]

**Fig. 7c,d**

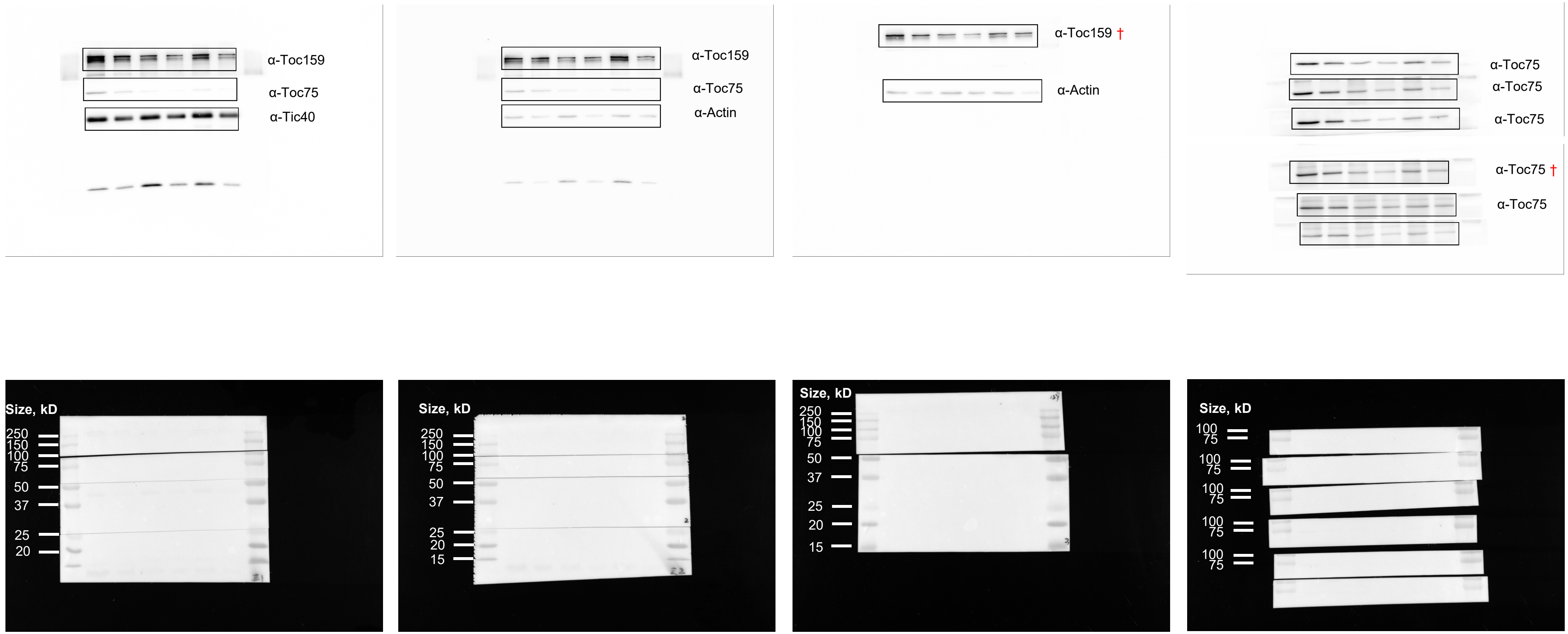


One single membrane but cut for probing with four different antibodies.
The lower panel was not included in Fig. 7d.

One single membrane but cut for probing with four different antibodies.
The lower panel was not included in Fig. 7d.

One single membrane but cut for probing with two different antibodies.

Six cropped membranes loaded with the same samples were reprobed with Toc75 antibody.
The upper three images were recorded with a longer exposure time.
The lower panel was not included in Fig. 7d.

Note: Replicate results using identical sample loadings for quantification purposes, for Toc159 and Toc75, are shown; a longer exposure time was used for Toc75 quantification; the results shown in Fig. 7c are marked with a dagger (†), although the exposure times may differ.

Multiple exposure times were recorded in each case, but for simplicity of presentation just a single exposure time is shown here.

## Fig. 7c,d continued

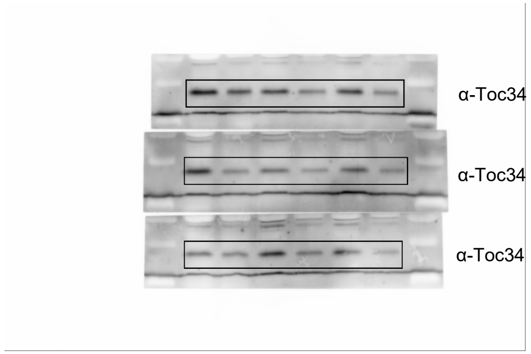


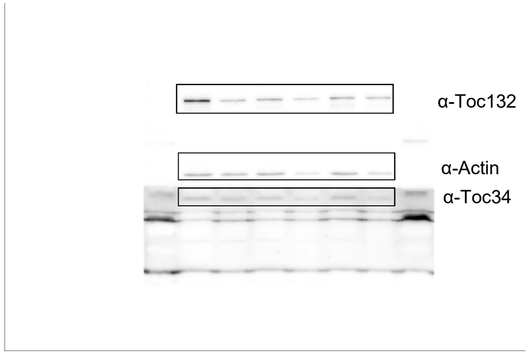


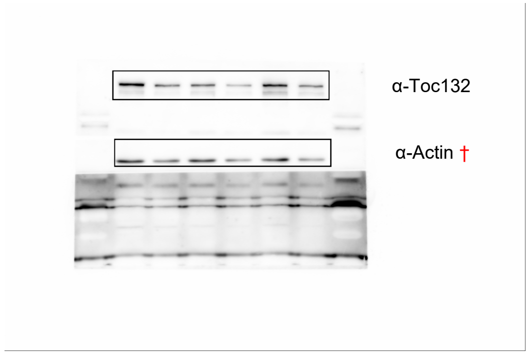


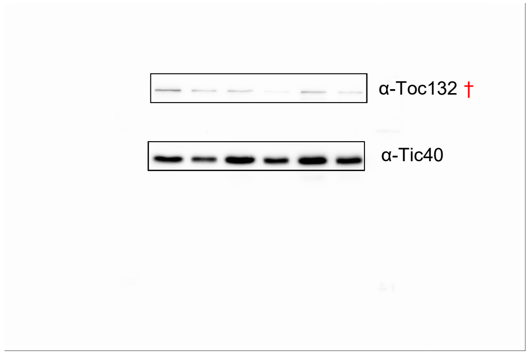


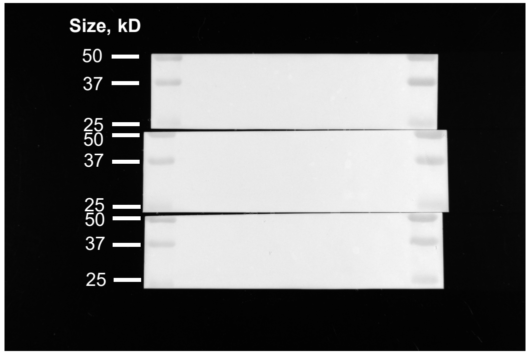


Three cropped membranes loaded with the same samples were reprobed with Toc34 antibody.

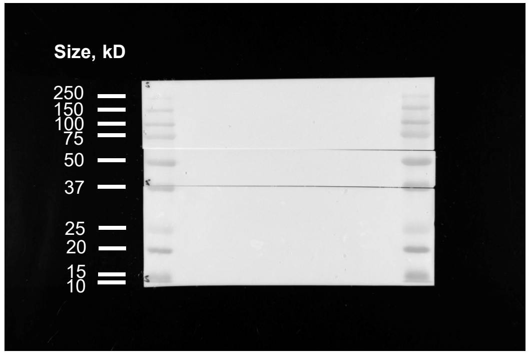


One single membrane but cut for probing with three different antibodies.

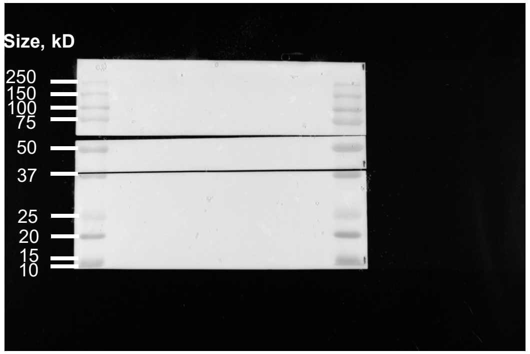


One single membrane but cut for probing with three different antibodies.
The lower panel was not included in Fig. 7d.

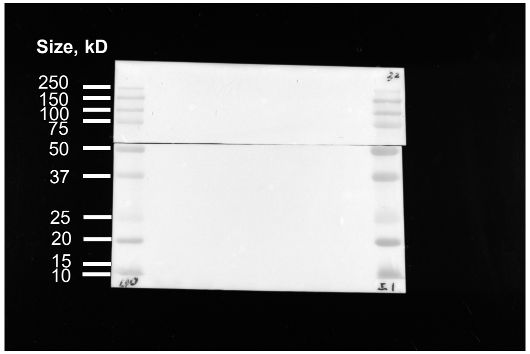


One single membrane but cut for probing with two different antibodies.

Note: Replicate results using identical sample loadings for quantification purposes, for Toc34 and Toc132, are shown; the results shown in Fig. 7c are marked with a dagger (†), although the exposure times may differ.
Replicate results using identical sample loadings for quantification purposes, for Actin, are shown here and on the previous page; the result shown in Fig. 7c is marked with a dagger (†), although the exposure time may differ.
Multiple exposure times were recorded in each, but for simplicity of presentation just a single exposure time is shown here.

## Fig. 7c,d continued

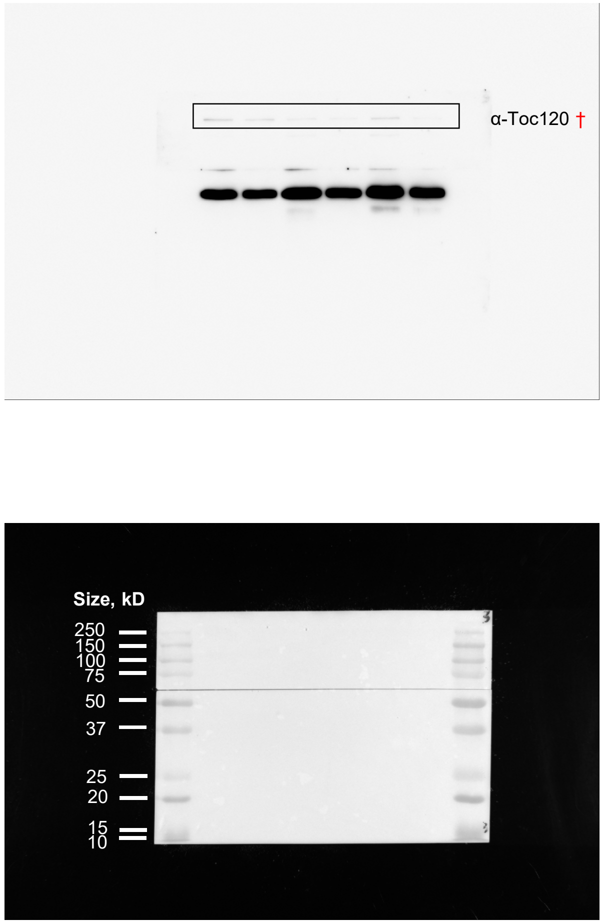


One single membrane but cut for probing with two different antibodies.
The lower panel was not included in Fig. 7d.

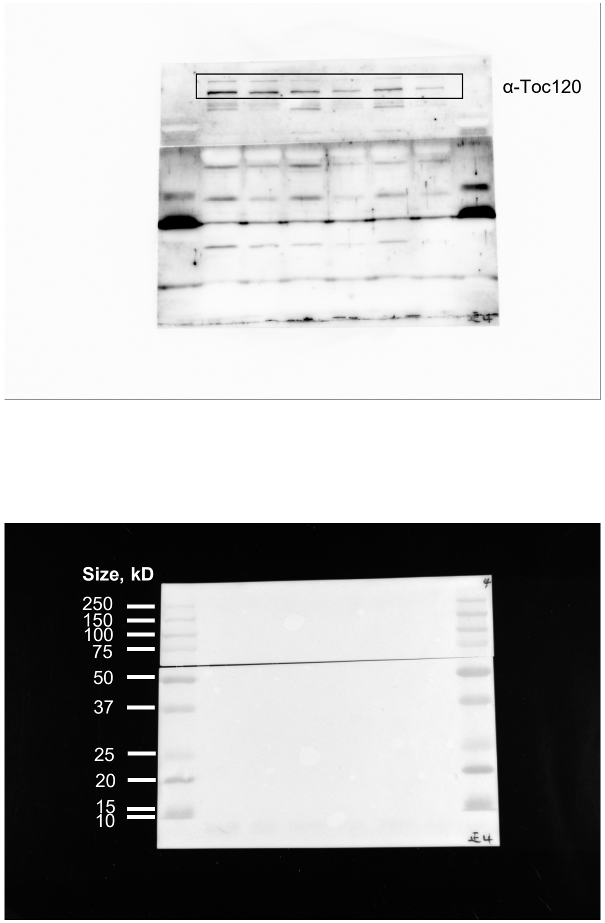


One single membrane but cut for probing with two different antibodies.
The lower panel was not included in Fig. 7d.

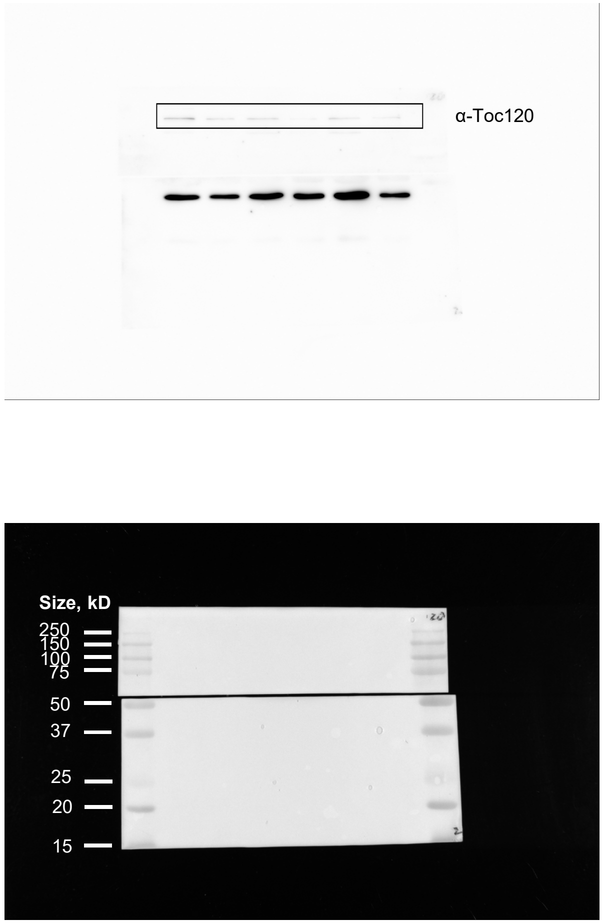


One single membrane but cut for probing with two different antibodies.
The lower panel was not included in Fig. 7d.

Note: Replicate results using identical sample loadings for quantification purposes, for Toc120, are shown; a longer exposure time was used for Toc120 quantification; the result shown in the Fig. 7c is marked with a dagger (†), although the exposure time may differ.

Multiple exposure times were recorded in each case, but for simplicity of presentation just a single exposure time is shown here.

**Fig. 7c,d continued**

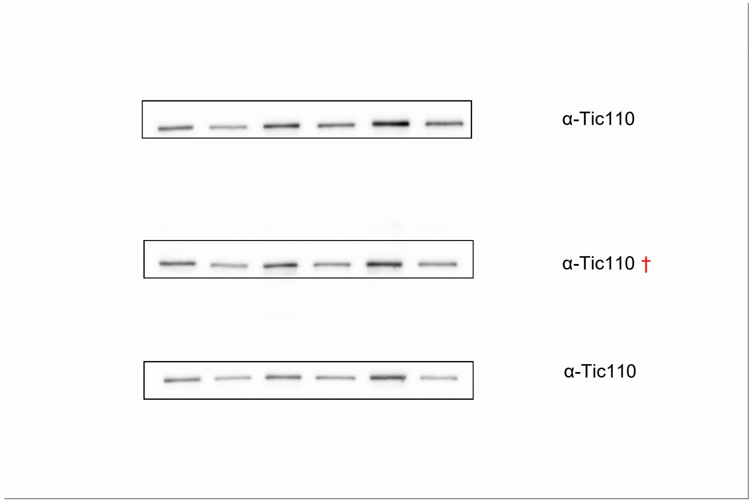


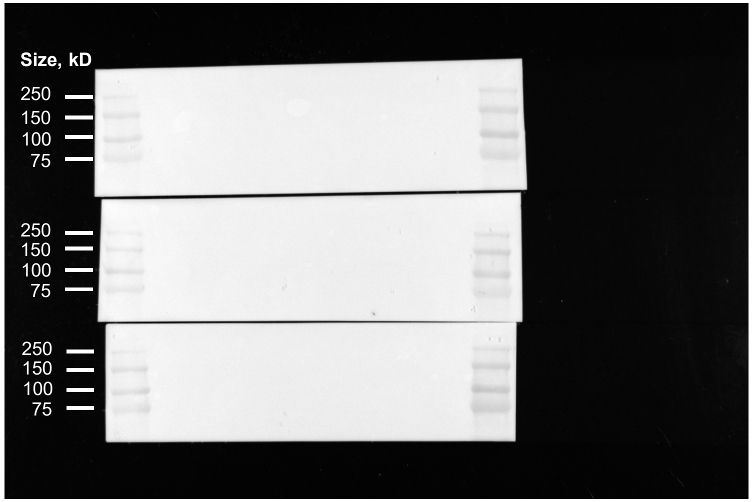


Three cropped membranes loaded with the same samples were probed with Tic110 antibody.

Note: Replicate results using identical sample loadings for quantification purposes, for Tic110, are shown; the result shown in Fig. 7c is marked with a dagger (†), although the exposure time may differ. Multiple exposure times were recorded in each case, but for simplicity of presentation just a single exposure time is shown here.

## Fig. 7c,d continued

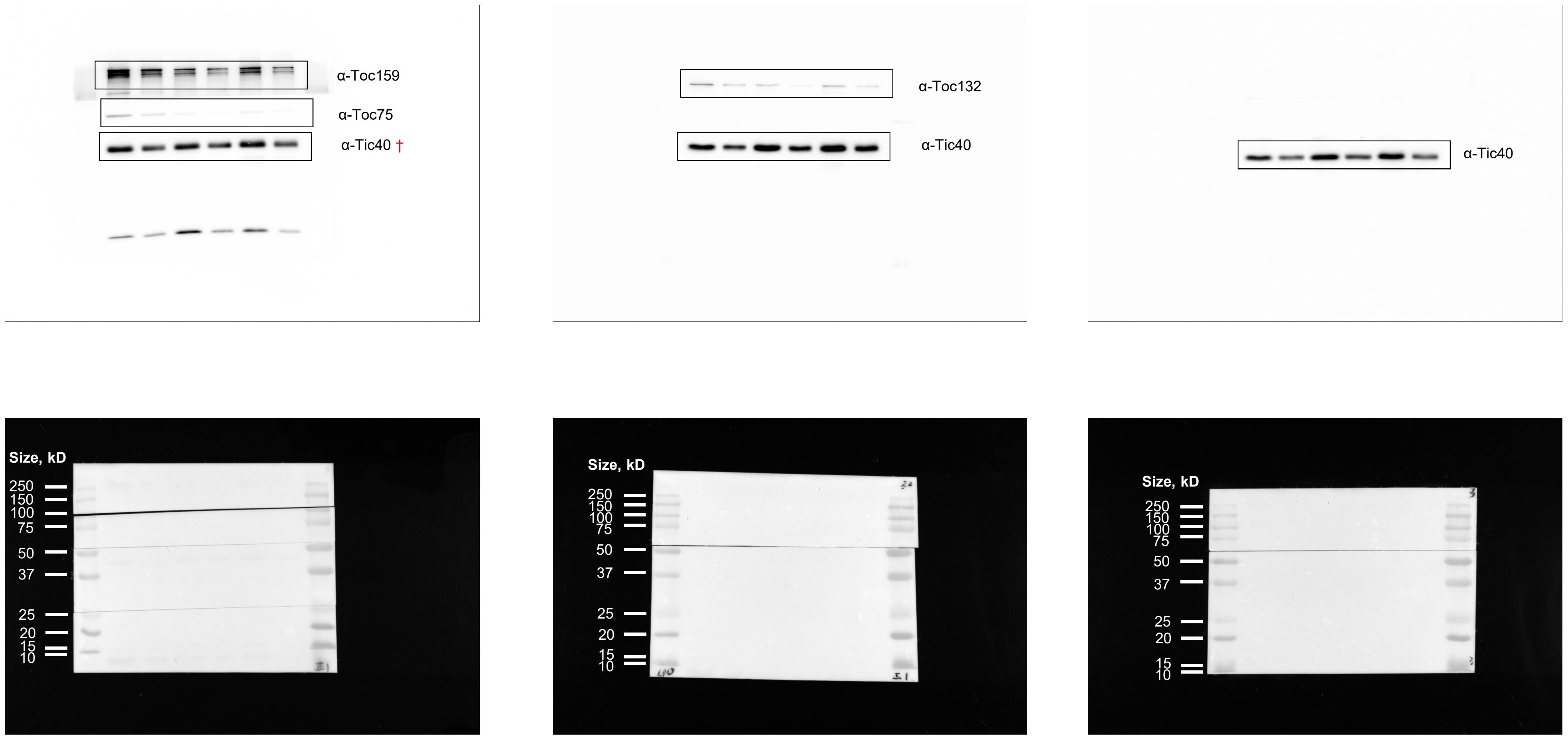


One single membrane but cut for probing with four different antibodies. The lower panel was not included in Fig. 7d.

One single membrane but cut for probing with two different antibodies.

One single membrane but cut for probing with two different antibodies.

Note: Replicate results using identical sample loadings for quantification purposes, for Tic40, are shown; the result shown in Fig. 7c is marked with a dagger (†), although the exposure time may differ.

Multiple exposure times were recorded in each case, but for simplicity of presentation just a single exposure time is shown here.

**Fig. 7e**

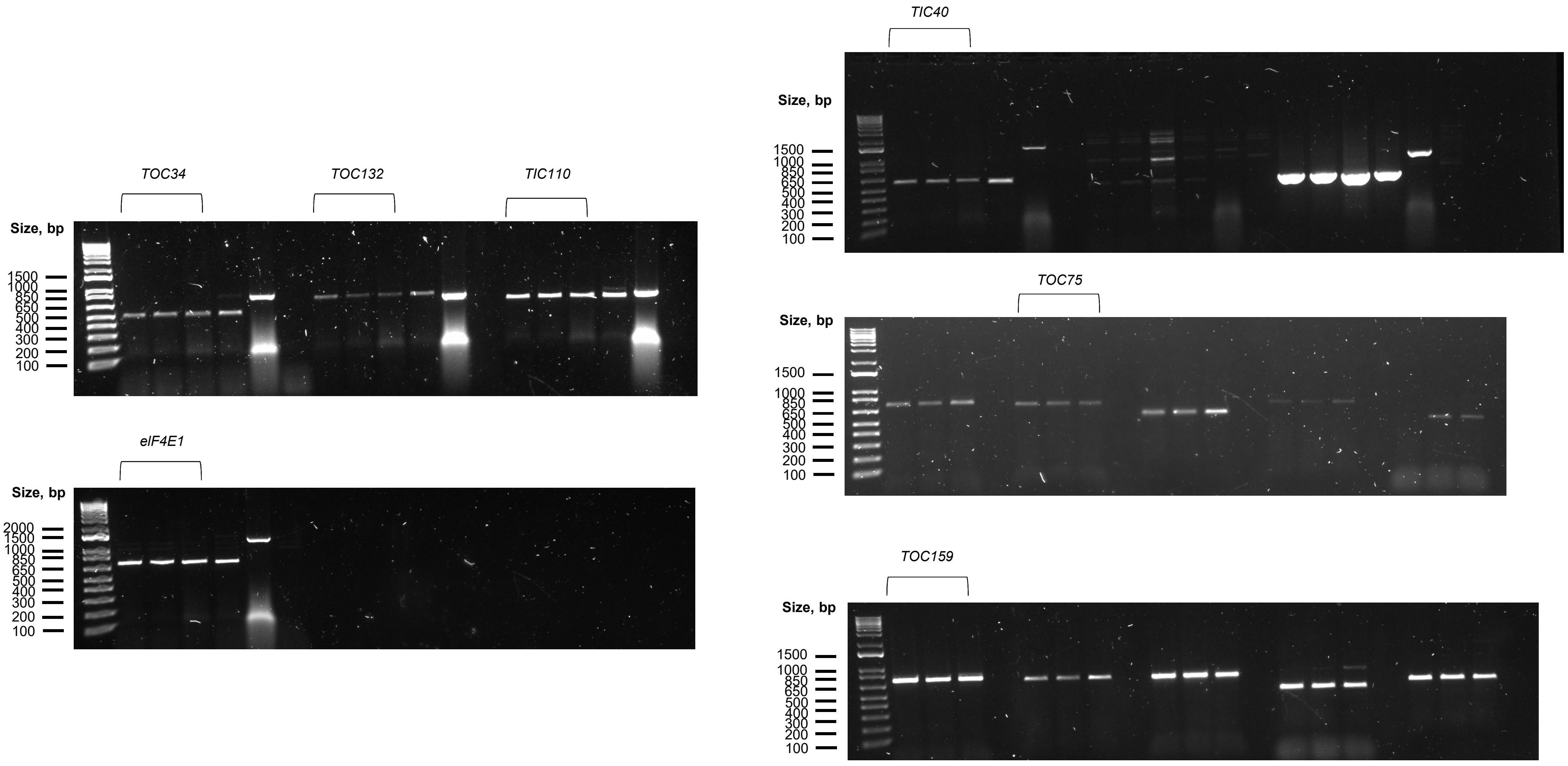
TOC34
TOC132
TIC110
Size, bp
1500
1000
850
650
500
400
300
200
100
eIF4E1
Size, bp
2000
1500
1000
850
650
500
400
300
200
100
TIC40
Size, bp
1500
1000
850
650
500
400
300
200
100
TOC75
Size, bp
1500
1000
850
650
500
400
300
200
100
TOC159
Size, bp
1500
1000
850
650
500
400
300
200
100
