# Supplementary material for: Recruitment of Cdc48 to chloroplasts by a UBX-domain protein in chloroplast-associated protein degradation
Source: Nat Plants. 2024 Aug 19;10(9):1400–17. doi: 10.1038/s41477-024-01769-x (PMC11410653; doi:10.1038/s41477-024-01769-x)
Supplement: Supplementary file 9 — Unprocessed western blots. [file 41477_2024_1769_MOESM9_ESM.pdf]

Fig. 8f,g

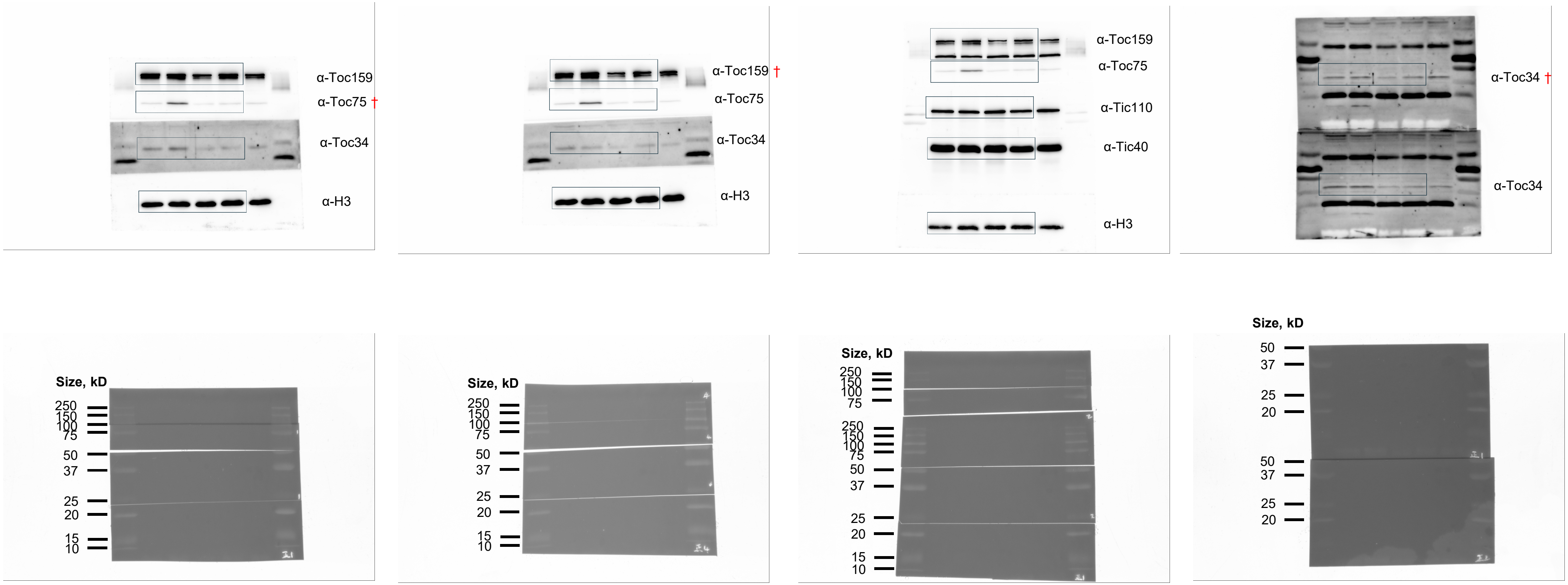

One single membrane but cut for probing with four different antibodies.

One single membrane but cut for probing with four different antibodies.

The two cropped membranes at the top were probed with two different antibodies, as indicated.

Below, one single membrane was cut for probing with three different antibodies.

Two cropped membranes loaded with the same samples were reprobed with Toc34 antibody.

Note: Replicate results using identical sample loadings for quantification purposes, for Toc159 and Toc34, are shown; a shorter exposure time was used for Toc159 quantification; the results shown in Fig. 8f are marked with a dagger (†), although the exposure times may differ.

Replicate results using identical sample loadings for quantification purposes, for Toc75, are shown here and on the following page; a longer exposure time was used for Toc75 quantification; the result shown in Fig. 8f is marked with a dagger (†), although the exposure time may differ.

Multiple exposure times were recorded in each case, but for simplicity of presentation just a single exposure time is shown here.

Fig. 8f,g continued

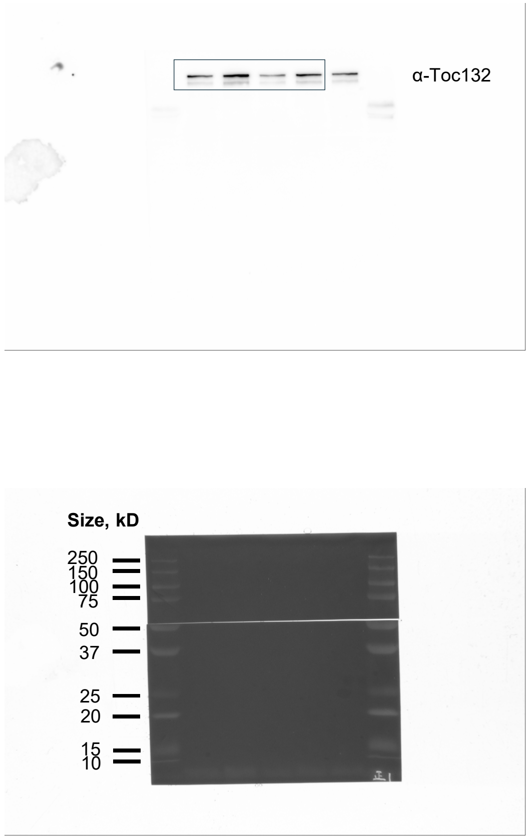

One single membrane but cut for probing with two different antibodies.

The signal for α-Toc34 was too weak so the membrane was reprobed with different Toc34 antibody (see previous page).

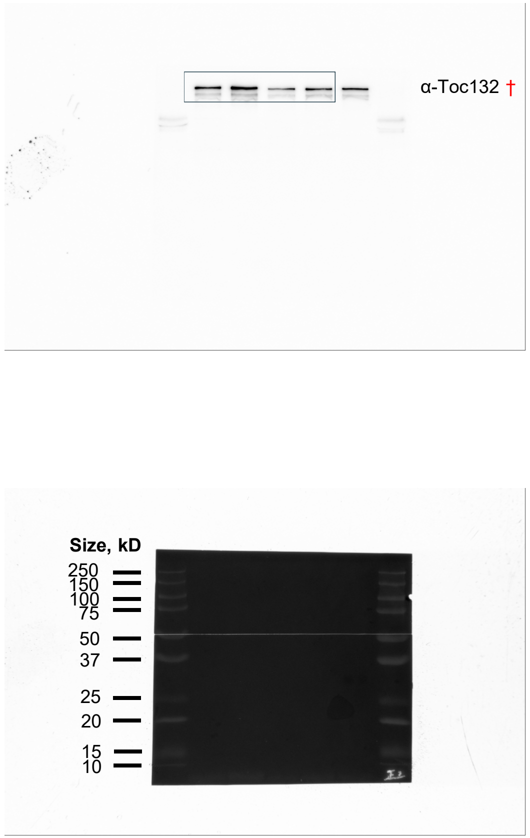

One single membrane but cut for probing with two different antibodies.

The signal for α-Toc34 was too weak so the membrane was reprobed with different Toc34 antibody (see previous page).

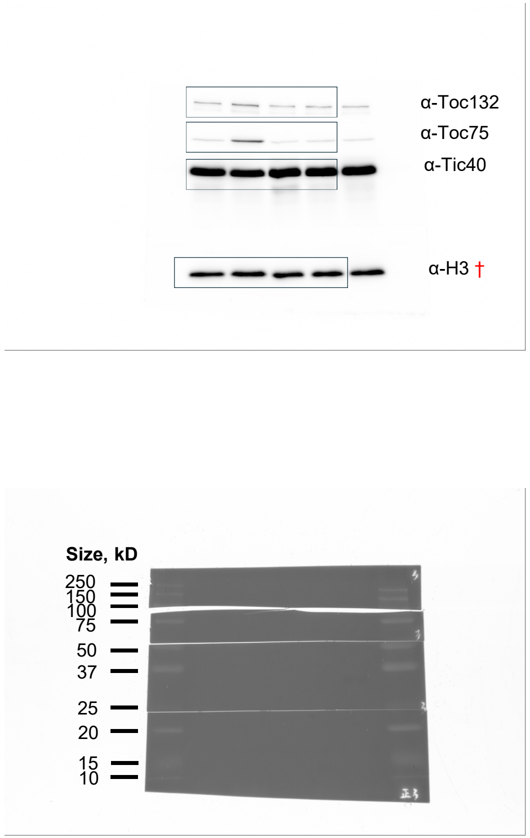

One single membrane but cut for probing with four different antibodies.

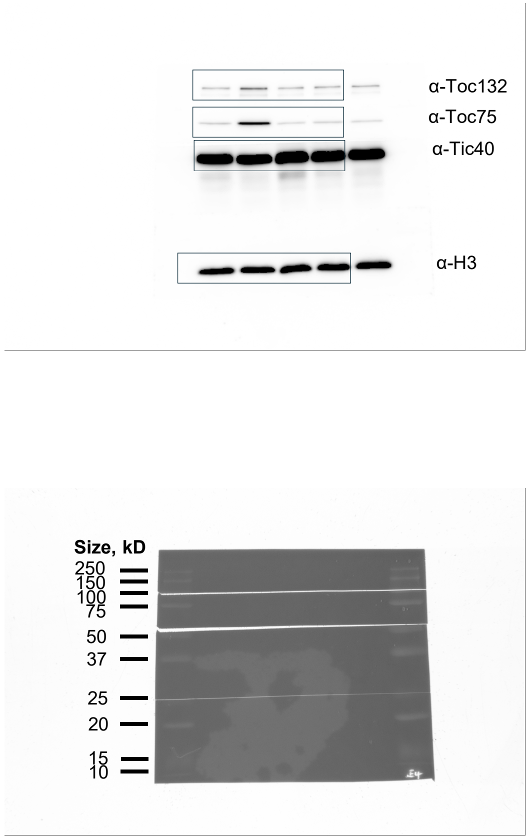

One single membrane but cut for probing with four different antibodies.

Note: Replicate results using identical sample loadings for quantification purposes, for Toc132, are shown; a longer exposure time was used for Toc132 quantification; the result shown in the Fig. 8f is marked with a dagger (†), although the exposure time may differ.

Replicate results using identical sample loadings for quantification purposes, for H3, are shown here, on the previous page, and on the following page; the result shown in the Fig. 8f is marked with a dagger (†), although the exposure time may differ.

Multiple exposure times were recorded in each case, but for simplicity of presentation just a single exposure time is shown here.

Fig. 8f,g continued

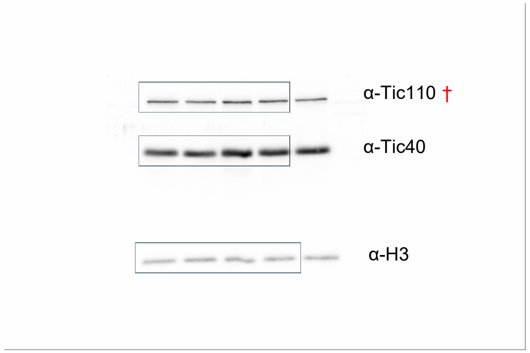

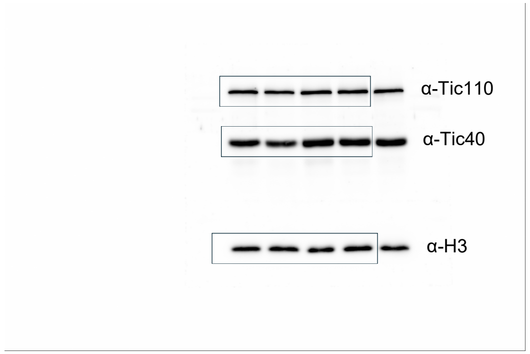

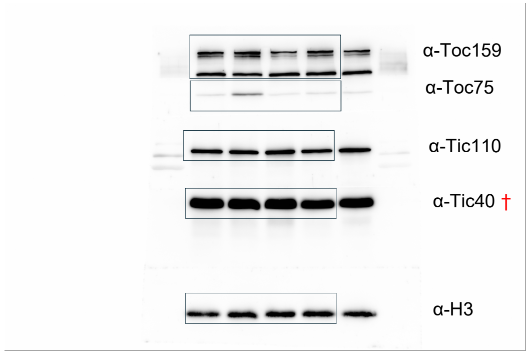

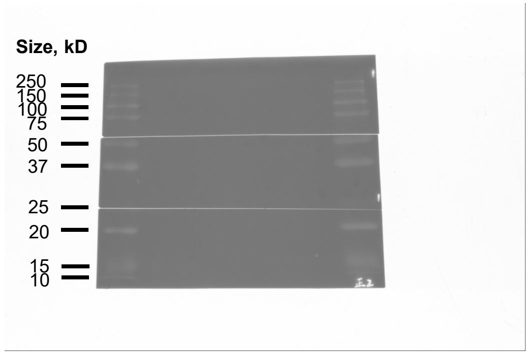

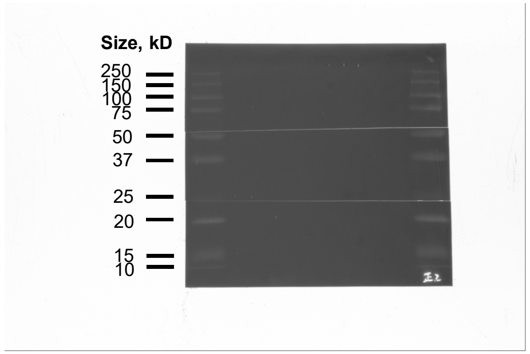

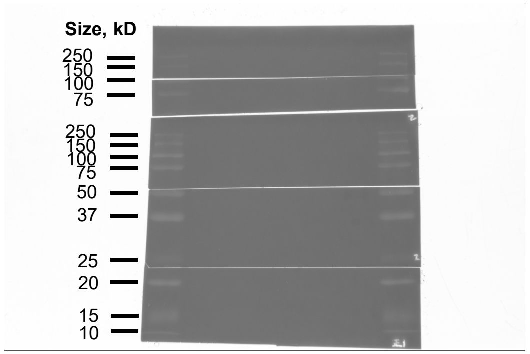

One single membrane but cut for probing with three different antibodies.

One single membrane but cut for probing with three different antibodies.

The two cropped membranes at the top were probed with two different antibodies, as indicated.

Below, one single membrane was cut for probing with three different antibodies.

Note: Replicate results using identical sample loadings for quantification purposes, for Tic110 and Tic40, are shown here and on the previous page; a shorter exposure time was used for Tic40 quantification; the results shown in Fig. 8f are marked with a dagger (†), although the exposure times may differ.

Multiple exposure times were recorded in each case, but for simplicity of presentation just a single exposure time is shown here.
