# Supplementary figures and images for: Recruitment of Cdc48 to chloroplasts by a UBX-domain protein in chloroplast-associated protein degradation
Source: Nat Plants. 2024 Aug 19;10(9):1400–17. doi: 10.1038/s41477-024-01769-x (PMC11410653; doi:10.1038/s41477-024-01769-x)

Extended data Fig. 3b

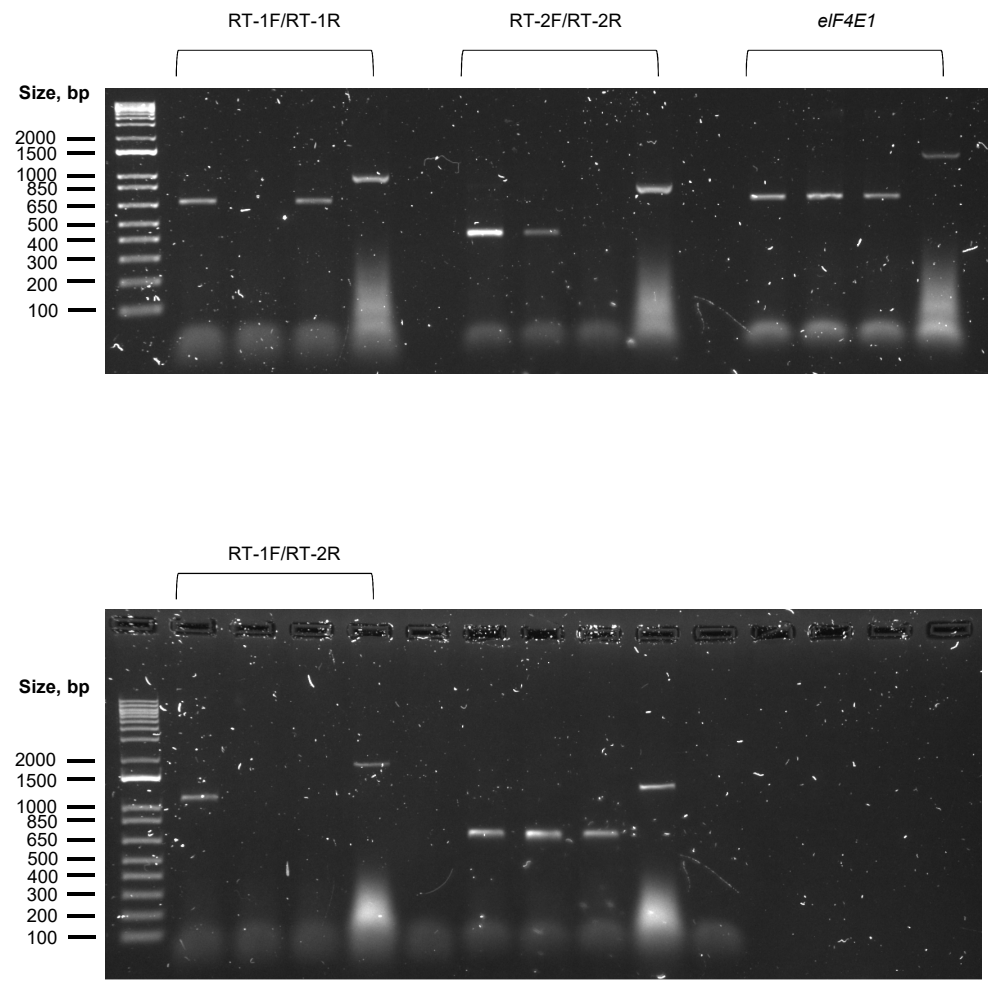

Supplement: Supplementary file 10 — Unprocessed gels. [file 41477_2024_1769_MOESM10_ESM.pdf]

Extended data Fig. 8b

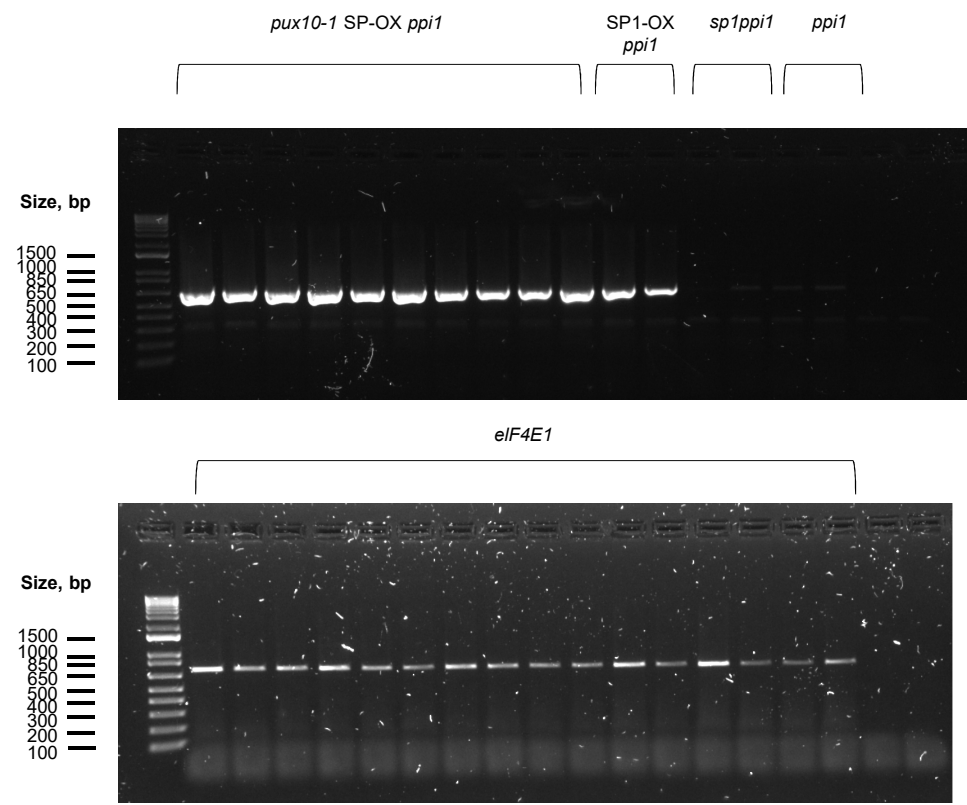

Supplement: Supplementary file 11 — Unprocessed gels. [file 41477_2024_1769_MOESM11_ESM.pdf]
